# Discovery of a pre-vein progenitor that requires VEGF/ERK inhibition to complete vein differentiation

**DOI:** 10.1101/2025.10.11.681838

**Published:** 2025-12-04

**Authors:** Lay Teng Ang, Sherry Li Zheng, Kevin J. Liu, Anastasiia Masaltseva, June Winters, Isabel von Creytz, Sawan K. Jha, Qingqing Yin, Crystal Qian, Xiaochen Xiong, Amir Dailamy, Ellie Xi, Juan C. Alcocer, Daniel W. Sorensen, Richard She, Karina Smolyar, Dorota Szumska, Svanhild Nornes, Renata M. Martin, Benjamin J. Lesch, Nicole K. Restrepo, Wenfei Sun, Jonathan S. Weissman, Heiko Lickert, Matthew H. Porteus, Mark A. Skylar-Scott, Christian Mosimann, Saulius Sumanas, Sarah De Val, Joseph B. Prescott, Kristy Red-Horse, Kyle M. Loh

**Affiliations:** 1 Institute for Stem Cell Biology & Regenerative Medicine, Stanford University, Stanford, CA, USA; 2 Department of Urology, Stanford University, Stanford, CA, USA; 3 Department of Developmental Biology, Stanford University, Stanford, CA, USA; 4 Department of Biology, Howard Hughes Medical Institute, Stanford University, Stanford, CA, USA; 5 Center for Biological Threats and Special Pathogens, Robert Koch Institute, Berlin, Germany; 6 School of Biological Sciences, Nanyang Technological University, Singapore; 7 Whitehead Institute for Biomedical Research, Department of Biology, Howard Hughes Medical Institute, Massachusetts Institute of Technology, Cambridge, MA, USA; 8 Institute of Developmental and Regenerative Medicine, Department of Physiology, Anatomy and Genetics, University of Oxford, Oxford, United Kingdom; 9 Department of Pediatrics, Stanford University, Stanford, CA, USA; 10 Department of Pathology and Cell Biology, University of South Florida, Tampa, Florida, USA; 11 Division of Endocrinology, Department of Medicine, Stanford University, Stanford, CA, USA; 12 Institute of Diabetes and Regeneration Research, Helmholtz Center Munich, Neuherberg, Germany; 13 German Center for Diabetes Research, Neuherberg, Germany; 14 School of Medicine, Technical University of Munich, Munich, Germany; 15 Department of Bioengineering, Basic Science and Engineering Initiative, Children’s Heart Center, Chan Zuckerberg Biohub, Stanford University, Stanford, CA, USA; 16 Department of Pediatrics, Section of Developmental Biology, University of Colorado School of Medicine, Anschutz Medical Campus, Aurora, CO, USA; 17 Ludwig Institute for Cancer Research Ltd, Nuffield Department of Medicine, University of Oxford, Oxford, United Kingdom; 18 Current address: Stately Bio, Palo Alto, CA, USA (S.L.Z.), Department of Biology, Stanford University, Stanford, CA, USA (X.X.), Replay Bio, San Diego, CA, USA (R.M.M.), Department of Surgery, University of California San Francisco, San Francisco, USA (B.J.L.); 19 These authors contributed equally to this work; 20 These authors contributed equally to this work

## Abstract

Despite substantial insight into mechanisms underlying arterial blood vessel development, multiple aspects of vein development remain elusive, including vein-determining extracellular signals and cell-fate trajectories^1–10^. One might expect arteries and veins to arise simultaneously during development, as both are needed for a functional circulatory system. Nevertheless, arteries often precede veins *in vivo*, as exemplified by the first intraembryonic blood vessels^11–17^. Here we present a model of vein differentiation that answers longstanding questions in the field. By reconstituting human vein endothelial cell (EC) differentiation from mesoderm *in vitro*, we discovered that vein development unfolds in two steps driven by opposing signals. First, VEGF is necessary to differentiate mesoderm into “pre-vein” ECs—a newly-defined intermediate state—and to endow endothelial identity. Second, once cells have acquired pre-vein EC identity, VEGF/ERK inhibition is necessary to specify vein ECs. Pre-vein ECs co-expressed certain arterial (SOX17) and venous (APLNR) markers and harbored poised chromatin at future venous genes. However, VEGF/ERK inhibition was necessary to activate poised venous genes (e.g., *NR2F2*), and for pre-vein ECs to complete venous differentiation. Intersectional lineage tracing supported a pre-vein intermediate step *in vivo*: early Sox17+ Aplnr+ ECs also formed veins in mouse embryos. We leveraged this developmental knowledge for disease modeling by differentiating human pluripotent stem cells into artery and vein ECs, and comparing their responses to Ebola and Andes viruses under biosafety-level-4 containment. Artery and vein ECs responded divergently to the same virus, thus revealing that developmentally specified cell identity impacts viral infection. Taken together, we propose a two-step model for vein development wherein VEGF first differentiates mesoderm into pre-vein ECs, but subsequent VEGF/ERK inhibition generates vein ECs. VEGF activation is thought to be broadly essential for vascular development^6,18^, and thus our discovery that VEGF/ERK inhibition specifies vein identity has potential implications for understanding current therapies that either activate or inhibit VEGF signaling^19,20^.

Endothelial cells (ECs) comprise the inner lining of blood vessels, and there are multiple subtypes of ECs, including artery, vein, capillary, and lymphatic ECs; each executes specialized functions and expresses different genes^1–10^. For instance, artery ECs express NOTCH ligands that specify the smooth muscle cells that ensconce arteries^21^. By contrast, vein ECs can construct valves that prevent backward blood flow^22^. The prevailing model suggests that artery ECs express SOXF family transcription factors (SOX7, SOX17, and SOX18) that impart arterial identity^23–29^. Conversely, vein ECs express the transcription factor NR2F2 and transmembrane proteins APLNR/APJ and FLRT2^14,30–35^.

Pathbreaking work has discovered key precepts of artery EC development^3–10^: VEGF/ERK activation^36–38^, NOTCH activation^39–43^, and cell-cycle arrest^33,44,45^ all drive arterialization. Nevertheless, many mysteries continue to surround the development of vein ECs^3,4^. For instance, one might assume that arteries and veins should arise contemporaneously, as both are required to form a functional circulatory system. Nevertheless, during the development of the first intraembryonic blood vessels, arteries emerge earlier than veins, as shown by anatomy and molecular markers in mouse and zebrafish embryos^11–17^. Why veins should emerge after arteries remains a longstanding curiosity. This piques questions about the earliest steps of venous development and the exact identity of the progenitor cells that build veins.

The extracellular signals that specify venous identity also warrant further investigation^3,4^. The transcription factor NR2F2 is a master regulator of vein EC identity^32^, but the upstream signals that ignite NR2F2 expression remain largely unknown^3,4^. VEGF is a master regulator of EC identity^6,18–20,46,47^, but has paradoxical roles in venous development. On one hand, VEGF is *required* for vein EC formation, as ECs are absent in *Vegfr2*^−/−^ mouse embryos^18^. On the other hand, VEGF also *represses* vein formation, as it instructs arterial identity in zebrafish embryos^36,37^. How can VEGF both promote and inhibit vein EC specification during vascular development?

Here we introduce a conceptual model of human vein EC differentiation that addresses certain mysteries surrounding vein development. We unveil two separable steps of vein differentiation, driven by opposing extracellular signals. First, VEGF *activation* differentiates lateral mesoderm into a newly discovered “pre-vein” EC intermediate, which co-expresses “arterial” marker SOX17 and “venous” marker APLNR. Future venous genes are poised at the chromatin level within pre-vein ECs. Subsequently, VEGF/ERK *inhibition* installs fully-fledged venous identity, and converts these poised venous genes into an active state. Intersectional genetic tracing confirmed that *Sox17*+ *Aplnr*+ ECs form vein ECs *in vivo*.

The discovery of pre-vein ECs may explain several longstanding curiosities in vascular development, including why veins tend to emerge after arteries *in vivo*^11–17^. Our model also reconciles why VEGF/ERK signaling can paradoxically both promote^18^ and inhibit^36–38^ vein EC development: the same signal executes both functions, at different times. The timing of an extracellular signal is of paramount importance, as it is interpreted in a temporally dynamic way. Our discovery that VEGF/ERK inhibition is required for vein EC specification also has potential implications for VEGF pathway inhibitors, which often exert mixed or modest clinical effects on cancer progression^19,20^. While VEGF inhibitors were designed to block tumor angiogenesis^19,20^, perhaps in certain contexts they might confer venous identity upon ECs; the latter phenomenon may contribute to VEGF inhibitor resistance. Finally, we applied developmental knowledge of the pre-vein state, and differentiated hPSCs into vein and artery ECs for disease modeling. Vein ECs responded differently to Ebola and Andes virus infection than artery ECs. To the best of our knowledge, this represents the first direct comparison of hemorrhagic fever viruses from two different families under BSL4 containment, thereby enriching comparative virology.

## RESULTS

### Discovery of “pre-vein” ECs that arise during human vascular differentiation *in vitro*

**Extended Data Figure 1:**
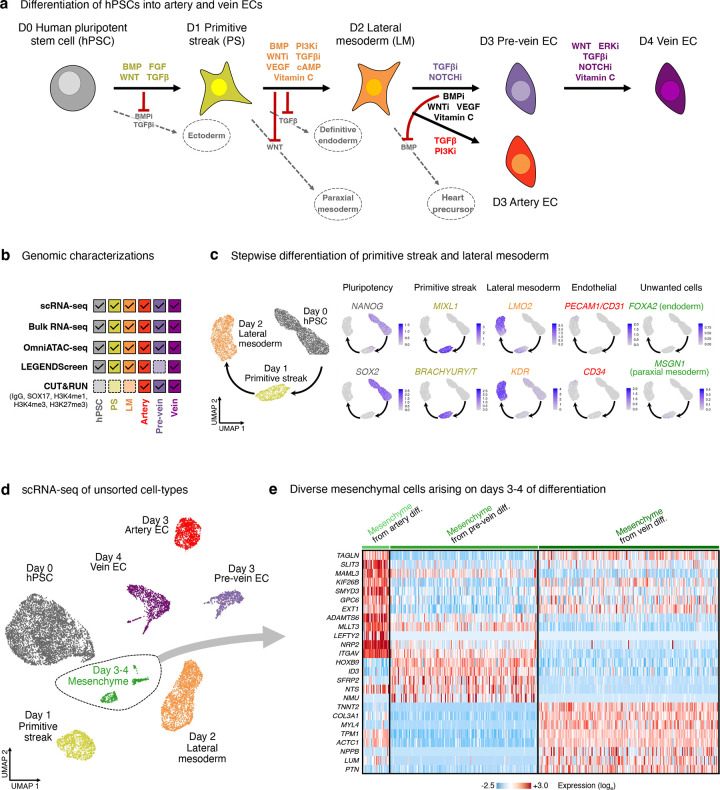
Cellular diversity during early steps of hPSC differentiation A) Summary of hPSC differentiation approach^48^. i: inhibition. B) Summary of gene expression, chromatin state, and cell-surface marker profiling conducted as part of this study. Dotted lines indicate that profiling was not conducted on a given cell-type. C) Gene expression on days 0, 1, and 2 of H1 hPSC differentiation, as detected by scRNAseq. D) scRNAseq of day-0 H1 hPSCs that were differentiated into day-1 primitive streak, day-2 lateral mesoderm, day-3 artery ECs, day-3 pre-vein ECs, and day-4 vein ECs. None of cell populations shown here were FACS purified and consequently, mesenchymal cells were detected alongside artery, pre-vein, and vein ECs on days 3–4 of differentiation. The same day-0 hPSC, day-1 primitive streak, and day-2 primitive streak scRNAseq datasets were also used in Fig. 1A; however, FACS-purified downstream EC populations are shown in Fig. 1A. E) scRNAseq of H1 hPSC-derived mesenchymal cells that arose alongside day-3 artery ECs, day-3 pre-vein ECs, and day-4 vein ECs. Differentially expressed genes that distinguish these three different types of mesenchyme are shown. Diff: differentiation.

**Figure 1:**
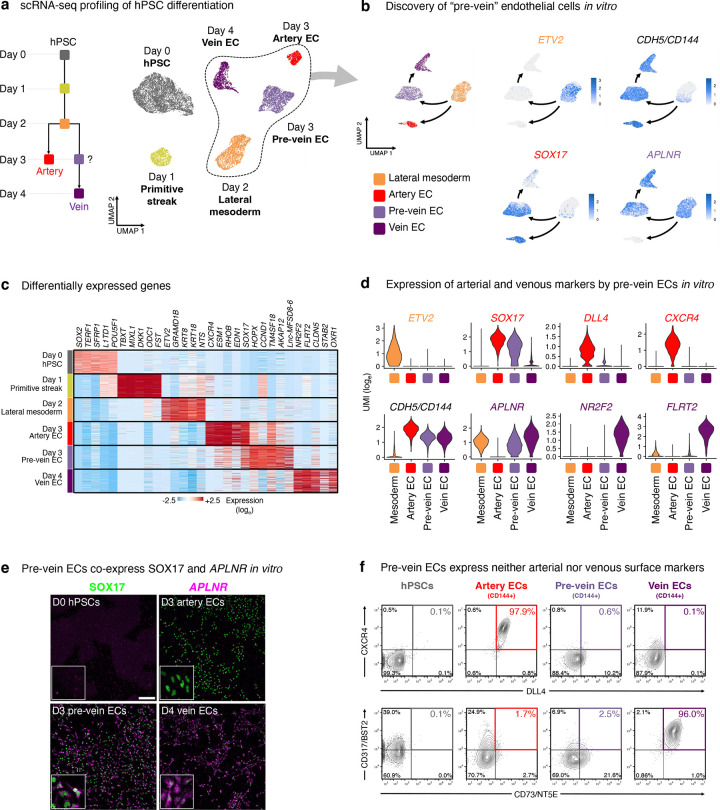
A roadmap for human arteriovenous differentiation reveals pre-vein endothelial cells A) scRNA-seq of day-0 H1 hPSCs, day-1 primitive streak, day-2 lateral mesoderm, CD144+ FACS-purified day-3 artery ECs, CD144+ FACS-purified day-3 pre-vein ECs, and CD144+ FACS-purified day-4 vein ECs. B) Differentially expressed genes that distinguish each hPSC-derived cell-type, as detected by scRNAseq. scRNA-seq was performed on day-0 H1 hPSCs, day-1 primitive streak, day-2 lateral mesoderm, CD144+ FACS-purified day-3 artery ECs, CD144+ FACS-purified day-3 pre-vein ECs, and CD144+ FACS-purified day-4 vein ECs. C) scRNA-seq of day-2 lateral mesoderm, CD144+ FACS-purified day-3 artery ECs, CD144+ FACS-purified day-3 pre-vein ECs, and CD144+ FACS-purified day-4 vein ECs. D) scRNA-seq of day-2 lateral mesoderm, CD144+ FACS-purified day-3 artery ECs, CD144+ FACS-purified day-3 pre-vein ECs, and CD144+ FACS-purified day-4 vein ECs. Gene expression is depicted in log_e_ unique molecular identifier (UMI) counts. E) Combined immunostaining for SOX17 protein and HCR3 *in situ* hybridization for *APLNR* mRNA in the indicated H1 hPSC-derived cell-types. Scale: 200 μm. F) Flow cytometry of H1 CRISPRi-expressing hPSCs, day-3 artery ECs, day-3 pre-vein ECs, and day-4 vein ECs. Day 3–4 populations were pre-gated on the CD144+ EC subset before depicting marker expression.

At 24 hour increments during hPSC differentiation into artery and vein ECs^48^, we mapped gene expression (through single-cell [scRNAseq] and bulk-population RNA-sequencing), chromatin accessibility (using OmniATACseq^49^), and cell-surface marker expression (through high-throughput screening of 332 cell-surface markers) (Fig. 1A, Extended Data Fig. 1A–E, Table S1–S2). We created an interactive web browser to explore scRNAseq data of each differentiation stage (https://anglab.shinyapps.io/artery-vein-scrna-seq/).

**Extended Data Figure 2:**
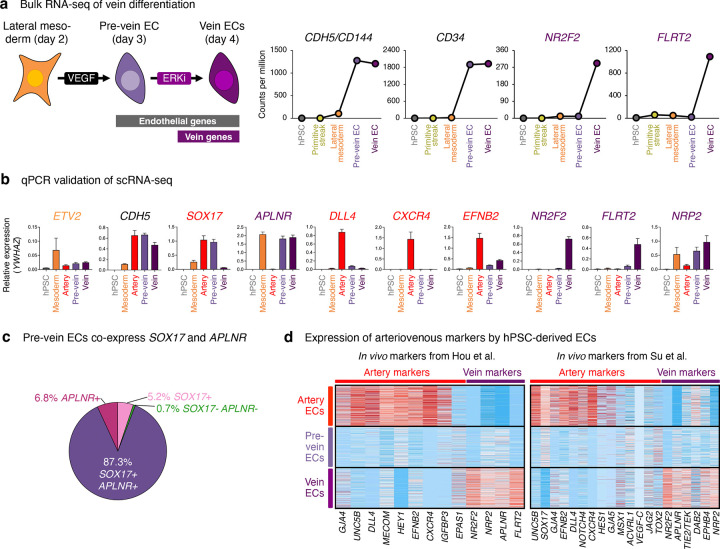
Characterization of pre-vein endothelial cells *in vitro* A) Bulk RNA-seq of the indicated H1 hPSC-derived cell-types. FACS was used to purify CD144+ pre-vein and vein ECs for RNA-seq. i: inhibition. B) qPCR of the indicated H1 hPSC-derived cell-types. qPCR data normalized to reference gene YWHAZ (i.e., YWHAZ levels = 1.0). Error bars: standard deviation (SD). C) scRNAseq of H1 hPSC-derived day-3 pre-vein EC populations, showing the percentage of cells that expressed APLNR and/or SOX17 mRNAs. D) scRNAseq of FACS-purified CD144+ DLL4+ CD73lo/− day-3 artery ECs, CD144+ day 3 pre-vein ECs, and CD144+ DLL4− CD73hi day-4 vein ECs generated from H1 hPSCs. In each of these in vitro cell-types, expression of in vivo arterial and venous markers reported by Hou et al., 202214 and Su et al., 201833 is shown

Investigating stepwise molecular changes during venous differentiation in detail, we discovered an unexpected intermediate with unique gene expression, chromatin, and cell-surface marker signatures—which we term “pre-vein” ECs—that preceded the emergence of vein ECs. Day-2 lateral mesoderm expressed the angioblast transcription factor *ETV2*^50^, but upon 24 hours of further differentiation, *ETV2* was silenced, whereupon EC surface markers (e.g., *CD144/VE-CADHERIN*) became expressed by day 3 (Fig. 1B–D, Extended Data Fig. 2A–B). However, mature venous markers *NR2F2* and *FLRT2* were only expressed by day 4 of vein differentiation (Fig. 1C–D, Extended Data Fig. 2A–B).

**Extended Data Figure 3:**
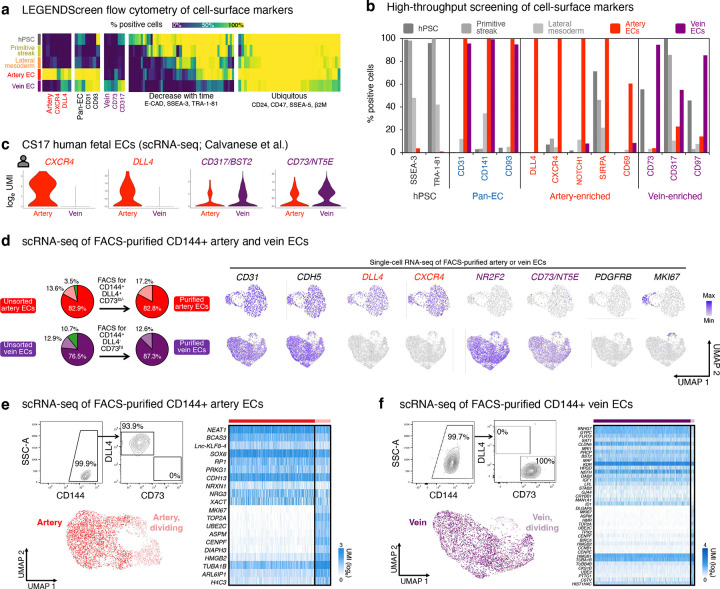
Cell-surface markers of human arteriovenous identity A) LEGENDScreen high-throughput flow cytometry of cell-surface markers in day-0 hPSCs, day-1 primitive streak, day-2 lateral mesoderm, day-3 artery ECs, and day-4 vein ECs. Day-3 artery and day-4 vein EC populations were pre-gated on the CD144+ EC subset before depicting marker expression B) LEGENDScreen high-throughput flow cytometry of cell-surface markers in day-0 hPSCs, day-1 primitive streak, day-2 lateral mesoderm, day-3 artery ECs, and day-4 vein ECs. Day-3 artery and day-4 vein EC populations were pre-gated on the CD144+ EC subset before depicting marker expression. C) scRNAseq of human Carnegie Stage 17 (CS17) fetal ECs. scRNAseq data were obtained from Calvanese et al., 202266. D) Left: Population heterogeneity of H1 hPSC-derived day-3 artery ECs and day-4 vein ECs, before and after FACS purification based on the indicated cell-surface marker combinations. Proportions of cell clusters in scRNAseq data are shown. Right: The copyright holder for this preprint bioRxiv preprint doi: https://doi.org/10.1101/2025.10.11.681838; this version posted December 4, 2025. (which was not certified by peer review) is the author/funder, who has granted bioRxiv a license to display the preprint in perpetuity. It is made available under a CC-BY 4.0 International license. scRNAseq of FACS-purified CD144+ DLL4+ CD73lo/− day-3 artery ECs and CD144+ DLL4− CD73hi day-4 vein ECs generated from H1 hPSCs. E) scRNAseq of FACS-purified CD144+ DLL4+ CD73lo/− day-3 artery ECs generated from H1 hPSCs. Left: FACS isolation strategy. Right: subclustering was performed to assess any potential population heterogeneity, and differentially expressed genes that distinguished cell subsets are shown. F) scRNAseq of FACS-purified CD144+ DLL4− CD73hi day-4 vein ECs generated from H1 hPSCs. Left: FACS isolation strategy. Right: subclustering was performed to assess any potential population heterogeneity, and differentially expressed genes that distinguished cell subsets are shown.

What is the identity of these incipient day-3 “pre-vein” ECs emerging during venous differentiation, prior to expression of fully-fledged venous markers by day 4? Curiously, CD144+ pre-vein ECs co-expressed the “arterial” marker *SOX17*^23–27^ and “venous” marker *APLNR*^30,31^ (Fig. 1B,D). By contrast, cells subjected to arterial differentiation solely expressed *SOX17*, but not *APLNR*, as expected^14,23–27,30–35^ (Fig. 1B,D). We confirmed that single pre-vein ECs co-expressed SOX17 and *APLNR*, as shown by combined *in situ* hybridization and immunostaining (Fig. 1E) and scRNAseq (Extended Data Fig. 2C). However, pre-vein ECs did not express additional markers of arterial (*GJA4/CX37*, *UNC5B*, *DLL4, MECOM, HEY1, EFNB2, CXCR4*, or *IGFBP3*), venous (*NR2F2*, *FLRT2*, or *NRP2*), or angioblast (*ETV2*) identity^14,33^ (Fig. 1D, Extended Data Fig. 2B,D). High-throughput profiling of cell-surface markers revealed that pre-vein ECs did not express markers of artery ECs (CXCR4+ DLL4+) or vein ECs (CD73+ CD317+)^51–53^, suggesting that pre-vein ECs can be classified as neither arterial nor venous (Extended Data Fig. 3A–F; Table S3). Having discovered pre-vein ECs, we subsequently explored the extracellular signals that drive entry into, and exit from, this intermediate state.

### Two steps of vein EC differentiation *in vitro*: VEGF/ERK activation, followed by inhibition

**Extended Data Figure 4:**
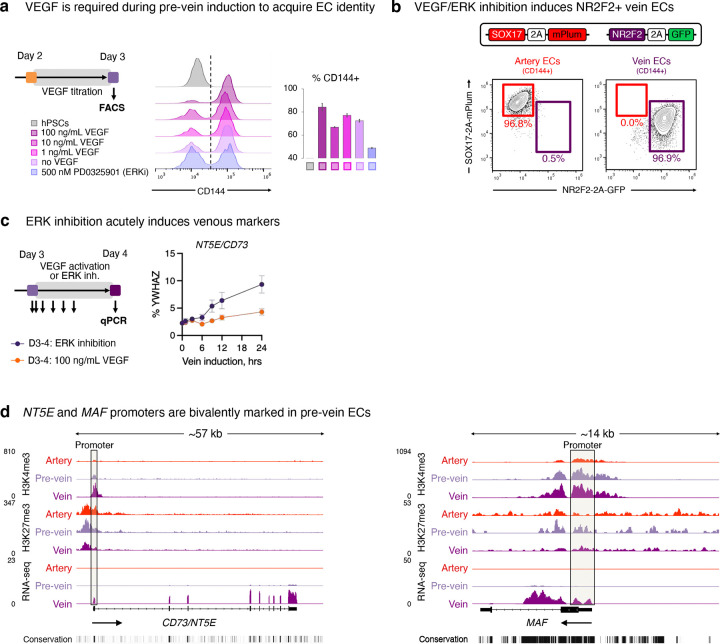
Generation and characterization of pre-vein endothelial cells *in vitro* A) First, H1 hPSCs were differentiated into day-2 lateral mesoderm^48^. Then, lateral mesoderm was then treated with different doses of VEGF pathway modulators (VEGF [0–100 ng/mL] or ERK inhibitor [ERKi; PD0325901, 500 nM]) alongside other pre-vein EC-inducing signals (TGFβ inhibitor + NOTCH inhibitor + BMP inhibitor + WNT inhibitor + Vitamin C) for 24 hours. Flow cytometry was conducted on day 3 of hPSC differentiation. This revealed that high VEGF for 24 hours is required during pre-vein EC specification to efficiently generate ECs by day 3. B) Flow cytometry of H1 *SOX17-2A-mPlum; NR2F2-2A-GFP* hPSCs differentiated into day-3 artery ECs or day-5 vein ECs. Day-4 vein ECs were maintained in the same vein induction media until day 5. Day 3–5 populations were pre-gated on the CD144+ EC subset before depicting marker expression. C) First, H1 hPSCs were differentiated into day-3 pre-vein ECs. Then, pre-vein ECs were then treated with different doses of VEGF pathway modulators (VEGF [0–100 ng/mL] or ERK inhibitor [ERKi; PD0325901, 500 nM]) alongside other vein EC-inducing signals (TGFβ inhibitor + NOTCH inhibitor + WNT agonist + Vitamin C) for 1–24 hours. qPCR was conducted on day 4 of hPSC differentiation, and expression is normalized to the sample with the highest expression. This revealed that ERK inhibition for 12 hours significantly upregulated venous marker expression. D) CUT&RUN profiling of H3K4me3 and H3K27me3 and bulk RNA-seq H1 hPSC-derived day-3 artery ECs, day-3 pre-vein ECs, and day-4 vein ECs.

**Figure 2:**
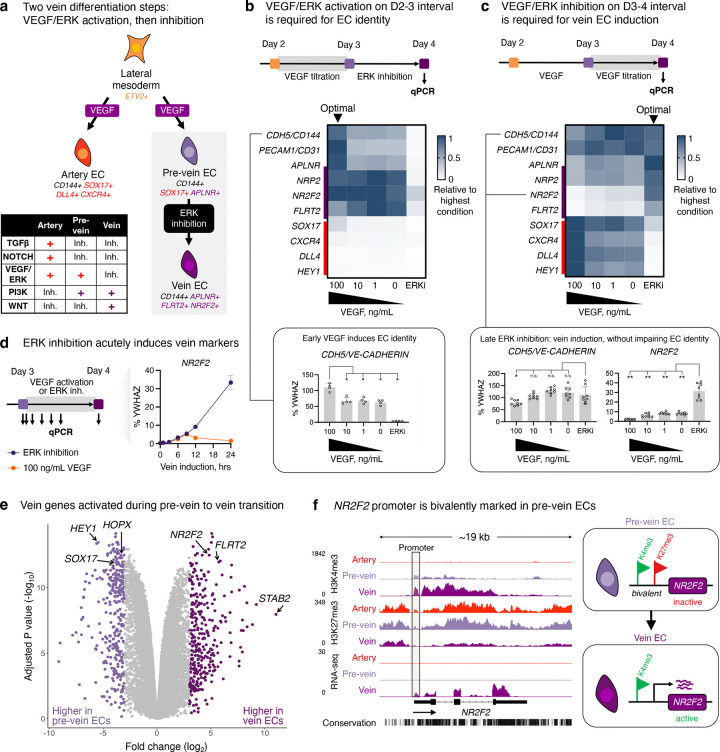
Two separable steps of vein differentiation driven by temporally dynamic VEGF/ERK activation, followed by inhibition A) Summary of the present study. B) First, H1 hPSCs were differentiated into day-2 lateral mesoderm^48^. Then, lateral mesoderm was then treated with different doses of VEGF pathway modulators (VEGF [0–100 ng/mL] or ERK inhibitor [ERKi; PD0325901, 500 nM]) alongside other pre-vein EC-inducing signals (TGFβ inhibitor + NOTCH inhibitor + BMP inhibitor + WNT inhibitor + Vitamin C) for 24 hours. Subsequently, cells were subject to vein EC differentiation for 24 hours. qPCR was conducted on day 4 of hPSC differentiation. In heatmaps, expression is normalized to the sample with the highest expression in either panel B or C. This revealed that high VEGF for 24 hours is required during pre-vein EC specification to subsequently generate vein ECs by day 4. Statistics: Wilcoxon rank sum test. Error bars: standard deviation (SD). *P<0.05. C) First, H1 hPSCs were differentiated into day-3 pre-vein ECs. Then, pre-vein ECs were then treated with different doses of VEGF pathway modulators (VEGF [0–100 ng/mL] or ERK inhibitor [ERKi; PD0325901, 500 nM]) alongside other vein EC-inducing signals (TGFβ inhibitor + NOTCH inhibitor + WNT agonist + Vitamin C) for 24 hours. qPCR was conducted on day 4 of hPSC differentiation. In heatmaps, expression is normalized to the sample with the highest expression in either panel B or C. This revealed that ERK inhibition for 24 hours is required to generate day-4 vein ECs, and that after cells acquire endothelial identity, VEGF/ERK is dispensable for the continued expression of pan-EC markers. Statistics: Wilcoxon rank sum test. Error bars: SD. **P<0.01, *P<0.05, n.s.: not significant. D) First, H1 hPSCs were differentiated into day-3 pre-vein ECs. Then, pre-vein ECs were then treated with either VEGF (100 ng/mL) or ERK inhibitor (PD0325901, 500 nM) alongside other vein EC-inducing signals (TGFβ inhibitor + NOTCH inhibitor + WNT agonist + Vitamin C) for 1–24 hours, followed by qPCR. This revealed that ERK inhibition for 12 hours significantly upregulated venous marker expression. E) Bulk-population RNA-seq of FACS-purified CD144+ day-3 pre-vein ECs and day 4-vein ECs generated from H1 hPSCs. Differentially expressed genes are colored purple. F) CUT&RUN profiling of H3K4me3 and H3K27me3 and bulk RNA-seq H1 hPSC-derived day-3 artery ECs, day-3 pre-vein ECs, and day-4 vein ECs.

What extracellular signals specify venous identity? We tested different combinations of the developmental signals shown in Fig. 2A. This revealed that VEGF *activation* was required to drive day-2 lateral mesoderm differentiation into day-3 pre-vein ECs, alongside inhibition of the artery-specifying TGFβ and NOTCH pathways^39–43,48^ (Fig. 2B). During this first 24-hour interval, VEGF/ERK activation was crucial to generate ECs; in its absence, EC formation *in vitro* was significantly impaired (Fig. 2B, Extended Data Fig. 4A). The requirement for VEGF to differentiate lateral mesoderm into pre-vein ECs is consistent with how VEGF is required for EC emergence *in vivo*^18,36^.

While VEGF was essential to generate day-3 pre-vein ECs, our combinatorial testing of developmental signals unexpectedly revealed that VEGF/ERK *inhibition* was necessary for their further progression into day-4 vein ECs (Fig. 2C, Extended Data Fig. 4B). Upon VEGF/ERK inhibition, venous markers *NR2F2* and *FLRT2* sharply increased, and pre-vein marker *SOX17* was silenced (Fig. 2D–E, Extended Data Fig. 2A). 12 hours of VEGF/ERK inhibition sufficed to significantly activate *NR2F2* expression (Fig. 2D, Extended Data Fig. 4C). By contrast, continued VEGF activation at this stage was arterializing, and repressed venous markers (Fig. 2C–D), as observed in zebrafish embryos^36,37^. The requirement for VEGF/ERK inhibition to confer venous identity was surprising, as VEGF is typically thought to be broadly required for all ECs^6,18,36^. Nevertheless, after cells have acquired endothelial identity, we found that VEGF was dispensable for cells to retain endothelial character (Fig. 2C).

We hypothesized that pre-vein ECs represent an early intermediate in vein development wherein venous genes are poised for future activation at the chromatin level. To test this hypothesis, we mapped the genome-wide distribution of H3K4me1, H3K4me3, and H3K27me3 using CUT&RUN^54^. In pre-vein ECs, future venous genes such as *NR2F2*, *NT5E*, and *MAF* were not yet expressed, but nevertheless their promoter regions were bivalently marked by activation-associated H3K4me3 and repression-associated H3K27me3 (Fig. 2F, Extended Data Fig. 4D). Such promoter bivalency can denote genes poised for future activation in anticipation of future developmental decisions^55^. VEGF/ERK inhibition transitioned the promoter elements of these venous genes from bivalency (H3K4me3+ H3K27me3+) to full activation (H3K4me3+ H3K27me3−), which was accompanied by transcription (Fig. 2F, Extended Data Fig. 4D).

We thus propose two sequential steps of vein differentiation driven by temporally dynamic VEGF *activation* to specify pre-vein ECs, followed by VEGF/ERK *inhibition* to induce vein ECs (Fig. 2A). This temporally-dynamic signaling switch reconciles why VEGF/ERK signaling has been reported to both promote^18^ and inhibit^36–38^ vein formation *in vivo*: the same signal can do both, at different times.

### Comparison of differentiation methods suggests that VEGF/ERK inhibition is integral to venous identity

**Extended Data Figure 5:**
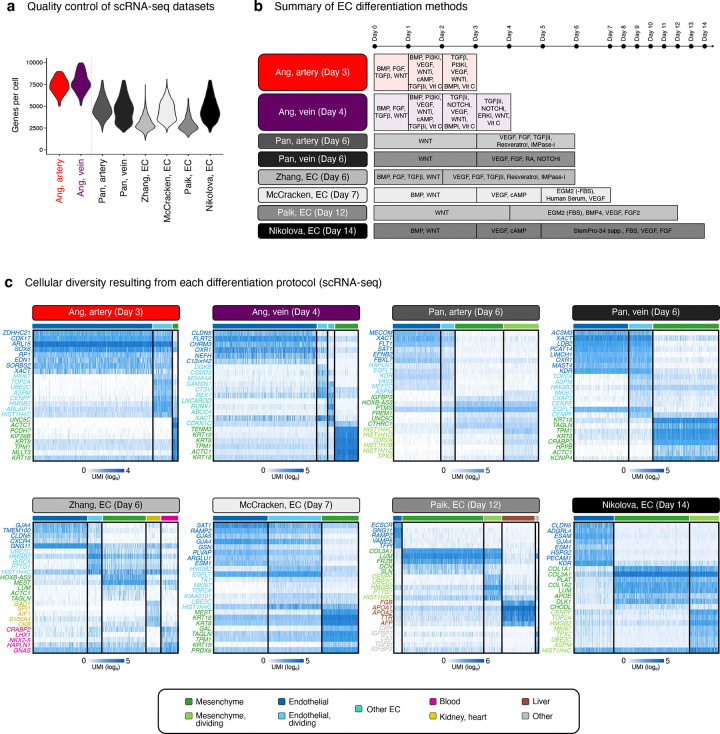
Comparison of methods to differentiate hPSCs into ECs A) scRNA-seq of hPSCs differentiated into ECs using various protocols, depicting the number of genes detected per single cell as a quality control metric. B) Summary of protocols to differentiate hPSCs into ECs^48,57–61^ used to generate the scRNA-seq datasets described in Fig. 3A. C) scRNA-seq of differentiated hPSC populations described in Fig. 3A. Subclustering was performed to assess population heterogeneity, and differentially expressed genes that distinguished cell subsets are shown. Clusters are annotated and colored as described in Fig. 3A.

**Figure 3:**
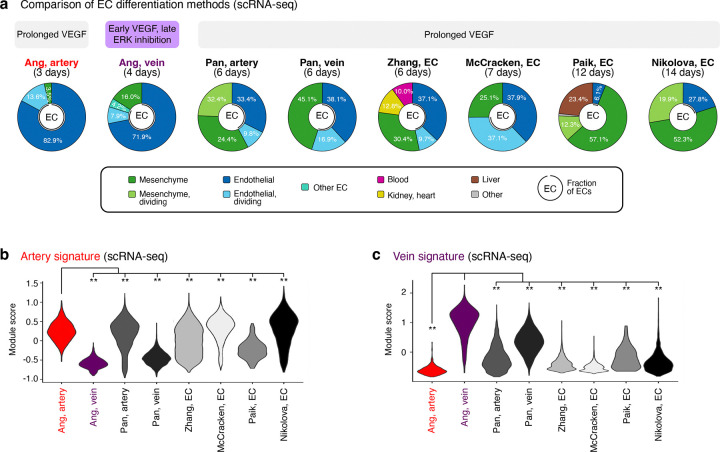
Comparison of differentiation methods suggests that temporally dynamic VEGF modulation is crucial to impart venous identity A) scRNA-seq of hPSCs differentiated into ECs using various protocols, with the number of days of differentiation indicated. scRNA-seq datasets were subclustered at the same resolution to identify population heterogeneity. Clusters were annotated by marker expression. The proportion of ECs is indicated. scRNA-seq data were obtained from this study, Ang et al., 2022^48^, Pan et al., 2024^57^, McCracken et al., 2019^61^, Paik et al., 2018^59^, and Nikolova et al., 2025^58^. B) scRNA-seq of differentiated hPSC populations described in Fig. 3A, and ECs were computationally isolated. An expression module score^121^ of arterial markers defined *in vivo* by Hou et al., 2022^14^ is shown. Statistics: Wilcoxon rank sum test. **P<0.01. C) scRNA-seq of differentiated hPSC populations described in Fig. 3A, and ECs were computationally isolated. An expression module score^121^ of venous markers defined *in vivo* by Hou et al., 2022^14^ is shown. Statistics: Wilcoxon rank sum test. **P<0.01.

Despite widespread success in differentiating hPSCs towards artery ECs, it has generally proven more challenging to generate vein ECs^3^. We hypothesized that difficulties in generating vein ECs might be attributable to the temporally-dynamic effects of VEGF: current differentiation methods typically entail prolonged VEGF activation^51–53,56–58^, which might inadvertently deter venous differentiation. To this end, we compared 8 hPSC differentiation methods by collating published scRNAseq datasets and generating new ones^48,57–61^ (Fig. 3A, Extended Data Fig. 5A–B). What is the diversity and arteriovenous character of cells emerging from each protocol?

**Extended Data Figure 6:**
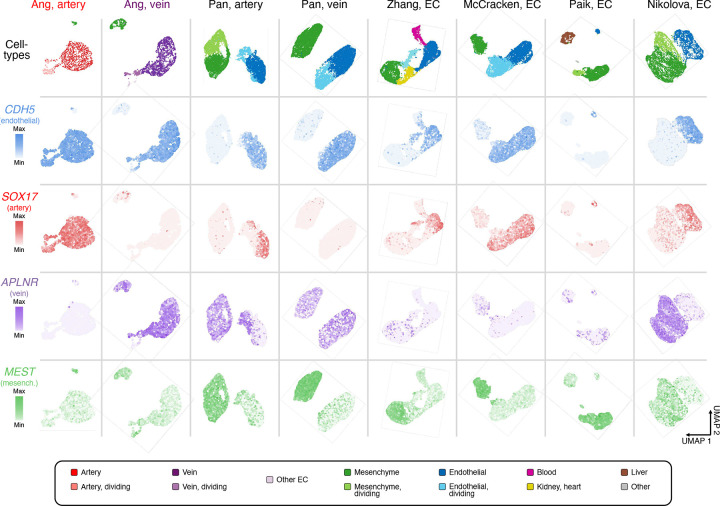
Comparison of methods to differentiate hPSCs into ECs A) scRNA-seq of differentiated hPSC populations described in Fig. 3A, depicting expression of pan-endothelial, arterial, venous, and mesenchymal marker genes. Clusters are annotated and colored as described in Fig. 3A.

**Extended Data Figure 7:**
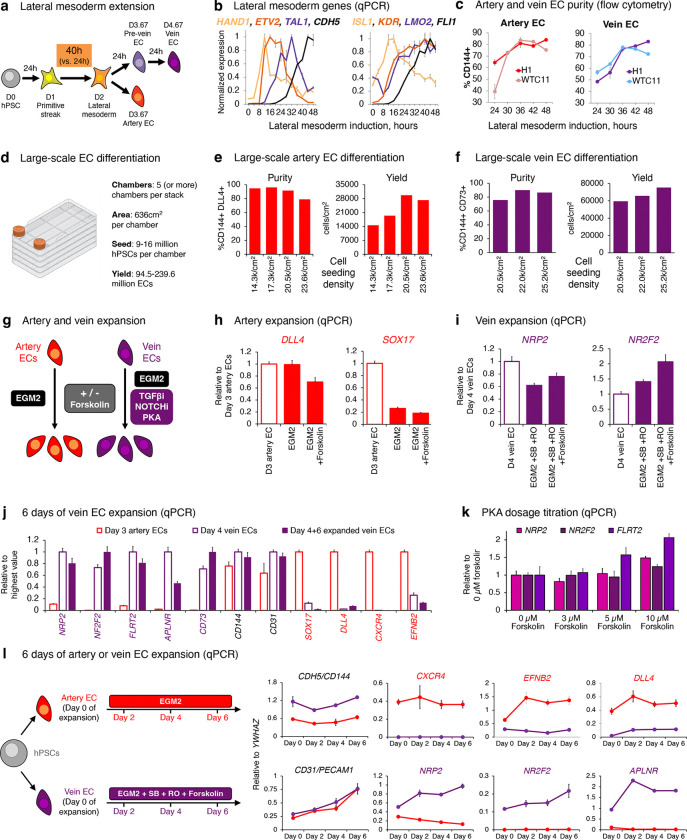
Improved generation and expansion of artery and vein ECs, while preserving arteriovenous identity A) Schematic of differentiation protocol, which includes prolongation of lateral mesoderm induction from 24 hours (V1 protocol) to 40 hours (V2 protocol). B) qPCR of lateral mesoderm and vascular marker expression in H1 hPSC-derived day-1 primitive streak cells challenged with lateral mesoderm induction media for up to 48 hours. 0 hours corresponds to day-1 primitive streak cells, prior to addition of lateral mesoderm induction media. Cells were lysed every 4 hours and qPCR was performed. For each gene, expression is normalized to the sample with the highest value, which was set to “1.0”. Error bars: SEM. C) Effect of lateral mesoderm induction duration on the subsequent production of CD144+ artery and vein ECs from the H1 and WTC11 hPSC lines, as measured by flow cytometry. Duration of lateral mesoderm induction was varied from 24 hours (V1 protocol) to 48 hours, with all other parameters of the differentiation protocol unchanged. Error bars: SEM. D) Schematic of CellSTACK system for large-scale differentiation of hPSCs into ECs. E) Yield and percentage of CD144+ DLL4+ day-3.67 artery ECs generated in the large-scale CellSTACK differentiation system, depending on initial hPSC seeding density. Yield and differentiation purity were respectively measured by flow cytometry and cell counting. F) Yield and percentage of CD73+ DLL4+ day-4.67 vein ECs generated in the large-scale CellSTACK differentiation system, depending on initial hPSC seeding density. Yield and differentiation purity were respectively measured by flow cytometry and cell counting. G) Strategy for expanding hPSC-derived artery and vein ECs. Artery ECs are cultured in EGM2 medium^48^, and vein ECs are cultured in EGM2 medium + TGFβ inhibitor + NOTCH inhibitor + PKA activator. i: inhibitor. H) qPCR of H1 hPSC-derived artery ECs that were thawed in EGM2 medium^48^ and cultured for 6 days. ROCK inhibitor (Thiazovivin, 2 μM) was added for the first day of thawing to improve cell survival, and was subsequently removed in later days. Forskolin (3 μM) was added as indicated. For each gene, expression is normalized to the sample with the highest value, which was set to “1.0”. Error bars: SEM. I) qPCR of H1 hPSC-derived vein ECs that were thawed in EGM2 medium + SB505124 (2 μM, TGFβ inhibitor) + RO4929097 (1 μM, NOTCH inhibitor)^48^ and cultured for 6 days. ROCK inhibitor (Thiazovivin, 2 μM) was added for the first day of thawing to improve cell survival, and was subsequently removed in later days. Forskolin (3 μM) was added as indicated. For each gene, expression is normalized to the sample with the highest value, which was set to “1.0”. Error bars: SEM. J) qPCR of H1 hPSC-derived vein ECs that were thawed in EGM2 medium + SB505124 (2 μM, TGFβ inhibitor) + RO4929097 (1 μM, NOTCH inhibitor) + Forskolin (10 μM, adenylate cyclase agonist) and cultured for 6 days. ROCK inhibitor (Thiazovivin, 2 μM) was added for the first day of thawing to improve cell survival, and was subsequently removed in later days. As a control, H1 hPSC-derived artery ECs prior to expansion were also analyzed. qPCR data are normalized to the sample with the highest expression, which was set to “1.0”. Error bars: SEM. K) qPCR of H1 hPSC-derived vein ECs that were thawed in EGM2 medium + SB505124 (2 μM, TGFβ inhibitor) + RO4929097 (1 μM, NOTCH inhibitor), in the presence or absence of Forskolin (3–10 μM, adenylate cyclase agonist) and cultured for 6 days. qPCR data are normalized to the sample lacking forskolin, which was set to “1.0”. Error bars: SEM. L) qPCR of pan-endothelial, arterial, and venous marker expression over the course of 6-day expansion of SUN004.2 *CAG-mScarlet* hPSC-derived artery and vein ECs. Artery ECs were expanded in EGM2 medium, whereas vein ECs were expanded in EGM2 medium + SB505124 (2 μM, TGFβ inhibitor) + RO4929097 (1 μM, NOTCH inhibitor) + Forskolin (10 μM, adenylate cyclase agonist). qPCR data normalized to reference gene *YWHAZ* (i.e., *YWHAZ* levels = 1.0). Error bars: SEM.

All 8 differentiation methods generated ECs and mesenchymal cells in varying proportions. The two differentiation protocols employed in this study^48^ yielded the highest EC percentages (84.0–96.5%), with the remaining non-ECs comprising mesenchymal cells (Fig. 3A, Extended Data Fig. 5C, Extended Data Fig. 6). Certain differentiation protocols^59,60^ yielded a wider spectrum of non-ECs, including blood, kidney, heart, and liver (Fig. 3A, Extended Data Fig. 5C, Extended Data Fig. 6). To minimize the emergence of non-ECs, we developed improved protocols to generate artery and vein ECs at higher purity and scale, yielding up to 654 million artery or vein ECs in a single batch (Extended Data Fig. 7A–F).

Next, we quantified the arteriovenous character of ECs emerging from each *in vitro* differentiation protocol, using *in vivo*-defined arteriovenous marker signatures^14^. Multiple protocols generated artery ECs—perhaps reflecting the pervasive *in vitro* use of VEGF (Extended Data Fig. 5B), which is arterializing *in vivo*^36,37^—although the arterial marker score differed between protocols (Fig. 3B, Extended Data Fig. 6). The artery EC differentiation protocol employed in this study^48^ imparted the strongest arterial identity (Fig. 3B; P<0.0001), potentially attributable to the explicit inhibition of vein-inducing signal PI3K^38,62^ to sharpen arterial identity. Only two differentiation protocols^48,57^ predominantly generated vein ECs, consistent with how it has proven more challenging to instate venous identity *in vitro*^3^. The protocol employed in this study^48^—which conferred the strongest venous identity (Fig. 3C; P<0.0001)—entailed VEGF-induced generation of pre-vein ECs, followed by VEGF/ERK inhibition to specify vein ECs. Other methods relying on sustained VEGF activation^57^ less effectively induced venous character (Fig. 3C, Extended Data Fig. 6). After generating vein ECs, we developed enhanced methods to expand them for up to 6 days *in vitro* while preserving venous identity (Extended Data Fig. 7G–L).

In summary, this comparison of differentiation protocols suggests that (1) transition through pre-vein EC intermediates and (2) temporally dynamic VEGF/ERK modulation are critical to maximize vein EC generation *in vitro*. Prolonged VEGF activation is the cornerstone of extant EC differentiation protocols (Extended Data Fig. 5B), but temporally dynamic VEGF/ERK activation, followed by inhibition, may prove decisive in imparting venous identity *in vitro*.

### Sox17+ Aplnr+ pre-vein ECs exist in early embryos and contribute to veins

**Extended Data Figure 8:**
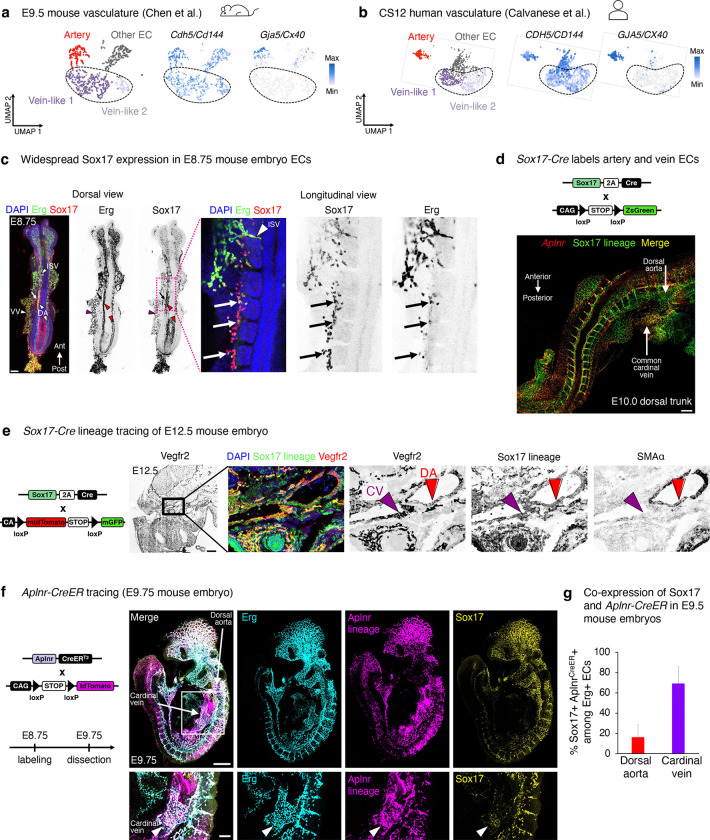
Existence and fate of *Sox17*+ *Aplnr*+ endothelial cells in the mouse embryo A) scRNAseq of E9.5 mouse embryo ECs. scRNAseq data were obtained from Chen et al., 2024^65^. B) scRNAseq of Carnegie Stage 12 (CS12) human embryo ECs. scRNAseq data were obtained from Calvanese et al., 2022^66^. C) Whole-mount Erg and Sox17 immunostaining of E8.75 mouse embryo. DA: dorsal aorta (arrowhead). VV: vitelline vein (arrowhead). ISV: intersomitic vessel (arrowhead). Arrows: solitary Sox17+ Erg+ ECs. Ant: anterior. Post: posterior. Scale: 100 μm. D) Whole-mount *Aplnr* mRNA staining of E10 *Sox17*^*Cre*^*; R26*^*zsGreen*^ mouse embryos^67,122^ by HCR3 *in situ* hybridization. Scale: 100 μm. E) E12.5 *Sox17*^*Cre*^*; R26*^*mTmG*^ mouse embryos^67,123^ were sectioned and stained for Vegfr2, SMAα, and GFP proteins. Scale: 200 μm. F) 4-hydroxytamoxifen (4OHT) was administered *in utero* to E8.75 *Aplnr-CreER*; *R26-tdTomato* mouse embryos^30,122^, which were then isolated at E9.75 and immunostained for Sox17 and Erg proteins. Bottom row arrows: cardinal vein. Scale: 200 μm (top row), 50 μm (bottom row). G) 4-hydroxytamoxifen (4OHT) was administered *in utero* to E8.75 *Aplnr-CreER*; *R26-tdTomato* mouse embryos^30,122^, which were then isolated at E9.75 and immunostained for Sox17 and Erg proteins. Quantification of Erg+ ECs in the dorsal aorta and cardinal vein that co-expressed tdTomato (indicative of *Aplnr-CreER* activity) and Sox17. Error bars: SD. **P<0.01.

**Figure 4:**
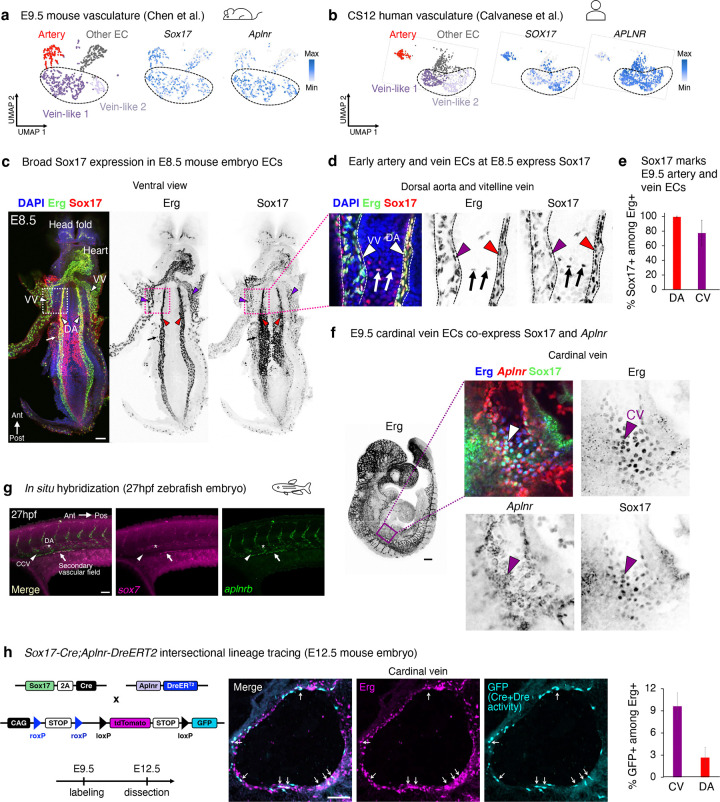
*Sox17*+ *Aplnr*+ endothelial cells exist in the early embryo, and contribute to vein endothelial cells A) scRNAseq of E9.5 mouse embryo ECs. scRNAseq data were obtained from Chen et al., 2024^65^. B) scRNAseq of Carnegie Stage 12 (CS12) human embryo ECs. scRNAseq data were obtained from Calvanese et al., 2022^66^. C) Whole-mount Erg and Sox17 immunostaining of E8.5 mouse embryo. Ant: anterior. Post: posterior. DA: dorsal aorta (arrowhead). VV: vitelline vein (arrowhead). Arrow: single migrating Erg+ Sox17+ ECs. Scale: 100 μm. D) Whole-mount Erg and Sox17 immunostaining of E8.5 mouse embryo. E) Percentage of Erg+ ECs in the E9.5 dorsal aorta (DA) or cardinal vein (CV) that express Sox17. Error bars: SD. F) Left: whole-mount Erg immunostaining of E9.5 mouse embryo. Right: E9.5 mouse embryo sectioned and immunostained for Sox17 and Erg proteins, alongside *in situ* staining for *Aplnr* mRNA. Arrow: cardinal vein. Scale: 200 μm. G) *sox7* and *aplnrb* staining of 27-hour post fertilization zebrafish embryos by HCR3 *in situ* hybridization. Lateral view of the mid-trunk region. Scale: 50 μm. H) 4OHT was administered *in utero* to E9.5 *Sox17-Cre*; *Aplnr-DreER*; *RLTG* mouse embryos^67,71,72^, which were then isolated at E12.5, cryosectioned, and immunostained for Erg and GFP proteins. Arrows: GFP+ vein ECs. Scale: 100 μm. Error bars: SD.

Having discovered *SOX17*+ *APLNR*+ pre-vein ECs *in vitro*, we investigated their existence and fate *in vivo*. Sox17 marks arteries *in vivo*^23–27^, but others suggest that it is more broadly expressed in the vasculature^63,64^. We found that *Sox17*+ *Aplnr*+ ECs exist in E9.5 mouse embryos (Fig. 4A, Extended Data Fig. 8A, Table S4) and Carnegie Stage 12 (CS12) human embryos (Fig. 4B, Extended Data Fig. 8B), through analysis of published scRNAseq data^65,66^. Whole mount immunostaining revealed that Sox17 marked most, if not all, Erg+ ECs in the E8.5 mouse embryo, including those in the dorsal aorta and the vitelline vein (Fig. 4C–D, Extended Data Fig. 8C). In E9.5 mouse embryos, Sox17 was expressed by both the dorsal aorta and cardinal vein (Fig. 4E). Indeed, permanent labeling of *Sox17*+ cells and all of their progeny with *Sox17-Cre* resulted in both artery and vein ECs being lineage traced (Extended Data Fig. 8D–E), consistent with past observations^67,68^. These lineage tracing and immunostaining results demonstrate that early vein ECs transiently express Sox17, although it is turned off later in the mature cardinal vein by E10.5^26,27^. Despite the prominent role of Sox17 in artery development^23–27^, we conclude that it is expressed by most ECs in early vascular development, and these cells construct most of the nascent vasculature.

Analogous to pre-vein ECs *in vitro*, we found that the common cardinal vein—the earliest intraembryonic vein^11^—co-expressed Sox17 and *Aplnr* in the E9.5-E10 mouse embryo. This was independently confirmed by two different means to detect *Aplnr* expression: *in situ* hybridization (Fig. 4F) and Aplnr-CreER activity (Extended Data Fig. 8F–G). While the early cardinal vein anatomically appears as a vein, from a molecular perspective it transiently expresses Sox17 and *Aplnr*; it thus mirrors pre-vein ECs *in vitro*, prior to downregulating Sox17^26,27^. Suggestive of evolutionary conservation of this pre-vein state, we found that *sox7* (a zebrafish *sox17* homolog^69^) was co-expressed alongside *aplnrb* in the zebrafish secondary vascular field (Fig. 4G), which represents future venous progenitors^70^.

Finally, we employed intersectional lineage tracing to test whether *Sox17*+ *Aplnr*+ ECs contribute to more mature vein ECs. *Sox17-Cre*^67^ and *Aplnr-DreER*^71^ driver mice were crossed with an intersectional reporter mouse^72^. In this approach, *GFP* expression denotes both Cre- and Dre-mediated recombination^72^, reflecting previous expression of *Sox17* and *Aplnr*. Tamoxifen injection at E8.5 led to GFP labeling of the E12.5 common cardinal vein, supporting the hypothesis that pre-vein ECs form vein ECs *in vivo* (Fig. 4H). However, not every vein EC was labeled, likely reflecting incomplete recombination efficiency inherent to intersectional lineage tracing, or alternative sources of vein ECs. An open question remains whether *all* vein ECs emerge via pre-vein intermediates, or whether alternative differentiation paths can also culminate in venous identity; this might be addressable in the future through unbiased CRISPR barcoding^73,74^. Taken together, *Sox17*+ *Aplnr*+ ECs contribute to veins in mouse embryos, and cells with molecular features of pre-vein ECs may be evolutionarily conserved in human and zebrafish embryos.

### SOXF transcription factors are required for both artery and vein differentiation

**Extended Data Figure 9:**
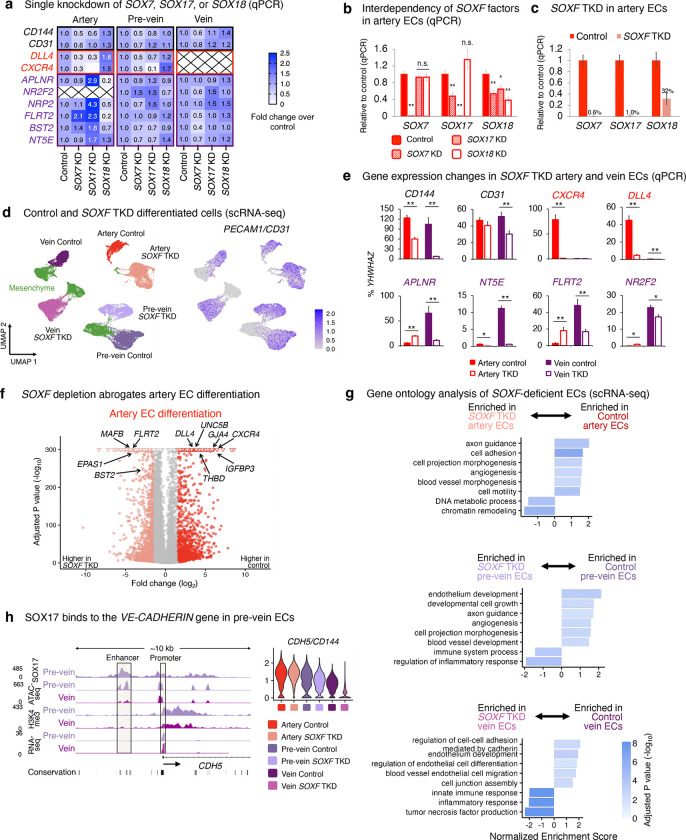
Triple CRISPRi knockdown of *SOXF* transcription factors impairs both human artery and vein EC differentiation *in vitro* A) qPCR of day-3 artery ECs derived from control and single *SOX7*-, *SOX17*-, or *SOX18*-knockdown H1 CRISPRi hPSC lines. Gene expression normalized to levels in control hPSC-derived artery ECs, which was set as “1.0”. “X” indicates that qPCR data were not shown, because gene expression was under 2% of *YWHAZ* in control samples. Statistics: unpaired t-test. **P<0.01. B) qPCR of day-3 artery ECs derived from control and single *SOX7*-, *SOX17*-, or *SOX18*-knockdown H1 CRISPRi hPSC lines. Gene expression normalized to levels in control hPSC-derived artery ECs, which was set as “1.0”. Statistics: unpaired t-test. Error bars: SEM. n.s.: not significant. *P<0.05. **P<0.01. C) qPCR of day-3 artery ECs generated from H1 control vs. *SOXF* TKD hPSCs. Gene expression normalized to control artery ECs. Percentages indicate remaining gene expression in *SOXF* TKD artery ECs, relative to controls. Statistics: unpaired t-test. Error bars: standard error of the mean (SEM). D) *Left*: scRNA-seq of day-3 artery ECs, day-3 pre-vein ECs and day-4 vein ECs generated from either H1 control or *SOXF* TKD CRISPRi hPSCs. *Right*: *PECAM1*+ ECs and *PECAM1*− mesenchymal cells were detected. The same scRNAseq datasets are shown here as in Fig. 4C, except that mesenchymal cells were computationally excluded in Fig. 4C. E) qPCR of day-3 artery ECs and day-4 vein ECs generated from H1 control vs. *SOXF* TKD CRISPRi hPSCs. Gene expression normalized to reference gene *YWHAZ* (i.e., *YWHAZ* = 100%). Statistics: unpaired t-test. Error bars: SEM. *P<0.05. **P<0.01. F) Differentially expressed genes between day-3 pre-vein ECs generated from H1 control vs. *SOXF* TKD CRISPRi hPSCs are colored. G) Gene Set Enrichment analysis (GSEA)^124^ of scRNA-seq data from H1 CRISPRi control vs. *SOXF* TKD hPSCs differentiated into day-3 artery ECs, day-3 pre-vein ECs, and day-4 vein ECs. Color represents the log_10_-transformed adjusted P-value. H) *Left*: OmniATAC-seq, CUT&RUN, and bulk RNA-seq of H1 hPSC-derived day-3 pre-vein ECs and day-4 vein ECs. *Right*: scRNA-seq of day-3 artery ECs, day-3 pre-vein ECs, and day-4 vein ECs generated from H1 control vs. *SOXF* TKD hPSCs. *SOXF* genes are required for *CDH5* (*VE-CADHERIN*) expression in pre-vein ECs.

**Figure 5:**
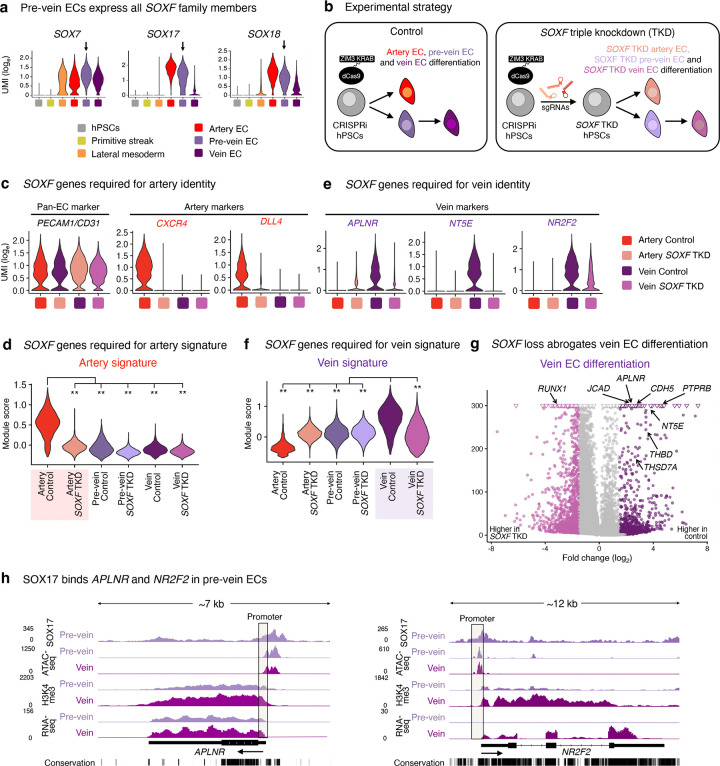
SOXF transcription factors are required for human vein EC specification *in vitro* A) scRNA-seq of day-2 lateral mesoderm, CD144+ FACS-purified day-3 artery ECs, CD144+ FACS-purified day-3 pre-vein ECs, and CD144+ FACS-purified day-4 vein ECs generated from H1 hPSCs. Arrows indicate that pre-vein ECs express *SOX7*, *SOX17*, and *SOX18*. B) H1 CRISPRi-expressing hPSCs were transduced with sgRNAs targeting *SOX7*, *SOX17*, and *SOX18* (*SOXF* triple knockdown [TKD]), and then subsequently differentiated into artery and vein ECs. C) scRNA-seq of day-3 artery ECs and day-4 vein ECs generated from H1 control vs. *SOXF* TKD hPSCs. Mesenchymal cells were computationally excluded. D) scRNA-seq of day-3 artery ECs, day-3 pre-vein ECs, and day-4 vein ECs generated from H1 control vs. *SOXF* TKD hPSCs. Mesenchymal cells were computationally excluded. A transcriptional module score^121^ computed from a panel of *in vivo*-defined arterial marker genes *in vivo*^14^ is shown. Statistics: Wilcoxon rank sum test. **P<0.01. E) scRNA-seq of day-3 artery ECs and day-4 vein ECs generated from H1 control vs. *SOXF* TKD hPSCs. Mesenchymal cells were computationally excluded. F) scRNA-seq of day-3 artery ECs, day-3 pre-vein ECs, and day-4 vein ECs generated from H1 control vs. *SOXF* TKD hPSCs. Mesenchymal cells were computationally excluded. A transcriptional module score^121^ computed from a panel of *in vivo*-defined venous marker genes *in vivo*^14^ is shown. Statistics: Wilcoxon rank sum test. **P<0.01. G) scRNA-seq of day-3 artery ECs and day-4 vein ECs generated from H1 control vs. *SOXF* TKD hPSCs. Mesenchymal cells were computationally excluded. Differentially expressed genes between control vs. *SOXF* TKD ECs are colored. H) OmniATAC-seq, CUT&RUN, and bulk RNA-seq of H1 hPSC-derived day-3 pre-vein ECs and day-4 vein ECs.

Our discovery that SOX17 marks pre-vein ECs *in vivo* and *in vitro* was surprising, as the prevailing view is that SOX17 imparts arterial identity^23–27^. Pre-vein ECs express all members of the SOXF transcription factor (TF) family: *SOX7*, *SOX17*, and *SOX18* (Fig. 5A); both SOX7 and SOX18 are also thought to confer arterial identity^28,29^. Beyond artery identity, are SOXF TFs also required for human vein EC differentiation? To answer this question, we first engineered hPSCs to express an enhanced CRISPR interference (CRISPRi) effector (dCas9-ZIM3 KRAB)^75^. hPSCs were transduced with sgRNAs targeting *SOX7*, *SOX17,* and/or *SOX18*, and then subject to EC differentiation (Fig. 5B). Individual CRISPRi knockdown of *SOX7*, *SOX17*, or *SOX18* modestly affected arteriovenous identity (Extended Data Fig. 9A–B), consistent with genetic redundancy between SOXF TFs^8,69,76^. We thus simultaneously depleted all three *SOXF* TFs using CRISPRi, and attained high knockdown efficiency (Extended Data Fig. 9C–D). As expected^23–27^, triple *SOXF* knockdown abrogated artery differentiation, with near-complete loss of arterial markers *CXCR4* and *DLL4* (Fig. 5C–D, Extended Data Fig. 9E–F, Table S5).

Interestingly, triple *SOXF* knockdown also strongly impaired vein differentiation: venous markers *NR2F2*^32^, *APLNR*^30,31^ and *NT5E/CD73*^51–53^ were reduced, as was endothelial marker *VE-CADHERIN* (Fig. 5E, Extended Data Fig. 9E,H, Table S6). Beyond individual markers, triple *SOXF* knockdown significantly impaired the venous transcriptional signature more broadly, as computed from known venous marker panels^14^ (Fig. 5F–G), and downregulated gene sets associated with blood vessel development, migration, and junction formation (Extended Data Fig. 9G).

To investigate why SOXF TFs are required for venous differentiation, we mapped the genome-wide binding of SOX17 in pre-vein ECs using CUT&RUN^54^. SOX17 directly bound the *APLNR* and *NR2F2* promoter elements in pre-vein ECs, prior to venous differentiation and predating *NR2F2* transcription (Fig. 5H, Extended Data Fig. 9H). Taken together, SOXF TFs directly bind to, and are required to activate, venous genes during human vein EC differentiation. This builds on the roles of SOXF TFs in mouse and zebrafish vein development^77–79^. We show that while SOXF is required for the specification of both artery and vein ECs, it turns on different genes in each: it activates arterial genes in artery ECs, but turns on venous genes in vein ECs.

### Accessible chromatin landscapes link VEGF/ERK inhibition to venous identity

**Extended Data Figure 10:**
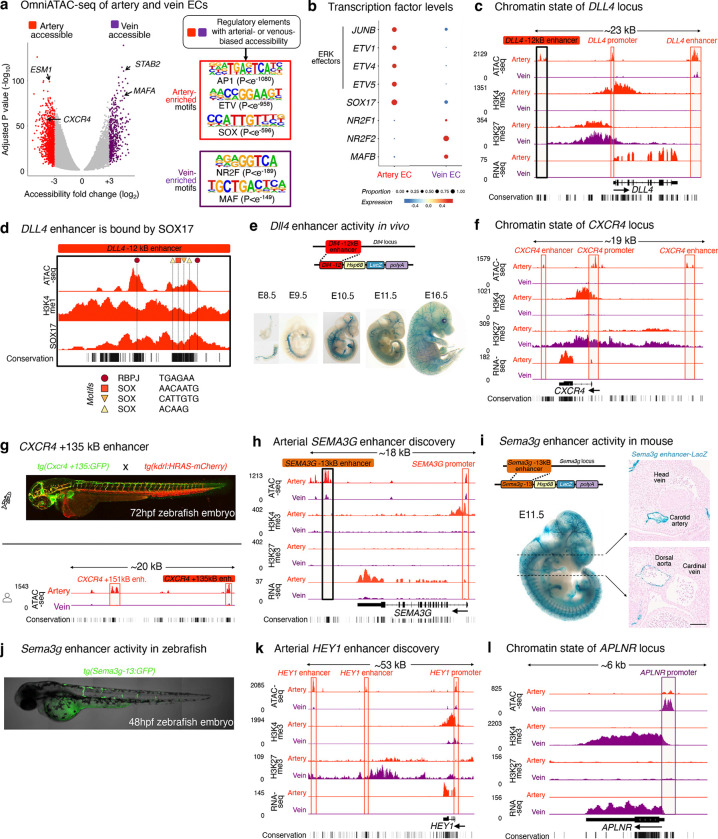
Chromatin hallmarks of human arteriovenous identity A) OmniATAC-seq of H1 hPSC-derived day-3 artery ECs and day-4 vein ECs. Left: genetic loci preferentially accessible in either artery or vein ECs are colored. Right: transcription factor motifs enriched in artery- or vein-accessible chromatin elements. B) Expression of AP1, ETV, SOX, NR2F, and MAF family transcription factors at the mRNA level, as shown by scRNAseq of FACS-purified CD144+ DLL4+ CD73lo/− day-3 artery ECs and CD144+ DLL4− CD73hi day-4 vein ECs generated from H1 hPSCs. C) OmniATAC-seq, CUT&RUN, and bulk RNA-seq of H1 hPSC-derived day-3 artery ECs and day-4 vein ECs. D) OmniATAC-seq and CUT&RUN analysis of the *DLL4* −12kb enhancer^81^ in H1 hPSC-derived day-3 artery ECs, with transcription factor motifs labeled. E) LacZ staining of E8.5-E16.5 mouse embryos bearing a *Dll4* −12 kB enhancer transgene driving *LacZ* expression^81^. F) OmniATAC-seq, CUT&RUN, and bulk RNA-seq of H1 hPSC-derived day-3 artery ECs and day-4 vein ECs. G) *Top*: image of a 3 day-post-fertilization (dpf) zebrafish bearing a *Cxcr4* +135 kB enhancer driving *GFP* expression, together with a *kdrl:HRAS-mCherry* transgene to label ECs^82^. *Bottom*: ATAC-seq of H1 hPSC-derived day-3 artery ECs and day-4 vein ECs, highlighting the *CXCR4* +135 kB enhancer whose ortholog was tested in the zebrafish transgenic assay^82^. H) OmniATAC-seq, CUT&RUN, and bulk RNA-seq of H1 hPSC-derived day-3 artery ECs and day-4 vein ECs. I) LacZ staining of E11.5 mouse embryo bearing a *Sema3g* −13 kB enhancer transgene driving *LacZ* expression (VISTA Enhancer Browser ID hs2179)^85,86^. Scale: 100 μm. J) Image of a 2 day-post-fertilization (dpf) zebrafish bearing a *Sema3g* −13 kB enhancer driving *GFP* expression. K) OmniATAC-seq, CUT&RUN, and bulk RNA-seq of H1 hPSC-derived day-3 artery ECs and day-4 vein ECs. L) OmniATAC-seq, CUT&RUN, and bulk RNA-seq of H1 hPSC-derived day-3 artery ECs and day-4 vein ECs.

We reasoned that TF motifs enriched in either artery- or vein-specific genomic regulatory elements might illuminate regulators of arteriovenous identity in an unbiased way. To this end, we mapped accessible chromatin in FACS-purified hPSC-derived artery and vein ECs using OmniATACseq^49^ (Extended Data Fig. 10A, Table S7). AP1 and ETS TFs are transcriptional effectors of ERK signaling^80^, and remarkably their motifs comprised the top 15 TF motifs enriched in artery-specific regulatory elements, and were correspondingly depleted in vein-specific regulatory elements (P<10^−1080^ and P<10^−958^, respectively; Extended Data Fig. 10A, Table S8–S9). At the mRNA level, AP1 (*JUNB*) and ETS (*ETV1*, *ETV4*, and *ETV5*) family TFs were upregulated in artery, relative to vein, ECs (Extended Data Fig. 10B). As a positive control, motifs of known arterial (e.g., SOXF) and venous (e.g., NR2F, MAF) TF families were also reciprocally enriched in artery- or vein-specific regulatory elements (Extended Data Fig. 10A–B, Table S8–S9). In summary, the accessible chromatin landscape of artery ECs is dominated by ERK transcriptional effectors, whereas vein EC accessible chromatin is depleted of such ERK effectors. The accessible chromatin landscapes of artery and vein ECs reveal how extracellular signals shape chromatin, and link VEGF/ERK inhibition to venous identity.

Chromatin profiling illuminated a wealth of regulatory elements with arterial- or venous-specific accessibility (Extended Data Fig. 10A). In addition to known arterial enhancer elements in the *DLL4* and *CXCR4* loci^81,82^ (Extended Data Fig. 10C–G), our chromatin data revealed a novel, arterial-specific enhancer 13 kB upstream of *SEMA3G* gene, which encodes an arterially expressed guidance cue required for vascular pathfinding^83,84^ (Extended Data Fig. 10H). Transgenic reporter assays delineated that this novel *Sema3g* enhancer was active in arteries, but not veins, within mouse^85,86^ and zebrafish embryos, attesting to its arterial specificity (Extended Data Fig. 10I–J). Taken together, hPSC-derived artery and vein ECs are distinguished by a wealth of arteriovenous regulatory elements at the chromatin level. Our genomic resource provides a means to identify additional such elements (Table S7, Extended Data Fig. 10K–L), as exemplified by the arterial *SEMA3G* enhancer.

### Vein ECs respond differently to hemorrhagic fever viruses than artery ECs

Finally, we leveraged developmental knowledge of the pre-vein EC state to differentiate hPSCs into vein ECs for disease modeling, and to explore whether they behave differently than artery ECs. Ebola virus (family *Filoviridae*)^87–90^ and Andes virus (family *Hantaviridae*)^91–93^ cause deadly vascular diseases, infect ECs, and can only be studied in high-containment laboratories^94^. The *individual* effects of either Ebola virus^87,95^ or Andes virus^96–99^ on ECs have been studied. However, fundamental questions in comparative virology are: (1) do different viruses exert similar effects on the same cell-type and (2) do different cell-types (e.g., artery and vein ECs) respond differently to the same virus?

**Figure 6:**
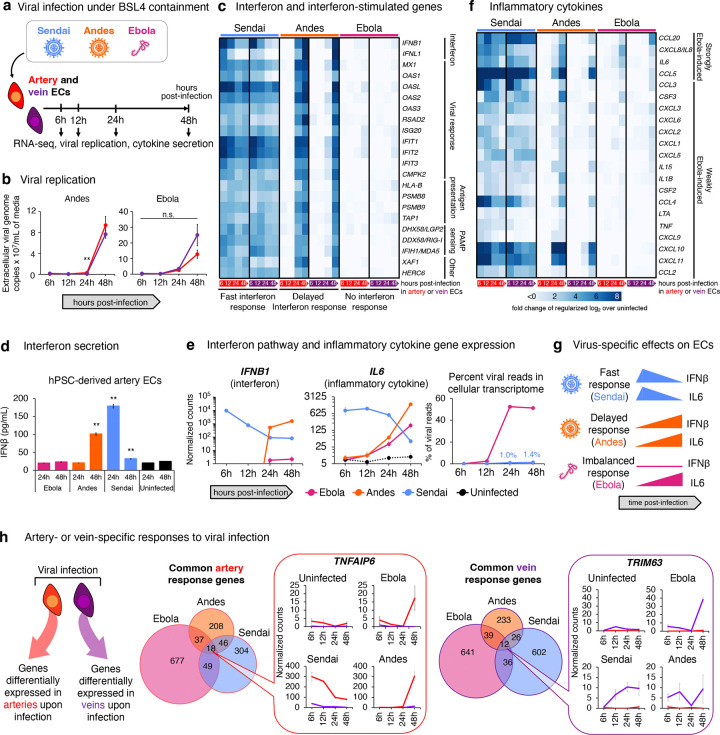
hPSC-derived artery and vein ECs respond differently to Ebola and Andes viruses A) Experimental summary. h: hour. B) Replication of Ebola and Andes viruses in hPSC-derived artery and vein ECs, as assayed by qPCR for viral genomes in the culture media. Statistics: unpaired t-test. Error bars: SEM. **P<0.01. n.s.: not significant. C) Bulk RNA-seq of interferon and interferon-stimulated gene^102^ expression in hPSC-derived artery and vein ECs 6, 12, 24, and 48 hours after infection with Sendai, Andes, or Ebola viruses. Fold change relative to uninfected cells is depicted. D) IFNβ protein secretion by hPSC-derived artery ECs after 24 or 48 hours of infection by Ebola, Andes, or Sendai viruses, or left uninfected (mock control), as measured by ELISA. Statistics: unpaired t-test with Welch correction. **P<0.01. E) Bulk-RNA-seq of hPSC-derived artery ECs 6, 12, 24, and 48 hours after infection with Ebola, Andes, or Sendai viruses, or left uninfected (mock control). Error bars: SEM. F) Bulk RNA-seq of inflammatory cytokine gene expression in hPSC-derived artery and vein ECs 6, 12, 24, and 48 hours after infection with Sendai, Andes, or Ebola viruses. Fold change relative to uninfected cells is depicted. G) Summary of the present study. H) Bulk-RNA-seq of hPSC-derived artery ECs 6, 12, 24, and 48 hours after infection with Ebola, Andes, or Sendai viruses, or left uninfected (mock control). Error bars: SEM.

Under BSL4 containment, hPSC-derived artery and vein ECs were infected with high titers of Ebola virus, Andes virus, or as a control, the non-pathogenic Sendai virus^100^, followed by RNA-seq at 6, 12, 24, and 48 hours post-infection (Fig. 6A, Table S10). Ebola and Andes viruses each replicated with similar kinetics in both artery and vein ECs (Fig. 6B). Next, we investigated the transcriptional effects of these viruses.

**Extended Data Figure 11:**
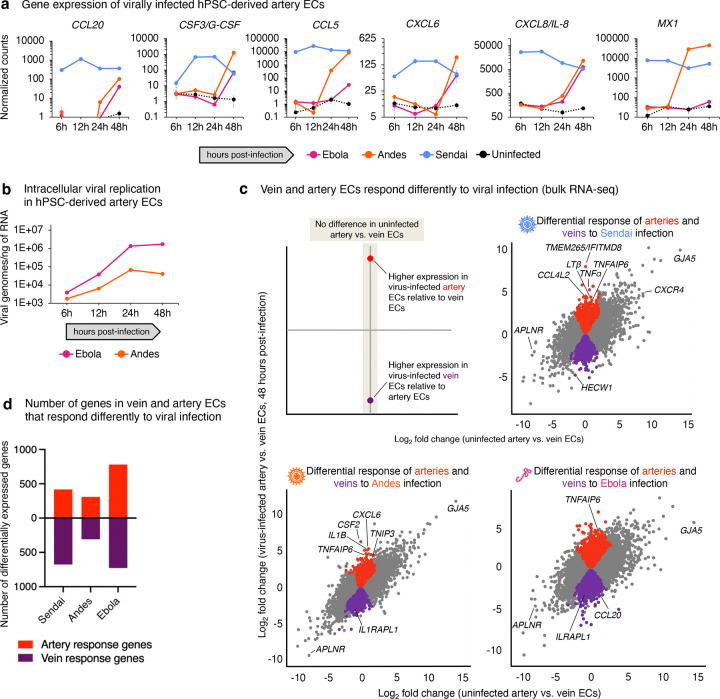
Infection of hPSC-derived artery and vein ECs by Ebola and Andes viruses A) Bulk RNA-seq of hPSC-derived artery ECs 6, 12, 24, and 48 hours after infection with Ebola, Andes, and Sendai viruses, or left uninfected (mock control). Error bars: SEM. B) Replication of Andes and Ebola viruses in hPSC-derived artery and vein ECs, as assayed by qPCR for intracellular viral genomes. C) Bulk RNA-seq of artery and vein ECs 48 hours after infection with Sendai, Andes, and Ebola viruses. Genes that are differentially expressed between infected artery and vein ECs, but are not differentially expressed between uninfected artery and vein ECs are correspondingly colored. Red: genes more highly induced by viral infection in hPSC-derived artery relative to vein ECs, and which do not show substantially different expression in uninfected artery vs. vein ECs. Purple: genes more highly induced by viral infection in hPSC-derived vein relative to artery ECs, and which do not show substantially different expression in uninfected artery vs. vein ECs. D) Number of genes that are differentially expressed in hPSC-derived artery vs. vein ECs upon infection with a given virus.

Classically, virus-infected cells secrete interferons (which activate antiviral genes that restrain viral replication) and inflammatory cytokines (which induce fever and other effects), thereby eliciting a balanced immune response^101^. Indeed, within 6 hours of infection, Sendai virus expectedly induced the expression of interferon and antiviral interferon-stimulated genes (ISGs)^102^ (Fig. 6C–E), as well as inflammatory cytokines and chemokines (Fig. 6E–F, Extended Data Fig. 11A), in both artery and vein ECs. Nevertheless, Andes virus only triggered the interferon and inflammatory programs in artery and vein ECs within 24–48 hours of infection (Fig. 6C–F, Extended Data Fig. 11A), despite substantial intracellular virus replication at 6–12 hours post-infection (Extended Data Fig. 11B). Andes virus thus temporarily delays innate immune responses in ECs, perhaps attributable to specific Andes virus proteins^103–105^. This may provide a foothold for initial viral amplification in ECs, prior to engagement of innate immunity. Andes thus mirrors other pathogenic viruses, including SARS-CoV-2, which induce interferon and inflammatory programs in delayed fashion^106^.

Ebola virus mRNAs occupied >50% of the artery EC transcriptome within 24–48 hours post-infection, but remarkably failed to induce type I interferon (*IFNβ*), type III interferon (*IFNλ1*) or antiviral ISGs to an appreciable extent (Fig. 6C,E, Extended Data Fig. 11A). The quantitative degree of interferon suppression was profound. Upon Ebola infection, there was no statistically significant increase in IFNβ secretion (Fig. 6D) nor upregulation of antiviral ISG *MX1* (Extended Data Fig. 11A) relative to uninfected ECs, whereas *MX1* was elevated 937-fold in response to Sendai infection. Correspondingly, antigen presentation ISGs (*HLA-B*, *PSMB8*, *PSMB9*, and *TAP1*^102^) were not markedly induced by Ebola, although they were upregulated in response to other viruses (Fig. 6C). By suppressing antigen presentation gene expression, Ebola may impair links between innate and adaptive immunity in ECs, consistent with defective adaptive immune responses in patients with fatal Ebola disease^107–109^.

Despite the profound lack of interferon induction, Ebola significantly induced inflammatory cytokines, including *IL6* and *CXCL8/IL8*, by 48 hours post-infection (Fig. 6E, Extended Data Fig. 11A). Indeed, IL6 and CXCL8 are elevated 100- to 1000-fold in Ebola patients, and can reach extremely high bloodborne levels (>1 ng/mL) *in vivo*^107,110^. Taken together, Ebola seemingly triggers “immunological misfiring”^111^ in human ECs by elevating inflammatory cytokines (e.g., IL6 and CXCL8), in the virtual absence of interferon and antiviral ISGs. This imbalanced innate immune response may underlie two clinical features of fatal Ebola disease: unremitting “cytokine storm”^107,110^, paired with extraordinary bloodborne viral loads^112^, the latter perhaps attributable to failed antiviral gene induction.

Finally, the same virus elicited different transcriptional responses in hPSC-derived vein vs. artery ECs (Extended Data Fig. 11C). 1509 genes were differentially expressed in vein vs. artery ECs in response to Ebola virus infection (Extended Data Fig. 11D). Such artery- or vein-specific responses to viral infection included genes involved in innate immunity and inflammation. For instance, *TRIM63* was upregulated by Ebola, Andes, and Sendai viruses in vein ECs, by comparison to artery ECs (Fig. 6H). *TRIM63* encodes an E3 ubiquitin ligase that regulates cell death^113^. Conversely, *TNFAIP6* was consistently elevated by Ebola, Andes, and Sendai viruses in artery ECs, relative to vein ECs (Fig. 6H). *TNFAIP6* encodes a stress-induced secreted protein that generally suppresses proteolysis of the extracellular matrix^114^. Vein and artery ECs may thus have distinct and hardwired responses to viral infection, illustrating that developmental cell state can impact viral infection. This exemplifies how hPSC-derived vein and artery ECs enable comparative virology studies under BSL4 containment.

## DISCUSSION

Curiously, despite substantiative insights into mechanisms of artery EC development^3–10,33,36–45^, longstanding mysteries have surrounded vein EC development^3,4^. What are the early embryonic progenitors that build veins? Why do veins emerge after arteries *in vivo*^11–17^? What are the upstream extracellular signals that specify venous identity? Here we discover pre-vein ECs that co-express the “arterial” marker *Sox17* and “venous” marker *Aplnr*, and can form vein ECs *in vivo* and *in vitro*. Cells with molecular features of pre-vein ECs also exist in human and zebrafish embryos. Collectively, the concept of pre-vein ECs unifies several observations concerning endothelial development, including why the first intraembryonic veins generally emerge after arteries in development^11–17^. We further discover that VEGF/ERK inhibition is crucial for pre-vein ECs to differentiate into vein ECs.

### VEGF/ERK activation, followed by inhibition, drives vein EC differentiation

We present evidence for a two-step model for vein development *in vitro*: VEGF/ERK *activation* is initially required to endow endothelial identity during the conversion of lateral mesoderm into pre-vein ECs, but 24 hours later, VEGF/ERK *inhibition* is subsequently required for vein EC specification. This reconciles the paradox of why VEGF/ERK both promotes^18^ and represses^36–38^ vein ECs *in vivo*: the same signal executes both roles, but at different times. The idea that VEGF/ERK inhibition is required for vein specification may be surprising, as VEGF activation is often construed as being broadly necessary for EC identity^6,18,36^. However, timing is paramount: while VEGF induces ECs, we suggest that once a cell has acquired EC identity, it now re-interprets VEGF/ERK inhibition to signify that it should adopt vein fate. Indeed, in other developmental venues, the same extracellular signal can specify different cellular identities at distinct times^115^. We propose that VEGF is a double-edged sword in venous differentiation: it induces ECs but subsequently represses venous identity. The temporally dynamic effects of VEGF likely contribute to past challenges in generating vein ECs *in vitro*. Indeed, VEGF is ubiquitous in EC differentiation and culture media, but our findings suggest that it may inadvertently suppress venous identity in certain contexts.

Our results may also pertain to the resistance of tumor vasculature against VEGF inhibitor therapies designed to block tumor angiogenesis^19,20^. If VEGF/ERK inhibition instructs ECs to adopt venous identity in certain contexts, this may represent a possible mechanism contributing to VEGF inhibitor resistance.

Why do major intraembryonic arteries precede veins *in vivo*? The answer may lie in how VEGF induces both endothelial and arterial identity^18,36–38^. Perhaps cells can contemporaneously acquire endothelial and arterial identity^11^ under the command of VEGF, but vein development must be parsed into two distinct steps wherein endothelial identity is first instated, followed by VEGF/ERK inhibition to specify veins. At the chromatin level, future venous genes—such as *NR2F2*—are in a poised state in pre-vein ECs, but are rapidly upregulated upon VEGF/ERK inhibition. This fills a vital missing link in our knowledge of the upstream extracellular signals that ignite *NR2F2* expression^3,4^. In other cell-types, ERK2 transcriptionally pauses RNA polymerase II on future developmental genes^116^. It will be interesting to test if RNA polymerase II elongation is unleashed upon VEGF/ERK inhibition in pre-vein ECs, driving *NR2F2* upregulation.

In other developmental contexts, the rich detail afforded by scRNAseq suggests that differentiation paths are continua^117,118^, piquing the philosophical question of where one cell state (e.g., pre-vein) ends and the other (e.g., vein) begins. Nevertheless, pre-vein ECs represent a clear intermediate step of venous development, as they differ from vein ECs in multiple ways. First, they are functionally induced by opposing signals: VEGF/ERK activation vs. inhibition, respectively. Second, pre-vein ECs do not express later-stage venous markers such as *NR2F2*, but harbor bivalent chromatin at future venous genes, implying developmental poising. Third, pre-vein ECs lack venous cell-surface markers CD73 and CD317.

### SOXF transcription factors are required for vein and artery EC specification

While the early cardinal vein anatomically appears as a vein, we find that it molecularly co-expresses Sox17 and *Aplnr*. The early cardinal vein may thus transiently resemble pre-vein ECs, prior to downregulating Sox17 at later stages^26,27^. Indeed, most if not all ECs temporarily express Sox17 in the mouse embryo, as shown by immunostaining and *Sox17-Cre* lineage tracing. Consistent with how the early vasculature broadly expresses Sox17 *in vivo*, simultaneous CRISPRi knockdown of all *SOXF* family members (*SOX7*, *SOX17*, and *SOX18*) compromised both human artery and vein EC differentiation *in vitro*.

Our findings support longstanding observations that SOXF transcription factors are required for arterial specification^23–29^, but additionally suggest important roles in venous identity. Further evidence supports our conclusions. First, both arteries and veins are morphologically lost in the *Sox7*−/− *Sox17*−/− *Sox18*−/− mouse retinal vasculature^79^. Second, double *sox7* and *sox18* knockdown abrogates vein development in zebrafish^77,78^. Third, SOXF motifs reside in not just arterial enhancers, but also in pan-endothelial enhancers, implying that SOXF has broader roles in endothelial identity beyond arterialization^82^. Taken together, SOXF transcription factors are expressed in pre-vein ECs, and are crucial for human vein EC differentiation.

While SOXF is necessary for the specification of artery and vein ECs, it activates different genes in each. Our CRISPRi studies showed that SOXF upregulates arterial genes in artery ECs, whereas it drives expression of certain venous genes (e.g., *NR2F2* and *APLNR*) in vein ECs. Indeed, TFs execute cell-type-specific roles^119^. Could differing SOXF levels contribute to these outcomes? Artery ECs express higher levels of SOX17 than pre-vein ECs (Fig. 1D). Perhaps higher vs. lower SOXF levels respectively bias ECs to arterial vs. venous identities, but wholesale SOXF depletion—as we achieved by CRISPRi—compromises both. SOXF TFs may also interact with differing cofactors in either artery vs. vein ECs to direct distinct transcriptional programs.

### A foundation for comparative virology

Finally, we leveraged the ability to mass-produce human artery and vein ECs to address central questions in comparative virology. To the best of our knowledge, this study represents the first side-by-side comparison of hemorrhagic fever viruses under BSL4 containment. We discovered differences in how multiple viruses affect the same cell-type (inter-virus differences), and how a given virus distinctly effects artery vs. vein ECs (intra-virus differences).

Building on past studies that separately examined Andes virus^96,99^ or Ebola virus^87,95^, we directly compared these viruses in the same experimental system; namely, hPSC-derived vein or artery ECs. Among the viruses studied here, Ebola virus was an outlier: we discovered that it *upregulated inflammatory cytokines* (e.g., IL6), while simultaneously *repressing interferon production*. Virus infection typically triggers two arms of innate immunity—interferon and inflammatory cytokine production^101^—yet, Ebola decoupled these two responses in opposite directions. Remarkably, Ebola-infected ECs failed to upregulate interferon or antiviral genes despite extraordinary viral loads (~1 in 2 mRNAs encoding Ebola virus). Substantial inflammatory cytokines, juxtaposed with defective interferon production and unchecked viral replication, may fuel an imbalanced immune response to Ebola infection. This is consistent with massive IL6 induction in Ebola patients^107,110^ (exceeding levels found in CAR-T induced “cytokine storm”^120^), coupled with prolific Ebola replication reaching 2 billion viral genomes per mL of blood^112^ (likely reflecting how Ebola powerfully suppresses interferon production *in vitro*).

Moreover, vein and artery ECs also responded differently to the same virus: *TRIM63* was consistently upregulated in virally infected vein ECs, whereas *TNFAIP6* was preferentially elevated in artery ECs by multiple viruses. This illustrates how a deeper understanding of vascular development, as exemplified by our discovery of pre-vein ECs, can be harnessed to generate human vascular cells for disease modeling and a multitude of practical applications.

## Methods

### Cell culture

All cells in this study were cultured in standard incubator conditions (20% O_2_, 5% CO_2_ and 37 °C).

### Human pluripotent stem cell lines

Wild-type H1 hESCs^125^, H1 *NR2F2-2A-GFP; SOX17-2A-mPlum* knock-in reporter hESCs, CRISPRi-expressing H1 hESCs, wild-type WTC11 hiPSCs^126^, and SUN004.2 *CAG-mScarlet* hiPSCs^48^ were used in this study.

All hPSC lines in this study, except for WTC11 and H1 CRISPRi hPSCs, were cultured in mTeSR Plus medium (STEMCELL Technologies). WTC11 hPSCs were cultured in Essential 8 medium^127^ (Thermo Fisher Scientific). H1 CRISPRi hPSCs were cultured in StemFlex medium (Thermo Fisher Scientific).

Methods to culture undifferentiated hPSCs have been described previously^128^. In brief: undifferentiated hPSCs were propagated in mTeSR Plus medium (STEMCELL Technologies) + 1% penicillin/streptomycin in monolayer cultures, on Geltrex basement membrane matrix-coated plates (described below). To maintain undifferentiated hPSCs, mTeSR Plus was changed either every day or every other day as per the manufacturer’s recommendations. In order to maintain cultures of undifferentiated hPSCs, when they became partially confluent, undifferentiated hPSCs were passaged by treating them for 7 minutes with Versene (Thermo Fisher; an EDTA-based dissociation buffer) at room temperature. Subsequently, Versene was removed, mTeSR Plus was added, and then hPSCs were manually scraped off the plate to generate clumps. hPSC clumps were then seeded onto new plates that had been precoated with Geltrex basement membrane matrix (described below) in mTeSR Plus medium + 1% penicillin/streptomycin. To reiterate, during Versene-based maintenance passaging of undifferentiated hPSCs as clumps, ROCK inhibitor was not added.

### Mouse models

Wild-type CD-1 mice (Charles River, catalog number 022), *Sox17-Cre* mice (provided by Heiko Lickert’s laboratory^67^), *Aplnr-CreER* mice (provided by Kristy Red-Horse’s laboratory^30^), *Aplnr-DreER* mice (provided by Sophie Astrof’s laboratory^71^), *ROSA26-LSL-zsGreen* mice (Ai6; JAX stock 007906, and developed by the Allen Brain Institute^122^), *ROSA26-LSL-tdTomato* mice (Ai14; JAX stock 007914, and developed by the Allen Brain Institute^122^), *RC::RLTG* mice (JAX stock 026931, and developed by Patricia Jensen’s laboratory^72^), *Sema3g* −*13kB enhancer-LacZ* mice (developed by Len Pennacchio’s and Axel Visel’s laboratories^85^), and *Dll4* −*12 kB enhancer-LacZ* mice (developed by Sarah De Val’s laboratory^81^), were used in this study.

### Zebrafish models

Wild-type AB zebrafish and *Cxcr4* +*135 enhancer-GFP*; *kdrl-HRAS-mCherry* zebrafish (developed by Sarah De Val’s laboratory^82^) were used in this study.

### Ebola virus, Yambuku variant, Mayinga isolate

Ebola virus, Yambuku variant, Mayinga isolate (Ebola virus/Human/COD/1976/Yambuku-Mayinga; genomic sequence reported in NCBI accession number AF086833.2) was originally isolated by the CDC from a fatally-infected human in the Democratic Republic of the Congo in 1976, and was passaged on Vero E6 cells^129^. Ebola virus is a member of the species *Orthoebolavirus zairense*; genus *Orthoebolavirus*; family *Filoviridae*; order *Mononegavirales*^130^. Ebola virus, Yambuku variant, Mayinga isolate was obtained from NIH Rocky Mountain Laboratories reference stocks.

### Andes virus, South variant, Chile-9717869 isolate

Andes virus, South variant, Chile-9717869 isolate (Andes virus/Oligoryzomys longicaudatus/CHL/1997/Chile-9717869; genome sequence reported in NCBI accession numbers AF291704 [L genome segment], AF291703 [M genome segment], and AF291702 [S genome segment]) was originally isolated by the CDC from an infected *O. longicaudatus* rodent in Chile in 1997, and was passaged on Vero E6 cells^93,131^. Isolate Chile-9717869 has been genomically assigned to the South variant^132^. Andes virus is a member of the species *Orthohantavirus andesense*; genus *Orthohantavirus*; family *Hantaviridae*; order *Bunyavirales*. Andes virus, South variant, Chile-9717869 isolate was obtained from NIH Rocky Mountain Laboratories reference stocks.

### Sendai virus, Cantell strain

The original provenance of Sendai virus, Cantell strain is unknown, but it was passaged over 100 times in chicken embryonated eggs at the Central Public Health Laboratory in Helsinki, Finland^133^. Sendai virus is a member of the species *Murine respirovirus*; genus *Respirovirus*; family *Paramyxoviridae*; order *Mononegavirales*. Sendai virus, Cantell strain was obtained from ATCC (VR-907).

## METHOD DETAILS

### Data processing and visualization

Standard plots were prepared with Microsoft Excel, Microsoft PowerPoint, or GraphPad Prism. Flow cytometry data were visualized with FlowJo. Microscope images were visualized with Fiji^134^. Genomics data were respectively wrangled and plotted using dplyr^135^ and ggplot2^136^ in the tidyverse^137^, in the RStudio environment. Genomics tracks were visualized with the Integrated Genomics Viewer (IGV)^138^, and evolutionary conservation was depicted using the Phastcons 20-way mammalian conservation track. Color palettes were chosen with the assistance of https://colorbrewer2.org/.

### Mouse husbandry

Adult mice of the indicated genotypes were mated to generate timed pregnancies. Females were checked each morning for a vaginal plug; noon on the day a plug was observed was defined as embryonic day 0.5 (E0.5).

### Lineage tracing

- ***Sox17-Cre;Aplnr-DreER* lineage tracing**: As described previously^139^, 25 mg of (*Z*)-4-hydroxytamoxifen (4OHT; Sigma, H7904) was dissolved in 1250 μL ethanol (Fisher Scientific, BP2818500) by vortexing and heating at 60 °C. This yielded a 20 mg/mL stock of 4OHT, which was aliquoted and stored at −20 °C. Prior to dosing mice, 50 μL aliquots (containing 1 mg of 4OHT) were heated for 10 minutes at 65 °C, and then combined with pre-warmed corn oil (250 μL, Sigma, C8267). This mixture of 4OHT and corn oil was thoroughly vortexed. Pregnant females were intraperitoneally injected at the specified labeling timepoint with 1 mg of 4OHT per mouse.
- ***Aplnr-CreER* lineage tracing**: 25 mg of (*Z*)-4-hydroxytamoxifen (4OHT; Cayman Chemical, 14854) was dissolved at 10mg/mL in ethanol (Fisher Scientific, BP2818500) by vortexing, and then was aliquoted and stored at −80 °C. Pregnant female mice were intraperitoneally injected with 28 mg 4OHT per kg of mouse weight at the specified labeling timepoint.

### *In situ* hybridization and immunostaining of whole-mount mouse embryos

Fluorescent *in situ* hybridization (FISH) of whole-mount mouse embryos was performed using hybridization chain reaction v3.0 (HCR3)^140^, and some instances, immunostaining was simultaneously performed. HCR3 was performed as per the Molecular Instruments protocol (https://www.molecularinstruments.com/hcr-v3-protocols), and is briefly summarized here.

Mouse embryos were dissected in ice-cold 4% paraformaldehyde (Fisher Scientific, 50-980-495, diluted in PBS). They were then subsequently fixed overnight in 4% paraformaldehyde, sequentially dehydrated in methanol solutions of increasing concentration (Fisher Scientific, A412-1), and then incubated in 100% methanol overnight.

Embryos were subsequently permeabilized in PBS + 0.1% Triton X-100 for 1 hour at room temperature, and then blocked in blocking buffer (PBS + 0.05% Triton X-100 + 5% BSA) overnight at 4 °C. Embryos were then stained with primary antibodies (diluted in blocking buffer) for 24–72 hours at 4 °C, washed three times with PBS, and stained with secondary antibodies (diluted in blocking buffer) for 24 hours at 4 °C. Subsequently, embryos were washed three times with PBS, and then HCR3 was performed as per the Molecular Instruments protocol. Hybridization mRNA probes, amplifiers, and buffers were obtained from Molecular Instruments. Embryos were incubated in DAPI + SSCT (sodium chloride, sodium citrate, and Tween buffer) prior to mounting. Images were captured on an Olympus FV3000 confocal microscope.

### Immunostaining of whole-mount mouse embryos

Mouse embryos were dissected in ice-cold PBS, and then fixed in 4% paraformaldehyde (Electron Microscopy Sciences, 15714; diluted in PBS) for 1 hour at 4 °C. Subsequently, embryos were washed in PBS and stained with primary antibodies (diluted in PBS + 0.1% Triton X-100) overnight at 4 °C. Following three washes in PBS + 0.1% Triton X-100, embryos were incubated in secondary antibodies (diluted in PBS + 0.1% Triton X-100) overnight at 4 °C. Embryos were then washed in PBS + 0.1% Triton X-100, counterstained with 1 μg/ml DAPI (Sigma, D9542), and washed twice in PBS. Embryos were then equilibrated sequentially in 25%, 50%, and 75% glycerol in PBS for 30 minutes at room temperature without agitation. Embryos were then mounted in Fluoromount-G (SouthernBiotech, 0100-010) and imaged on a Zeiss LSM 980 confocal microscope.

### Immunostaining of cryosectioned mouse embryos

Mouse embryos were dissected in ice-cold PBS, and then fixed in 4% paraformaldehyde (Electron Microscopy Sciences, 15714; diluted in PBS) for 1 hour at 4 °C. Subsequently, embryos were washed in PBS, and dehydrated in 30% sucrose at 4°C overnight. Embryos were embedded in OCT medium (Fisher Scientific, 23-730-571), and then frozen overnight at −80 °C. Embryos were sectioned to a thickness of 20 μm and incubated at room temperature for 30 minutes. Sections were subsequently permeabilized in PBS + 0.1% Triton X-100 for 15 minutes at room temperature and blocked in 5% donkey serum + PBS + 0.05% Triton X-100 at room temperature. Sections were then stained with primary antibodies (diluted in blocking buffer) at 4 °C overnight, washed with PBS + 0.05% Triton X-100. They were then stained with secondary antibodies (diluted in blocking buffer) at room temperature, followed by additional washes in PBS + 0.05% Triton X-100, counterstained with 1 μg/ml DAPI (Sigma, D9542) for 10 minutes at room temperature, and then washed with PBS. Slides were then mounted with Fluoromount-G (SouthernBiotech, 0100-010) and imaged on a Zeiss LSM 980 confocal microscope.

### Quantification of mouse embryo images

Fiji^134^ was used to generate maximum-intensity projections from z-stacks of whole-mount mouse embryos or sectioned mouse embryos.

- Sox17 immunostaining: Erg+ or Sox17+ cells were manually counted in the dorsal aorta (DA) and cardinal vein (CV). The percentage of Erg+ cells that expressed Sox17 is shown.
- *Sox17-Cre;Aplnr-DreER* lineage tracing: Erg+ or GFP+ cells were manually counted in the DA and CV. The percentage of Erg+ cells that expressed GFP is shown.
- *Aplnr-CreER* lineage tracing: Erg+, Sox17+, or tdTomato+ cells were manually counted in the DA and CV. The percentage of Erg+ cells that co-expressed both Sox17 and tdTomato is shown.

### Functional testing of *Dll4* enhancer element in mouse embryos

All animal procedures were approved by a local ethical review committee at Oxford University and licensed by the UK Home Office.

A stable transgenic mouse line bearing a genomic integration of the *Dll4* −12 kb enhancer element driving *LacZ* reporter expression, known as *Tg(Dll4-12:lacZ)*, was generated as previously described^81^. Embryos were fixed in 2% paraformaldehyde + 0.2% glutaraldehyde + 1x PBS for 60 minutes. After fixation, embryos were rinsed in 0.1% sodium deoxycholate, 0.2% Nonidet P-40, 2 mM MgCl_2_ and 1x PBS, then stained for 2–24 hours in 1 mg/ml 5-bromo-4-chloro-3-indolyo-β-D-galactoside solution (X-gal) containing 5 mM potassium ferrocyanide, 5 mM ferricyanide, 0.1% sodium deoxycholate, 0.2% Nonidet P-40, 2 mM MgCl_2_, and 1x PBS. After staining, embryos were rinsed through a series of 1x PBS washes, then fixed overnight in 4% paraformaldehyde at 4°C. All embryos were imaged using a Leica M165C stereo microscope equipped with a ProGres CF Scan camera and CapturePro software (Jenoptik).

### Functional testing of *Sema3g* enhancer element in mouse embryos

The activity of the *Sema3g* −13 kb enhancer element was tested in mouse embryos through a transgenic reporter assay by Len Pennacchio’s and Axel Visel’s laboratories, and was reported as part of the VISTA Enhancer Browser^85,86^ (VISTA Enhancer Browser ID hs2179: https://enhancer.lbl.gov/vista/element?vistaId=hs2179&alleleId=0&backbone=hZR&stage=e11.5). In brief, a reporter construct containing the *Sema3g* −13 kb enhancer element and the *Hsp68* minimal promoter driving *LacZ* reporter expression was randomly integrated into the mouse genome; whole-mount staining of mouse embryos for *LacZ* reporter expression was then performed^85,86^.

### Fluorescent *in situ* hybridization of zebrafish embryos

FISH of whole-mount zebrafish embryos was performed using HCR3^140^.

Wild-type AB strain embryos were fixed overnight with 4% paraformaldehyde (BT-Fix) in PBS at 4 °C, dehydrated through a sequential ethanol series, and stored at −20°C. Embryos were rehydrated and washed three times with PBT (PBS + 0.2% bovine serum albumin + 0.2% Tween 20). A prehybridization step was done using hybridization buffer (30% formamide + 5x SSC + 9 mM citric acid at pH 6.0 + 0.1% Tween 20 + 50 μg/ml heparin + 1x Denhardt’s solution, + 10% dextran sulfate) for 30 mins at 37 °C.

Following this, each 2 pmol of each HCR3 probe was combined with 500 μL of hybridization buffer and zebrafish embryos were incubated overnight in this solution at 37°C, while being gyrated. Following incubation, embryos were washed four times with 30% formamide + 5x SSC + 9 mM citric acid at pH 6.0 + 0.1% Tween 20 + 50 μg/mL heparin at 37°C, for 15 minutes each. This was then followed with three washes with 5x SSCT (5x SSC + 0.1% Tween 20) at room temperature for 5 minutes each. Subsequently, 30 minutes of incubation in amplification buffer (5× SSC + 0.1% Tween 20 + 10% dextran sulfate) at room temperature was performed. Hairpin probes (30 pmol each), fluorescently labeled through snap cooling of 3 μM stock solution, were added to the embryos in amplification buffer and incubated overnight at room temperature in the dark. Subsequently, samples were washed five times with 5x SSCT. Embryos were mounted in 0.6% low-melting agarose and imaged using a Nikon A1R confocal microscope and a 20× objective. Images were processed using the denoiseAI function in the NIS Elements software (Nikon) to reduce noise. Maximum intensity images were obtained with Fiji^140^.

### Differentiation of hPSCs into artery and vein ECs

Two differentiation protocols were used throughout this study, which we refer to “Version 1” (V1, which was used for most experiments) and “Version 2” (V2, which was only used in Extended Fig. 7A–F). The V1 differentiation protocol has been described previously^48,128^. All RNA-seq, ATAC-seq, CUT&RUN, and LEGENDScreen profiling in this study was conducted on cells generated by the V1 differentiation protocol.

The V2 differentiation protocol was developed as part of this study and is more efficient at generating both artery and vein ECs than the V1 protocol. The V2 differentiation protocol is identical to the V1 protocol, with the exception that the V1 protocol entails 24 hours of lateral mesoderm differentiation, whereas the V2 protocol entails 40 hours of lateral mesoderm differentiation.

All differentiation was conducted in defined, serum-free CDM2 basal media^141,142^. Detailed instructions to how to prepare CDM2 are available^128^.

- **Seeding hPSCs for differentiation** (**Step 0**). In preparation for differentiation, hPSCs were dissociated into single cells using Accutase (Thermo Fisher), because sparse seeding of cells is important for differentiation. Accutase-dissociated hPSCs were plated into recipient wells in mTeSR medium supplemented with the ROCK inhibitor thiazovivin (1 μM, Tocris; to enhance hPSC survival after passaging) onto plates precoated with Geltrex basement membrane matrix. hPSCs were seeded at a density of 25,000–50,000 cells/cm^2^ were seeded (i.e., 95,000–190,000 hPSCs/well of a 12-well plate)^48,128^. To clarify, long-term maintenance of undifferentiated hPSCs entailed passaging as clumps using Versene (an EDTA-based dissociation buffer; to maintain normal karyotype), but hPSCs were dissociated using Accutase to seed single cells for differentiation. 24 hours after seeding in mTeSR + 1 μM thiazovivin, during which the hPSCs re-formed small clumps, differentiation was initiated as described below.
- **Step 1: Mid primitive streak induction, 24 hours**. Day 0 hPSCs were briefly washed (DMEM/F12, Thermo Fisher) to remove all traces of mTeSR + thiazovivin. Then, they were differentiated towards mid primitive streak in CDM2 media supplemented with Activin A (30 ng/mL, R&D Systems), BMP4 (40 ng/mL, R&D Systems), CHIR99021 (6 μM, Tocris), FGF2 (20 ng/mL, Thermo Fisher), as previously described^48,128,142^. In both the V1 and V2 protocols, mid primitive streak induction was conducted for 24 hours.
- **Step 2: Lateral mesoderm induction, 24–40 hours**. Day 1 mid primitive streak cells were briefly washed (DMEM/F12) and then differentiated towards lateral mesoderm in CDM2 media supplemented with BMP4 (40 ng/mL), GDC-0941 (2.5 μM, Cellagen Technology), Forskolin (10 μM, Tocris), SB-505124 (2 μM, Tocris), VEGF (100 ng/mL, R&D Systems), XAV939 (1 μM, Tocris) and ascorbic acid-2-phosphate (AA2P; 200 μg/mL, Sigma), as previously described^48,128^. In the V1 protocol, lateral mesoderm induction was performed for 24 hours. In the V2 protocol, lateral mesoderm induction was performed for 40 hours. Subsequently, lateral mesoderm was subjected to either artery EC induction (Step 3A; below) or pre-vein EC induction (Step 3A; below).
- **Step 3A: Artery EC induction, 24 hours**. Day 2 lateral mesoderm cells (V1 protocol) or day 2.67 lateral mesoderm cells (V2 protocol) were briefly washed (DMEM/F12) and then differentiated towards artery ECs in CDM2 media supplemented with Activin A (15 ng/mL), DMH1 (250 nM, Tocris), GDC-0941 (2.5 μM), VEGF (100 ng/mL), XAV939 (1 μM) and AA2P (200 μg/mL), as previously described^48,128^. In both the V1 and V2 protocols, artery EC induction was performed for 24 hours.
- **Step 3B: Pre-vein EC induction, 24 hours**. Day 2 lateral mesoderm cells (V1 protocol) or day 2.67 lateral mesoderm cells (V2 protocol) were briefly washed (DMEM/F12) and then differentiated into pre-vein ECs in CDM2 media supplemented with SB505124 (2 μM), DMH1 (250 nM), RO4929097 (2 μM, Cellagen Technology), VEGF (100 ng/mL), XAV939 (1 μM) and AA2P (200 μg/mL), as previously described^48,128^. In both the V1 and V2 protocols, pre-vein EC induction was performed for 24 hours.
- **Step 4B: Vein EC induction, 24 hours**. Day 3 pre-vein ECs (V1 protocol) or day 3.67 pre-vein ECs (V2 protocol) were briefly washed (DMEM/F12) and then differentiated into vein ECs in CDM2 media supplemented with SB505124 (2 μM), RO4929097 (2 μM), PD0325901, (500 nM, Tocris), CHIR99021 (1 μM) and AA2P (200 mg/mL), as previously described^48,128^. In both the V1 and V2 protocols, vein EC induction was performed for 24 hours.

Detailed methods to reconstitute each differentiation-inducing small molecule and recombinant growth factor and to prepare stocks of each are available^128^. In brief, (1) all recombinant growth factors were reconstituted in PBS + 0.1% bovine albumin fraction V (both from Thermo Fisher Scientific), (2) all small molecules except for AA2P were reconstituted in DMSO (Sigma), and (3) AA2P was reconstituted in H_2_O (Thermo Fisher Scientific), as described previously^128^.

#### Large-scale differentiation of hPSCs into artery and vein ECs

Large-scale differentiation of hPSCs was performed using the V2 differentiation protocol described above.

- **Artery EC differentiation**: WTC11 hPSCs were seeded at a density of 20.5K cells/cm^2^ in a Geltrex-coated 5-stack CellSTACK device (Corning), which yielded 94.5 million cells per device.
- **Vein EC differentiation**: WTC11 hPSCs were seeded at a density of 25.2K cells/cm^2^ in a Geltrex-coated 5-stack CellSTACK device, which yielded 239.6 million cells per device. Alternatively, WTC11 hPSCs were seeded at a density of 17.2K cells/cm^2^ in 60 15-cm dishes, which yielded 654 million cells altogether.

### Cryopreservation and maintenance of hPSC-derived artery and vein ECs

After hPSC differentiation into artery ECs or vein ECs as described above, they could be maintained for at least 6 additional days *in vitro* on Geltrex-coated cell culture plates. As described previously^48^, hPSC-derived artery ECs were expanded in EGM2 (Endothelial Cell Growth Medium 2, Lonza CC-3162), which was refreshed every 24 hours. By contrast, hPSC-derived vein ECs were expanded in EGM2 + SB505124 (2 μM) + RO4929097 (2 μM) + Forskolin (10 μM), which was refreshed every 24 hours.

Alternatively, as previously described^48^, hPSC-derived day 3 artery ECs and day 4 vein ECs were dissociated, cryopreserved in freezing media (90% PBS + 10% DMSO), and stored in liquid nitrogen. hPSC-derived artery and vein ECs were then thawed in their respective media (EGM2 for artery ECs and EGM2 + SB505124 + RO4929097 + Forskolin for vein ECs) and cultured for up to 6 days as described above, with Thiazovivin (1 μM) added for the first 24 hours post-thawing to improve cell survival.

### Flow cytometry

Cultured cells were dissociated by incubation in TrypLE Express (Thermo Fisher) for 5 minutes at 37 °C. Following dissociation, the cells were diluted with 5–10 times excess volume of FACS buffer (PBS + 1 mM EDTA [Thermo Fisher] + 2% v/v FBS [Atlanta Bio] + 1% v/v Penicillin/Streptomycin [Thermo Fisher]) and centrifuged at 500g for 5 minutes to pellet them. Each cell pellet was then resuspended in FACS buffer and incubated with fluorescently-conjugated primary antibodies for 15–30 minutes in the dark at 4°C. After staining, cells were washed twice with FACS buffer and resuspended in 100 μL FACS buffer containing DAPI (1 μg/mL) for live/dead discrimination. Flow cytometry was conducted on a Beckman Coulter CytoFlex analyzer in the Stanford Stem Cell Institute FACS Core Facility. For data analysis, cells were gated based on forward and side scatter area, followed by height and width parameters for doublet discrimination. Subsequently, live cells that were negative for DAPI were gated for marker analyses and calculations of population frequency.

In this study, we defined hPSC-derived artery and vein ECs using the following cell-surface marker combinations:

- **Artery ECs**: CD144^+^ CXCR4^+^ DLL4^+^ (and, in some experiments, CD144^+^ DLL4^+^ CD73^lo/−^). The following antibody combination was used to define arterial identity: CD144 FITC (BD Biosciences, 560411 [1:50 concentration]), DLL4 APC (Biolegend, 346508 [1:5 concentration]), and CXCR4 PE-Cy7 (BD Biosciences, 560669 [1:50 concentration]).
- **Vein ECs**: CD144^+^ CD317^+^ CD73^+^ (and, in some experiments, CD144^+^ DLL4^−^ CD73^hi^). The following antibody combination was used to define venous identity: CD144 FITC (BD Biosciences, 560411 [1:50 concentration]), CD73 APC (BD Biosciences, 560847 [1:10 concentration]), and CD317 PE-Cy7 (BioLegend, 348416 [1:20 concentration]).

The CD73 protein is encoded by the *NT5E* gene. The CD317 protein is encoded by the *BST2* gene.

### Quantitative PCR

Methods for RNA extraction, reverse transcription, and qPCR have been described previously^48^. In brief, undifferentiated or differentiated hPSCs were first lysed in 350 μL of RLT Plus Buffer and RNA was extracted using the RNeasy Plus Mini Kit (Qiagen) according to the manufacturer’s protocol. Second, 300 ng of total RNA was reverse transcribed into cDNA using the High-Capacity cDNA Reverse Transcription Kit (Applied Biosystems) according to the manufacturer’s protocol. Third, qPCR was performed in 384-well format using the SensiFAST SYBR Lo-ROX Kit (Thomas Scientific) as previously described^48,142^, using gene-specific forward and reverse primers on a QuantStudio 5 qPCR machine (Thermo Fisher). Expression of all genes was normalized to the levels of the reference gene *YWHAZ*.

This qPCR procedure was only used for biosafety level 2 (BSL2) cell cultures. A separate qPCR procedure was used for biosafety level 4 (BSL4) materials, and is detailed further below.

**Table:** 

Human gene	Forward primer	Reverse primer
*YWHAZ*	GAGCTGGTTCAGAAGGCCAAAC	CCTTGCTCAGTTACAGACTTCATGCA
*HAND1*	GTGCGTCCTTTAATCCTCTTC	GTGAGAGCAAGCGGAAAAG
*ISL1*	AGATTATATCAGGTTGTACGGGATCA	ACACAGCGGAAACACTCGAT
*VEGFR2/FLK1/KDR*	TTTTTGCCCTTGTTCTGTCC	TCATTGTTCCCAGCATTTCA
*ETV2*	CCGACGGCGATACCTACTG	CGGTGGTTAGTTTTGGGGCAT
*SCL/TAL1*	CAAAGTTGTGCGGCGTATCTT	TCATTCTTGCTGAGCTTCTTGTC
*LMO2*	ATTGGGGACCGCTACTTC	GCCCAAAAAGCCTGAGATAGT
*FLI1*	ACCTCCCACACCGACCAAT	GGACTTTTGTTGAGGCCAGAA
*CDH5/VE-CADHERIN*	AACGAGCAGGGCGAGTTCACCTTC	TAGGTGACCAGCTGCTCGTGGATC
*PECAM1/CD31*	AACAGTGTTGACATGAAGAGCC	TGTAAAACAGCACGTCATCCTT
*DLL4*	GTCTCCACGCCGGTATTGG	CAGGTGAAATTGAAGGGCAGT
*CXCR4*	CACCGCATCTGGAGAACCA	GCCCATTTCCTCGGTGTAGTT
*EFNB2*	AAGGACTGGTACTATACCCACAG	TGTCTGCTTGGTCTTTATCAACC
*SOX7*	AGCCGGAGCAGACCTTCTT	GCCGGGGAGTAATAGGCAG
*SOX17*	CGCACGGAATTTGAACAGTA	GGATCAGGGACCTGTCACAC
*SOX18*	AAGCGTGGAAGGAGCTGAAC	CGCGGCCGGTACTTGTAGTT
*NR2F2*	GCCATAGTCCTGTTCACCTCA	AATCTCGTCGGCTGGTTG
*APLNR*	CTCTGGACCGTGTTTCGGAG	GGTACGTGTAGGTAGCCCACA
*FLRT2*	CGCTGCGACAGGAACTTTG	TGGAGGTAGAGTACGGTTACG
*NRP2*	GCTGGCTATATCACCTCTCCC	TCTCGATTTCAAAGTGAGGGTTG
*NT5E/CD73*	CCAGTACCAGGGCACTATCTG	TGGCTCGATCAGTCCTTCCA

### Combined immunostaining and *in situ* hybridization of cultured cells

Combined immunostaining and *in situ* hybridization of cultured cells was performed as described by Molecular Instruments (https://files.molecularinstruments.com/MI-Protocol-2%C2%BAIF-RNAFISH-GenericSolution-Rev6.pdf). First, cultured monolayer cells were fixed, permeabilized and immunostained with primary and secondary antibodies. Next, probe hybridization, amplification, and wash steps were performed using the HCR3 protocol^140^. Imaging was conducted using an FV3000 confocal microscope (Olympus).

### Bulk-population RNA-seq of uninfected cells

We performed bulk-population RNA-seq on the following cell populations that were generated from H1 hPSCs in the same biological experiment: *1*) day-0 hPSCs (entire cell population, unsorted); *2*) day-1 mid primitive streak (entire cell population, unsorted); *3*) day-2 lateral mesoderm (entire cell population, unsorted); *4*) day-3 artery ECs (FACS-purified for CD144^+^ DLL4^+^ CD73^lo/−^ cells; 10 μM forskolin was added during day-3 artery EC induction); *5*) day-3 pre-vein ECs (FACS-purified for CD144^+^ cells); and *6*) day-4 vein ECs (FACS-purified for CD144^+^ cells). Bulk-population RNA-seq datasets of day-3 artery ECs and day-4 vein ECs were previously reported by Ang et al., 2022 (NCBI PRJNA837932)^48^.

Bulk-population RNA-seq was performed and computationally analyzed as described previously^48^. Cells were lysed in Zymo RNA lysis buffer, and RNA was purified using the Zymo Quick-RNA Microprep Kit (Zymo, R1051). RNA integrity was assessed by Agilent Bioanalyzer on-chip electrophoresis. High-quality RNA samples with RNA integrity number ≥ 8 underwent poly(A) enrichment, and libraries were prepared with indexed adaptors for multiplexing. RNA-seq libraries were then sequenced on the DNBSEQ-G400 sequencer by BGI Global Genomic Services to generate 150-bp paired-end reads. To limit batch effects, all libraries were pooled prior to sequencing and distributed across multiple planes.

FastQC^143^ was used to perform quality control of raw RNA-seq reads. Adapters and low-quality bases were trimmed with Trim Galore^144^; a Phred quality threshold of ≥ 33 was used, and reads shorter than 20 nucleotides after trimming were discarded. Reads from each library—which was originally sequenced on multiple lanes—were then concatenated to yield one file per library. RNA-seq reads were pseudoaligned to human reference genome hg38, and gene-level RNA-seq counts were then quantified, using Kallisto^145^. After quantification of gene-level RNA-seq counts, two separate computational workflows in the RStudio environment were used:

- **Workflow 1** (**Generation of volcano plots**): Gene counts were filtered with edgeR^146^. Counts were then transformed to log_2_ counts per million (CPM) using voom^147^. Differentially expressed genes were determined by limma^148^, and P values were adjusted for multiple hypothesis testing using the Benjamini-Hochberg method to control the false discovery rate.
- **Workflow 2** (**Generation of gene expression matrices**): Ensembl transcript IDs were mapped to Ensembl gene IDs using EnsDb.Hsapiens.v86^149^. Transcript-level abundance estimates were summarized to gene-level counts with tximport^150^, and then imported into DESeq2^151^ for differential expression analysis.

A separate procedure was used to perform bulk-population RNA-seq of cells that underwent viral infection under BSL4 containment, which is described below.

### Single-cell RNA-sequencing of hPSC-derived cell populations

The 10x Genomics Chromium platform was used to perform single-cell RNA-seq profiling every 24 hours during the differentiation of H1 hPSCs into artery and vein ECs using the V1 differentiation protocol.

In one experiment, the Chromium Single Cell 3’ GEM, Library & Gel Bead Kit v3 was used to profile the following samples: *1*) day-0 hPSCs (entire cell population, unsorted); *2*) day-1 mid primitive streak (entire cell population, unsorted); *3*) day-2 lateral mesoderm (entire cell population, unsorted); *4*) day-3 pre-vein EC (entire cell population, unsorted); *5*) day-3 pre-vein EC (FACS-purified for CD144^+^ cells); *6*) day-4 vein EC (entire cell population, unsorted); and *7*) day-4 vein EC (FACS-purified for CD144^+^ DLL4^−^ CD73^hi^ cells). In a second experiment, the Chromium Single Cell 3’ GEM, Library & Gel Bead Kit v3.1 was used to profile the following samples: *8*) day-3 artery EC (entire cell population, unsorted); and *9*) day-3 artery EC (FACS-purified for CD144^+^ DLL4^+^ CD73^lo/−^ cells). This scRNAseq dataset of unsorted day-4 vein ECs was previously reported by Ang et al., 2022 (NCBI PRJNA837932)^48^; all other scRNAseq datasets were generated as part of this study.

The rationale to perform scRNAseq of FACS-purified ECs was to rigorously test whether cell-surface marker combinations would enable the isolation of transcriptionally-homogeneous cell populations.

As per the manufacturer, no batch effects have been detected between the Chromium v3 and v3.1 chemistries (https://kb.10xgenomics.com/hc/en-us/articles/360047373071-Does-Cell-Ranger-distinguish-between-v3-and-v3-1-chemistry).

Sequencing libraries were prepared using Chromium Single Cell 3’ GEM Gene Expression v3 or v3.1 kits as per the manufacturer’s guidelines. Sequencing libraries were diluted in Buffer EB. Libraries were prepared with 10-nucleotide indices compatible with Illumina sequencers.

### Computational analysis of single-cell RNA-sequencing data from hPSC-derived cell populations

scRNAseq libraries were sequenced across multiple lanes on an Illumina HiSeq 4000 sequencer by Novogene. The first and last 8 nucleotides of the i7 indices were unique and thus used for demultiplexing. FASTQ sequencing files were input into Cell Ranger^152^, which was used to align reads to the hg38 reference genome (version GRCh38-2024-A), followed by filtering, barcode counting, and unique molecular identifier (UMI) counting.

Subsequent analyses were performed using Seurat v3^121^ in the RStudio environment. Cell matrix files generated from Cell Ranger were imported into R using the Seurat function “Read10x_h5”. For each individual scRNAseq dataset, we performed quality control by excluding dying/dead cells that *1*) exhibited low numbers of expressed genes, *2*) displayed anomalously low or high mitochondrial counts, or *3*) did not express reference genes *ACTB* or *YWHAZ*; additionally, we also *4*) computationally excluded likely doublets by removing cells that had significantly higher counts of expressed genes. High-quality single-cell transcriptomes that passed these quality control metrics were used for subsequent analyses.

Seurat objects from all scRNAseq datasets were merged using the “Merge” function in Seurat. scRNAseq data were then normalized using the “LogNormalized” function and scaled using a linear transformation in Seurat. Marker gene expression was depicted on UMAP plots^153^ by coloring each single cell (i.e., a dot) according to the levels of marker gene expression. Dots were randomly ordered, without visually superimposing dots that were positive for a given marker gene, thereby avoiding visual stacking bias.

For scRNAseq analysis of control vs. *SOXF*-deficient CRISPRi hPSCs that were subject to EC differentiation, first we computationally identified ECs and excluded non-ECs from the scRNAseq dataset. This was performed via Louvain clustering, which clearly distinguished *PECAM1*+ ECs vs. *PECAM1*− non-EC clusters (Extended Data Fig. 9D). EC clusters were used for subsequent analyses, using the aforementioned scRNAseq analysis workflows. Gene set enrichment analysis of gene ontology terms was performed on genes that were differentially expressed between control vs. *SOXF*-CRISPRi ECs using clusterProfiler^154^. Selected gene ontology terms were shown.

Data wrangling and plotting were respectively performed using dplyr^135^ and ggplot2^136^ in the tidyverse^137^. An interactive web browser to explore scRNAseq data was constructed using ShinyCell^155^.

### Comparing different endothelial differentiation protocols using single-cell RNA-seq

We analyzed scRNAseq datasets of hPSCs that were subjected to 8 different EC differentiation protocols. The 8 differentiated cell populations analyzed were:

- **Ang et al., day 3 artery ECs**: hPSCs were differentiated into artery ECs in 3 days using the V1 differentiation protocol described by Ang et al., 2022^48^. This new scRNAseq dataset was generated as part of this study.
- **Ang et al., day 4 vein ECs**: hPSCs were differentiated into vein ECs in 4 days using the V1 differentiation protocol described by Ang et al., 2022^48^. This scRNAseq dataset was deposited by the Ang et al., 2022 study^48^ and is publicly available from NCBI PRJNA837932.
- **Pan et al., day 6 artery ECs**: hPSCs were differentiated into artery ECs in 6 days using the Pan et al., 2024 protocol^57^. This scRNAseq dataset was deposited by the Pan et al., 2024 study^57^, and is publicly available from NCBI PRJNA1114402.
- **Pan et al., day 6 vein ECs**: hPSCs were differentiated into vein ECs in 6 days using the Pan et al., 2024 protocol^57^. This scRNAseq dataset was deposited by the Pan et al., 2024 study^57^, and is publicly available from NCBI PRJNA1114402.
- **Zhang et al., day 6 ECs**: hPSCs were differentiated into ECs in 6 days using the Zhang et al., 2017 differentiation protocol^60^. This scRNAseq dataset was deposited by the McCracken et al., 2019 study^61^, and is publicly available from NCBI GSE131736.
- **McCracken et al., day 7 ECs**: hPSCs were differentiated into ECs in 7 days using the McCracken et al., 2019 protocol^61^. This scRNAseq dataset was deposited by the McCracken et al., 2019 study^61^, and is publicly available from NCBI GSE131736. Data from 3 experimental replicates were deposited to the Gene Expression Omnibus, and our preliminary analysis revealed batch effects among these three experimental replicates. To reduce batch effects, we selected replicate 3 for analysis, as it was the replicate that contained the largest number of cells. Of note, the original McCracken et al. study refers to cells being harvested on day 8 of differentiation^61^. However, because the first day of differentiation in their procedure entails cell seeding in hPSC medium^61^, to be consistent with the nomenclature used here to describe other differentiation protocols, here we refer to these cells being differentiated for 7 days.
- **Paik et al., day 12 ECs**: hPSCs were differentiated into ECs in 12 days using the Paik et al., 2018 protocol^59^. This scRNAseq dataset was deposited by the Paik et al., 2018 study^59^, and is publicly available from NCBI GSE116555.
- **Nikolova et al., day 14 ECs**: hPSCs were differentiated into vascular organoids in 14 days using the Nikolova et al., 2025 protocol^58^, which in turn was modified from the Wimmer et al., 2019 protocol^156^ to generate vascular organoids. This scRNAseq dataset was deposited by the Nikolova et al., 2025 study^58^, and is publicly available from ArrayExpress E-MTAB-14807.

scRNAseq datasets were analyzed using Seurat v3^121^. For all scRNAseq datasets, first we selected single-cell transcriptomes that passed well-established quality control metrics, by excluding dying/dead cells that *1*) exhibited low numbers of expressed genes, *2*) displayed anomalously low or high mitochondrial counts, or *3*) did not express reference genes *ACTB* or *YWHAZ*; additionally, we also *4*) computationally excluded likely doublets by removing cells that had significantly higher counts of expressed genes. For initial analyses, we assessed all single-cell transcriptomes that passed these quality control criteria; we did not pre-select a given subpopulation of cells based on marker gene expression that might bias further analyses.

Seurat objects from all scRNAseq datasets were then merged using the “Merge” function in Seurat v3^121^. To quantify the degree of cellular heterogeneity generated by each differentiation protocol, Louvain clustering was applied at the same resolution (0.1) across all datasets, thus decomposing each scRNAseq dataset into multiple constituent cell-types. To assign the identity of each “cell-type” within each dataset, we analyzed genes that were differentially expressed between each cell-type within a given dataset, which we annotated based on the expression of known marker genes:

- **Ang et al., day 3 artery ECs** (3 constituent clusters): artery EC (*EDN1*+ *MKI67*−), dividing artery EC (*EDN1*+ *MKI67*+), mesenchyme (*ACTC1*+ *TPM1*+)
- **Ang et al., day 4 vein ECs** (4 constituent clusters): vein EC *(FLRT2*+ *NEFH*+*)*, dividing vein EC (*FLRT2*+ *DGKB*+*),* other EC (*LNCAROD*+), mesenchyme (*ACTC1*+ *TPM1*+)
- **Pan et al., day 6 artery ECs** (4 constituent clusters): EC (*EGFL7*+ *ASPM*−), dividing EC (*EGFL7*+ *ASPM*+), mesenchyme (*IGFBP3*+ *TOP2A*−), dividing mesenchyme (*IGFBP3*+ *TOP2A*+)
- **Pan et al., day 6 vein ECs** (3 constituent clusters): EC (*KDR*+ *TOP2A*−), dividing EC (*KDR*+ *TOP2A*+), mesenchyme (*ACTC1*+)
- **Day 6 Zhang et al.** (5 constituent clusters): EC (*CLDN5*+ *UBE2C*−), dividing EC (*CLDN5*+ *UBE2C*+), mesenchyme (*MEST*+ *ACTC1*+), blood-like (*SPI1/PU.1*+), heart/kidney (*NKX2.5*+ *LHX1*+)
- **McCracken et al., day 7 ECs** (3 constituent clusters): EC (*PLVAP*+ *MKI67*−), dividing EC (*PLVAP*+ *MKI67*+), mesenchyme (*MEST*+ *TAGLN*+)
- **Paik et al., day 12 ECs** (5 constituent clusters): EC (*ECSCR*+), mesenchyme (*LUM*+ *CENPF*−), proliferating mesenchyme (*LUM*+ *CENPF*+), liver-like (*FGB*+ *APOA2*+ *TTR*+ *AFP*+), unknown (co-expression of epithelial marker *EPCAM* and mesenchymal marker *LUM*)
- **Nikolova et al., day 14 ECs** (3 constituent clusters): EC (*CLDN5*+), mesenchyme (*LUM*+ *TOP2A*−), dividing mesenchyme (*LUM*+ *TOP2A*+)

### Assessing arteriovenous identity of cells generated from each hPSC differentiation protocol, using single-cell RNA-seq

We quantified the arteriovenous identity of hPSC-derived ECs that were profiled by aforementioned scRNAseq studies. Each dataset comprises a mixture of ECs and non-ECs at varying proportions. First, we computationally selected the EC cluster generated by each differentiation protocol, as per the above cluster annotations. (For differentiation protocols that generated “EC” and “dividing EC” clusters, all EC clusters were combined.) Then, in these EC populations obtained from distinct differentiation protocols, we analyzed the expression of arterial vs. venous marker gene modules, which are referred to as the “artery signature” or “vein signature” in this study. The Hou et al., 2022 study^14^ previously demonstrated that the expression of these arteriovenous gene modules is evolutionarily conserved across ECs obtained from both human and mouse embryos:

- **Arterial gene module**: *GJA4*, *UNC5B*, *DLL4*, *MECOM*, *HEY1*, *EFNB2*, *EPAS1*, *CXCR4*, *IGFBP3*
- **Venous gene module**: *NR2F2*, *NRP2*, *APLNR*, *FLRT2*

We implemented the AddModuleScore function of Seurat v3^121^ to calculate the average expression of these arterial module genes and venous module genes in hPSC-derived ECs generated from each differentiation protocol.

### Single-cell RNA-seq analysis of endothelial heterogeneity in human and mouse embryos

We downloaded scRNAseq datasets of ECs isolated from the E9.5 mouse embryo (generated by Chen et al., 2024^65^; NCBI GSE216970) or the Carnegie Stage 12 human embryo (generated by Calvanese et al., 2022^66^; NCBI GSE162950, sample GSM4968831). Computational analysis was performed as described above, with the exception that Seurat v4^157^ was used.

### OmniATAC-seq library construction

We performed OmniATAC-seq profiling of hPSC-derived day 3 artery ECs (FACS-purified for CD144^+^ DLL4^+^ CD73^lo/−^ cells) and day 4 vein ECs (FACS-purified for CD144^+^ DLL4^−^ CD73^hi^ cells). A slightly modified version (https://www.med.upenn.edu/kaestnerlab/assets/user-content/documents/ATAC-seq-Protocol-(Omni)-Kaestner-Lab.pdf) of the original OmniATAC-seq protocol^49^ was employed here, and is briefly summarized below.

First, we prepared resuspension buffer (10 mM Tris-HCl, pH 7.5 + 10 mM NaCl + 3 mM MgCl_2_ + nuclease-free H_2_O), cold lysis buffer (resuspension buffer + 0.1% v/v NP-40 + 0.1% v/v Tween-20 + 0.01% v/v digitonin), and wash buffer (99.9% resuspension buffer + 0.1% v/v Tween-20).

50,000 cells from each hPSC-derived cell-type were pelleted and washed with 500 μL of cold PBS, before lysis in 100 μL cold lysis buffer for 3 minutes on ice. To the cell lysate, 1 mL of cold wash buffer was added. The mixture was centrifuged at 500g for 10 minutes at 4 °C. Then, the supernatant (cytoplasm) was discarded and the pellet (nuclei) was retained. Transposition reaction mix from the Nextera DNA library prep kit (1x Tagment DNA [TD] Buffer + 1x PBS + 0.1% v/v Tween-20 + 0.01% v/v Digitonin + Tn5 Transposase [Tagment DNA Enzyme 1] + nuclease-free H_2_O) was added to the pellet to resuspend nuclei. The transposition reaction was incubated at 37 °C for 30 minutes on a thermal mixer, with shaking at 1,000 rpm.

DNA was purified using the Qiagen MinElute Reaction Cleanup Kit, and then PCR-amplified using Illumina i5 and i7 index primers on a thermal cycler. Then, the libraries were purified using AMPure XP beads. All OmniATAC-seq libraries were multiplexed such that they could be sequenced as a pool on a single lane. After quality control was performed on pooled libraries, deep sequencing was performed on an Illumina HiSeq sequencer (~350 million reads/lane) and an Illumina NovaSeq S4 sequencer (~2500 million reads/lane). According to general guidelines, a minimum of 50 million reads are needed to identify accessible chromatin elements and 200 million reads are needed to identify enriched transcription factor motifs (https://www.illumina.com/techniques/popular-applications/epigenetics/atac-seq-chromatin-accessibility.html) by ATAC-seq. In this study, OmniATAC-seq libraries were sequenced at a depth of 292–1908 million raw reads per library.

### OmniATAC-seq computational analysis

OmniATAC-seq data were computationally processed using the standardized ENCODE ATAC-seq analysis pipeline (https://www.encodeproject.org/atac-seq/). First, reads were aligned to human reference genome hg38 using Bowtie2^158^. Then, MACS2^159^ was used to call peaks for each library. A unified peak list for each cell-type was generated by selecting only peaks that were reproducible between the two replicates. This was achieved through an irreproducible discovery rate (IDR) analysis at the threshold of 0.05 described by the ENCODE Consortium^160^. Finally, peaks that overlapped with a “black list” of artifactual regions in hg38 (https://sites.google.com/site/anshulkundaje/projects/blacklists) were removed.

Diffbind^161^ was used to identify chromatin regions that exhibited >8-fold differential accessibility between artery and vein ECs. HOMER^162^ was used to discover DNA motifs overrepresented in these artery- or vein-accessible elements. HOMER analyses were run on repeat-masked hg38 sequences extracted in 200-nucleotide windows centered on peak summits, using the “findMotifsGenome.pl” function.

### CUT&RUN library construction

CUT&RUN profiling of H3K4me1, H3K4me3, H3K27me3, and SOX17 was conducted on H1 hPSCs differentiated into day 3 artery ECs, day 3 pre-vein ECs, and day 4 vein ECs. For most CUT&RUN experiments, CD144+ ECs were purified by FACS to exclude any contaminating mesenchymal cells, with the exception for SOX17 CUT&RUN, as SOX17 is not expressed in mesenchymal cells. As a negative control, an isotype IgG control antibody was also separately included.

CUT&RUN profiling and library construction was performed as previously described^54^. CUT&RUN libraries were sequenced on an Illumina NovaSeq X Plus by Novogene.

### CUT&RUN computational analysis

FastQC^143^ and FastQ Screen^163^ were used to perform quality control of raw CUT&RUN reads. Paired-end reads were merged using NGmerge^164^ and aligned to human reference genome hg38 with Bowtie2^64^, using the --very-sensitive mode and fragment length parameters -I 10 -X 2000. Resulting SAM files were processed with SAMBLASTER^165^ to remove PCR duplicates. SAMtools^166^ was then used to convert files into the sorted BAM format. Bigwig signal tracks were generated using the “bamCoverage” function from deepTools^167^, with 5-nucleotide bin size and values displayed in reads per kilobase million (RPKM).

### High-throughput surface marker screen by flow cytometry

The expression of 332 cell-surface markers was assessed across undifferentiated hPSCs (day 0), primitive streak (day 1), lateral mesoderm (day 2), artery ECs (day 3) and vein ECs (day 4) through the use of high-throughput flow cytometry as described previously^142^. In brief, hPSCs or their differentiated mesoderm progeny were dissociated using TrypLE Express. They were then plated into individual wells of four 96-well LEGENDScreen PE-Conjugated Human Antibody Plates (Biolegend, 700001). Each well containing a distinct antibody against a human cell-surface antigen, altogether totaling 332 unique cell-surface markers across four 96-well plates. High-throughput cell-surface marker staining was largely done as per the manufacturer’s recommendations, and cells were stained with a viability dye (DAPI) prior to robotically-enabled plate-based analysis on an BD FACSCanto II (Stanford Stem Cell Institute FACS Core). Stained cells were not fixed prior to FACS analysis. LEGENDScreen data for undifferentiated H7 hPSCs (day 0) and H7-derived anterior primitive streak (day 1) were published previously^142^. LEGENDScreen data for H1-derived lateral mesoderm (day 2), H1-derived artery ECs (day 3) and H1-derived vein ECs (day 4) was generated in this study. Day 3 artery ECs and day 4 vein ECs were both co-stained with an anti-CD144 Alexa Fluor 647 antibody (BD Biosciences, 561567) to identify CD144+ ECs, and surface-marker expression was evaluated specifically in the CD144+ population.

### Assembling CRISPRi constructs

sgRNAs targeting the human *SOX7*, *SOX17*, or *SOX18* genes were selected from genome-wide libraries of CRISPRi sgRNAs^168^.

- *SOX7* CRISPRi sgRNA 1: TCGCCTCGCTTCGCCTGGCG
- *SOX7* CRISPRi sgRNA 2: GAAGCGAGGCGACCCGCGTG
- *SOX17* CRISPRi sgRNA: GCGACAGGCCAGAACACGGG
- *SOX18* CRISPRi sgRNA: GCGGATGGCGGTGGGGACGG

To prepare sgRNA inserts, we synthesized the following oligonucleotides in preparation for introduction into the single sgRNA plasmid (harboring BstXI and Bmtl restriction sites):

- 5’ ttg + top strand sgRNA + gtttaagagc 3’
- 5’ ttagctcttaaac + bottom strand sgRNA + caacaag 3’

To prepare sgRNA inserts, we synthesized the following oligonucleotides in preparation for introduction into the dual sgRNA plasmid (harboring BstXI, BsmBI, and BlpI restriction sites):

- 5’ ttg + position A top strand sgRNA + tctca 3’
- 5’ ctcttgaga + position A bottom strand sgRNA + caacaag 3’
- 5’ gaaaggag + position B top strand sgRNA + gtttaagagc 3’
- 5’ ttagctcttaaac + position B top strand sgRNA + ctcc 3’

These oligonucleotides were annealed using 20 μL of each oligonucleotide at 100 μM concentration + 10 μL of 10x annealing buffer (100 μM Tris HCl (pH 7.5), 500 mM NaCl, 10 mM EDTA (pH 8.0)) + 50 μL water. Annealing was performed at 99 °C for 5 minutes and then brought to 25 °C for 5 minutes in a thermal cycler.

To individually knock down each of these genes, we cloned each individual sgRNA into a separate *mU6-sgRNA; EF1A-HygroR* plasmid using the Quick Ligation Kit (New England Biolabs, M2200), generally using the manufacturer’s protocol but with modified reaction volumes: 0.33 μL digested plasmid at 25 ng/μL concentration, 1.67 μL 2x quick ligase buffer, 0.166 μL quick ligase, and 1.33 μL annealed sgRNA at 20 nM concentration per reaction.

In experiments where we sought to simultaneously knockdown all three genes, we assembled a dual sgRNA plasmid using a previously-described strategy^169^. A dual sgRNA plasmid was assembled from two backbone fragments (Addgene, 187243 and 187239, respectively). We assembled a *mU6-SOX7-sgRNA 1; hU6-SOX17-sgRNA; EF1A-PuroR-2A-*GFP plasmid using the Quick Ligation Kit (New England Biolabs, M2200). The manufacturer’s protocol was generally followed, but modified reaction volumes were used: 0.33 μL of backbone plasmid 1 at 25–33 ng/μL concentration, 0.33 μL of backbone plasmid 2 at 25–33 ng/μL concentration, 1.67 μL 2x quick ligase buffer, 0.166 μl quick ligase, 0.5 μL of annealed sgRNA 1 at 200 nM concentration per reaction, and 0.5 μL of annealed sgRNA 2 at 200 nM concentration per reaction. As described below, to achieve triple *SOXF* knockdown, we transduced CRISPRi hPSCs with a *mU6-SOX18-sgRNA*; *EF1A-HygroR* plasmid, and subsequently transduced them with this dual *SOX7/SOX17* sgRNA construct.

All plasmids were transformed into Mix & Go! *E. coli* Competent Cells (Zymo Research) and purified using the Wizard Plus SV Minipreps DNA Purification System (Promega).

### CRISPRi knockdown in hPSCs

CRISPRi plasmids were packaged into VSV-G pseudotyped lentiviruses in HEK293T/17 cells using a 3^rd^ generation lentiviral packaging system as described previously^48^. 24 hours prior to transfection, HEK293T/17 cells were seeded at a density of 105,000 cells/cm^2^ in 6-well plates coated with 0.01% poly-L-Lysine. Each well was transfected with 1.39 μg pMDL plasmid + 0.78 μg VSV-G plasmid + 0.53 μg pREV plasmid + 11.3 μL FuGENE HD transfection reagent + 2.1 μg sgRNA plasmid in Opti-MEM medium. 18 hours post-transfection, media was changed to DMEM with 10% FBS. Supernatant was collected 42 hours post-transfection, filtered with 0.45 μM polyethersulfone filter, and stored at −80 °C until used for transduction.

In parallel, H1 hPSCs were engineered to constitutively express CRISPR interference (CRISPRi) machinery, namely nuclease-dead Cas9 (dCas9) fused to the transcriptional repressor ZIM3 KRAB^75^. Validation of these CRISPRi-expressing H1 hPSCs will be described in a forthcoming manuscript.

To transduce them with lentiviruses carrying single sgRNAs, H1 CRISPRi hPSCs were dissociated with Accutase, and then re-seeded at 150,000 cells/well in 6-well plates in StemFlex (Thermo Fisher, A3349401) supplemented with 1% penicillin/streptomycin and 1 μM thiazovivin and lentivirus-containing supernatant (15 μL for *SOX17*-sgRNA, 10 μL for *SOX7-*sgRNA, and 50 μL for *SOX18*-sgRNA lentiviruses, respectively). After reaching 80% confluency after 2–3 days, cells were re-plated using Accutase and cultured in StemFlex with Hygromycin B (Thermo Fisher, 10687010) at 50–100 μg/ml concentration for at least 3 weeks. 1 μM thiazovivin was supplemented for the first day of selection.

To generate the triple *SOXF* knockdown line, first we transduced H1 CRISPRi hPSCs with the *mU6-SOX18-sgRNA*; *EF1A-HygroR* lentivirus and performed hygromycin selection for at least 3 weeks, as described above. These H1 CRISPRi *SOX18-*sgRNA hPSCs were then dissociated with Accutase, re-seeded at 200,000 cells/well in 12-well plates in StemFlex supplemented with 1% penicillin/streptomycin, 50 μg/ml Hygromycin B and 1 μM thiazovivin and transduced with 50 μl of lentivirus-containing supernatant. The following day, media was replaced with StemFlex supplemented with 1% penicillin/streptomycin and 50 μg/ml Hygromycin. After 3 days, cells were re-plated using Accutase and selected with 1 μg/ml Puromycin (Thermo Fisher, A1113803) and 50 μg/mL Hygromycin B for at least 3 days. 1 μM thiazovivin was supplemented for the first day of antibiotic selection.

H1 CRISPRi hPSC lines were then further differentiated into artery and vein ECs using the V1 protocol described above. qPCR and scRNAseq was performed on control and *SOXF* knockdown artery ECs, pre-vein ECs, and vein ECs as described above.

### Construction of *SOX17-2A-mPlum; NR2F2-2A-GFP* reporter hPSC line

The Cas9/AAV6 knock-in strategy^170^ was used to generate a double *SOX17–2A-mPlum*; *NR2F2-2A-GFP* knock-in reporter hPSC line. We previously generated single *SOX17-2A-mPlum* or *NR2F2-2A-GFP* reporter hPSC lines^48^. In this study, starting from a *NR2F2-2A-GFP* reporter line (clone 10), we used our previously-described approach to knock-in a *2A-mPlum* cassette downstream of the endogenous *SOX17* gene such that the *SOX17* stop codon was removed^48^.

### Generation of Andes and Ebola virus stocks

All experiments with Risk Group 3 and Risk Group 4 viruses were conducted under maximum containment conditions in the biosafety-level-4 (BSL4) laboratory of the Robert Koch Institute, in accordance with approved standard operating procedures. Work at the Robert Koch Institute was performed using only terminally-differentiated artery and vein ECs.

Andes virus (Andes virus/Oligoryzomys longicaudatus/CHL/1997/Chile-9717869)^93,131^ and Ebola virus (Ebola virus/Human/COD/1976/Yambuku-Mayinga)^129^ were obtained from NIH and CDC reference stocks, as described above. These viruses were then subsequently propagated on Vero E6 cells (Vero (Vero 76, clone E6, Vero E6), from the European Collection of Authenticated Cell Cultures (ECACC)) in DMEM + 2% FBS to generate archive stocks, which were frozen at −80 °C. Archive stocks were thawed and passaged once more on Vero E6 to generate virus working stocks for experiments, which were stored at −80 °C.

In certain experiments, Sendai virus, Cantell strain^133^—a paramyxovirus not known to cause disease^100,171^—was used as a control. Sendai virus is classified in Risk Group 2, but was handled in the BSL4 laboratory for consistency. Sendai virus, Cantell strain was obtained from ATCC (VR-907).

### Quantification of Andes and Ebola virus stocks

To determine the infectious units of Ebola and Andes virus stocks by focus assay, viral titration was performed using the fluorescent focus-forming unit (FFU) assay. In brief, Vero E6 cells were seeded into 96-well tissue culture plates (for Ebola virus titration) or 24-well plates (for Andes virus titration). Cells were seeded in DMEM containing 10% FBS, 2 mM glutamine and 1% penicillin/streptomycin, at a density such that after overnight incubation, the cells were 95–100% confluent. Separately, virus stocks were diluted in DMEM in serial 10-fold dilutions. Then, the Vero E6 culture medium was aspirated and 100 μL of diluted Ebola virus or 200 μL of diluted Andes virus was added to triplicate wells of Vero E6 cells. After 1 hour of incubation, viral supernatants were removed and medium comprising DMEM + 2% FBS + 1% carboxymethylcellulose (CMC) was added. After 3 days for Ebola virus and 5 days for Andes virus, cells were stained with virus-specific antibodies. For this, the CMC-containing medium was removed, and cells were fixed in 10% formalin for 10–30 minutes, permeabilized in DPBS + 0.1% Triton X-100 at room temperature for 5 minutes, washed once in DPBS, and then incubated in blocking buffer containing 2% BSA for 1 hour at 37 °C.

Subsequently, 100 μL of primary antibody used to stain the cells for 1 hour at 37 °C. For Ebola virus, anti-Ebola NP antibody (clone 173/303/109, diluted 1:2500) was added. For Andes virus, anti-TULV1 N antibody (diluted 1:1000)—which is known to cross-react against Andes N protein^172^—was added. After primary antibody staining, cells were then washed and then incubated with 100 μL of secondary antibody (goat anti-mouse IgG Alexa 488, Jackson ImmunoResearch, 115-545-003, diluted 1:500–1:1000) for 30 minutes at 37 °C, washed once, and then foci of infected cells were enumerated. The titer/mL of the input viral supernatant was calculated using the Spearman-Karber method.

### Viral infection of hPSC-derived artery and vein ECs

As described above, cryopreserved H1 hPSC-derived artery and vein ECs were thawed in Geltrex-coated 48-well plates. They were cultured for 4 days in their respective growth media (EGM2 for artery ECs and EGM2 + SB505124 + RO4929097 + Forskolin for vein ECs) until they were confluent, with Thiazovivin (1 μM) added for the first 24 hours post-thaw to enhance survival. hPSC-derived artery and vein ECs were then inoculated with the following viruses, or left uninfected (negative control), for 1 hour:

- Andes virus: 2.2×10^4^ FFU units/well
- Ebola virus: 4.3×10^4^ FFU units/well
- Sendai virus: 8 HA units/well

After 1 hour of viral infection, cells were washed twice in EGM2 medium, and then artery and vein ECs were cultured in their respective growth media (EGM2 for artery ECs and EGM2 + SB505124 + RO4929097 + Forskolin for vein ECs) in standard incubator conditions (37°C and 5% CO_2_). After viral infection, ECs were profiled 6, 12, 24, or 48 hours later to quantify viral replication, gene expression (by RNA-seq), and cytokine secretion (by ELISA), as described below.

### Quantification of viral replication in hPSC-derived ECs

To quantify viral genomes present in the media at 6, 12, 24, and 48 hours post-infection of hPSC-derived artery and vein ECs, 140 μL of the culture media (i.e., supernatant) was placed in 560 μL of lysis buffer AVL (Qiagen QIAamp Viral RNA extraction kit, Qiagen, 52904), in addition to 560 μL of 100% ethanol, in order to inactivate it for transfer out of the BSL4 laboratory. Then, RNA was extracted following the manufacturer’s instructions. Subsequently, qRT-PCR was performed using primers directed against the viral genome and the AgPath-ID One-Step RT-PCR Kit (Thermo Fisher, 4387391).

To quantify intracellular viral genomes present within the cells at 6, 12, 24, and 48 hours post-infection of hPSC-derived artery and vein ECs, the cells within each well were lysed with 350 μL RLT buffer (Qiagen RNeasy Mini Kit, Qiagen, 74106) containing 1% β-Mercaptoethanol, in addition to 350 μL of 70% ethanol, in order to inactivate materials for transfer out of the BSL4 laboratory. Afterwards, RNA was extracted following the manufacturer’s instructions. Subsequently, qRT-PCR was performed using primers and FAM-labeled probes directed against the viral genome and the AgPath-ID One-Step RT-PCR Kit (Thermo Fisher, 4387391). Following PCR conditions were used: reverse transcription was performed at 45°C for 15 min, followed by initial cDNA denaturation at 95°C for 10 minutes, followed by 43 cycles with denaturation at 95°C for 15 seconds, and annealing at 60°C for 1 minute.

**Table:** 

**Andes virus**	
Forward primer	AAGGCAGTGGAGGTGGAC
Reverse primer	CCCTGTTGGATCAACTGGTT
Probe	ACGGGCAGCTGTGTCTACATTGGA 6Fam-BBQ
**Ebola virus**	
Forward primer	GTTCGTCTCCATCCTCTTGCA
Reverse primer	TGAGGGAAAAGACCATGCTCA
Probe	TGCTCCTTTCGCCCGACTTTTGAACC 6Fam-BBQ

### Quantification of cytokine secretion by virally-infected hPSC-derived ECs

At 24 or 48 hours post-infection of hPSC-derived artery and vein ECs, supernatants (culture media) were harvested, and stored at −80 °C. Media was then thawed, and the concentration of secreted interferon-β (IFNβ) protein in the 2:1 diluted media was quantified using the Human IFNβ Quantikine QuicKit ELISA Kit (R&D Systems, QK410), as described previously^48^ and as per the manufacturer’s instructions. Absorbance was quantified using a TECAN Sunrise plate reader (Tecan Trading AG, Switzerland) with a spectral filter. The concentration of IFNβ in the samples was calculated by comparing the absorbance to that of a standard curve generated with human IFNβ, after subtracting background absorbance, using TECAN Magellan software. Data shown represent the average of results from three technical replicates using H1 hPSCs.

### RNA-seq of virally-infected hPSC-derived ECs

At 6, 12, 24, or 48 hours post-infection of hPSC-derived artery and vein ECs, cells were lysed in 350 μL of buffer RLT from the RNeasy Micro Kit (Qiagen, 74004) and then triturated with 350 μL of 70% ethanol to inactivate infectious material for transfer out of the BSL4 laboratory. RNA was then extracted as per the manufacturer’s instructions. Bulk-population RNA-seq libraries were constructed and sequenced by Novogene.

### Computational analysis of RNA-seq data from virally-infected hPSC-derived ECs

Quality control of RNA-seq reads was performed using FastQC^143^ and Trim Galore^144^, as described in the above section “Bulk-population RNA-seq of uninfected cells”. RNA-seq reads from virus-infected cells contained both human and viral reads, and two parallel workflows were used to analyze human or viral gene expression:

- **Human gene expression analysis**: Human gene expression was analyzed as described in the above section “Bulk-population RNA-seq of uninfected cells”. In brief, read pseudoalignment to human reference genome hg38 and quantification of transcript-level abundances was performed using Kallisto^145^. Ensembl transcript-to-gene ID mapping (EnsDb.Hsapiens.v86^149^), gene-level count summarization (tximport^150^), and differential gene expression analysis (DESeq2^151^) was then performed.
- **Viral gene expression analysis**: To determine the overall percentage of viral reads within a given RNA-seq library, Kallisto^145^ was used to pseudoalign reads to the reference genomes of Ebola virus, Yambuku variant, Mayinga isolate (NCBI accession number NC_002549.1) or Sendai virus (NCBI accession number NC_001552). This enabled the detection of all viral reads within a given sample. Then the number of total viral counts was divided over the number of total human + viral counts to estimate what proportion of the cellular transcriptome comprised viral transcripts. Viral read alignment to the Andes virus genome was not performed. We performed RNA-seq of polyadenylated mRNAs and therefore did not expect to capture Andes virus mRNAs, which are not polyadenylated.

Further RNA-seq analysis of human gene expression was conducted as follows:

- **Heatmaps**: The rlog function of DESeq2^151^ was used to perform regularized log transformation. Transformed counts were used to plot heatmaps of gene expression using pheatmap^173^.
- **Visualization of timecourse gene expression**: Expression counts normalized by the DESeq function of DESeq2^151^ were used to create line graphs of timecourse gene expression.
- **Differential gene expression analysis**: Genes that were differentially expressed between samples were determined using the lmFit linear modeling tool provided by limma^148^.
- **Artery- vs. vein-specific transcriptional responses to viral infection**: Genes with (1) log_2_ > 0 expression and (2) adjusted P<0.05 in virus-infected artery ECs relative to virus-infected vein ECs, and (3) no statistically significant (adjusted P>0.05) expression between uninfected artery vs. vein ECs were categorized as genes with artery-specific induction upon viral infection. The reciprocal was used to define genes with vein-specific induction upon viral infection.
- **Conserved artery- vs. vein-specific transcriptional responses to viral infection**: Finally, we determined genes that were consistently induced by viral infection in artery ECs, but not vein ECs, and *vice versa*. From the above list of genes with artery-specific induction upon viral infection (described in the above section, “Artery- vs. vein-specific transcriptional responses to viral infection”), we selected genes with greater than log_2_ 1.5-fold (i.e., 2^1.5^-fold) higher expression in infected vs. uninfected cells at 2 days post-infection. We then determined overlapping genes, e.g., genes that were consistently upregulated in artery ECs by Ebola, Sendai, and Andes infection. The same analysis was performed on vein ECs, to discover genes that were consistently upregulated in vein ECs by Ebola, Sendai, and Andes infection. These genes were enumerated in Venn diagrams.

### Quantification and statistical analyses

Statistical tests are specified in the figure legend accompanying each experimental result. Unpaired t-tests were used to test for statistical significance in qPCR and ELISA data, and P values were reported. Wilcoxon rank sum tests were performed to test for statistical significance in module score differences quantified by scRNAseq, and P values were reported. The following convention was used in figures: not significant (n.s., P>0.05), *P<0.05, and **P<0.01.

Q-values were reported for differentially-expressed genes detected by bulk-population RNA-seq, differentially-accessible genomic loci detected by Omni-ATAC-seq, and transcription factor motif enrichments detected by Omni-ATAC-seq. The q-value represents the P value adjusted for multiple hypothesis testing using the Benjamini-Hochberg method to control the false discovery rate.

Quantification of mouse embryo images was performed as described in the above section, “Quantification of mouse embryo images”.

## Extended Data

## Supplementary Material

Supplement 1 **Table S1: Single-cell RNA-seq of hPSC-derived cell-types** Differentially expressed genes that distinguish day 0 hPSCs (“H1”), day 1 mid primitive streak (“d1ps”), day 2 lateral mesoderm (“d2dlm”), day 3 artery ECs (“d3aus”), day 3 pre-vein ECs (“d3pvus”), day 4 vein ECs (“d4vus”), and day 3–4 mesenchyme (“mes1”). Gene expression was measured by single-cell RNA-seq.
**Table S2: Bulk RNA-seq of hPSC-derived cell-types** Count matrix of gene expression of day-0 hPSCs (“H1”), day-1 mid primitive streak (“D1_PS”), day-2 lateral mesoderm (“D2_DLM”), day 3 artery ECs, CD144+ FACS-purified (“D3_Artery”), day 3 pre-vein ECs, CD144+ FACS-purified (“D3_Prevein”), and day 4 vein ECs, CD144+ FACS-purified (“D4_Vein”). Gene expression was measured by bulk-population RNA-seq, and is quantified in log_**2**_ counts per million (CPM) units.
**Table S3: Cell-surface marker screening of hPSC-derived cell-types** For each hPSC-derived cell-type, the number indicates the percentage of cells that expressed a given cell-surface marker by high-throughput flow cytometry. Day 3 artery EC and day 4 vein EC populations were pre-gated on the CD144+ endothelial subset before quantifying the expression of other cell-surface markers. This was done to avoid being confounded by CD144− non-endothelial cells in the culture.
**Table S4: Single-cell RNA-seq of E9.5 mouse embryo ECs *in vivo*** Differentially expressed genes that distinguish 4 different types of ECs identified in the E9.5 mouse embryo. Raw single-cell RNA-sequencing data were obtained from a previous study^65^. We provisionally annotated the following clusters based on expression of characteristic marker genes: 0 (vein-like 1 ECs), 1 (other ECs), 2 (artery ECs), and 3 (vein-like 2 ECs).
**Table S5: Single-cell RNA-seq of gene expression changes upon triple *SOXF* knockdown during artery EC differentiation** Genes that are differentially expressed between artery ECs generated from control vs. *SOX7/SOX17/SOX18*-CRISPRi hPSCs. Positive fold change indicates higher expression in control artery ECs, and log_**2**_ fold change is shown. Gene expression was measured by single-cell RNA-seq.
**Table S6: Single-cell RNA-seq of gene expression changes upon triple *SOXF* knockdown during vein EC differentiation** Genes that are differentially expressed between vein ECs generated from control vs. *SOX7/SOX17/SOX18*-CRISPRi hPSCs. Positive fold change indicates higher expression in control vein ECs, and log_**2**_ fold change is shown. Gene expression was measured by single-cell RNA-seq.
**Table S7: Differentially accessible genomic elements in hPSC-derived artery vs. vein ECs, as assayed by OmniATAC-seq** Differentially accessible genomic elements in hPSC-derived artery vs. vein ECs, as assayed by OmniATAC-seq. Fold change indicates accessibility in vein ECs relative to artery ECs (i.e., a positive value indicates that a genomic element is more accessible in vein ECs, whereas a negative value indicates greater chromatin accessibility in artery ECs). The gene closest to each genomic element is also indicated.
**Table S8: Transcription factors enriched in artery-specific regulatory elements, as assayed by OmniATAC-seq** Transcription factor motifs enriched in artery-specific regulatory elements, as determined by HOMER. Motifs are named in accordance with the most closely matching motif in the HOMER dataset. P value indicates the statistical significance of motif enrichment in artery-specific regulatory elements, relative to vein-specific regulatory elements.
**Table S9: Transcription factors enriched in vein-specific regulatory elements, as assayed by OmniATAC-seq** Transcription factor motifs enriched in vein-specific regulatory elements, as determined by HOMER. Motifs are named in accordance with the most closely matching motif in the HOMER dataset. P value indicates the statistical significance of motif enrichment in vein-specific regulatory elements, relative to artery-specific regulatory elements.
**Table S10: Bulk RNA-seq of hPSC-derived artery and vein ECs infected by Risk Group 4 viruses** Tab 1: count matrix of gene expression of hPSC-derived artery and vein ECs infected with either Sendai, Andes, Nipah, or Ebola viruses at 6, 12, 24, and 48 hours post-infection. Uninfected cells are designated as “Mock”. Each condition was assessed in triplicate. Tab 2: sample annotations. Gene expression was measured by bulk-population RNA-seq, and count units are shown.

## Figures and Tables

**Table:** 

Reagent or Resource	Source	Identifier
**Antibodies**		
CD144 FITC antibody (used 1:50 for *in vitro* studies)	BD Biosciences	560411
CD144 Alexa Fluor 647 antibody (used 1:50 for *in vitro* studies)	BD Biosciences	561567
DLL4 APC antibody (used 1:5 for *in vitro* studies)	BioLegend	346508
CXCR4 PE-Cy7 antibody (used 1:50 for *in vitro* studies)	BD Biosciences	560669
CD73 APC antibody (used 1:10 for *in vitro* studies)	BD Biosciences	560847
CD73 PE antibody (used 1:10 for *in vitro* studies)	Biolegend	344004
BST2/CD317 PE-Cy7 antibody (used 1:20 for *in vitro* studies)	BioLegend	348416
SOX17 antibody (used 1:1000 for *in vitro* studies and 1:200 for *in vivo* studies)	R&D Systems	AF-1924
ERG antibody (used 1:500 for *in vivo* studies)	Abcam	ab92513
VEGFR2 antibody (used 1:125 for *in vivo* studies)	R&D Systems	AF644
SMAα antibody Cy3 (used 1:500 for *in vivo* studies)	Sigma	C6198
GFP antibody (used 1:300 for *in vivo* studies)	Abcam	ab13970
GFP antibody (used 1:1000 for *in vivo* studies)	Aves Labs	NC9510598
Ebola virus NP antibody (used 1:2500 for *in vitro* studies)	Robert Koch Institute	Clone 173/303/109
TULV1 N antibody, cross-reactive against Andes virus (used 1:1000 for *in vitro* studies)	Rainer Ulrich’s laboratory	Avižinienė et al.^172^
Goat anti-mouse IgG secondary antibody Alexa Fluor 488 (used 1:500–1:1000 for *in vitro* studies)	Jackson ImmunoResearch	115-545-003
Donkey anti-chicken IgY secondary antibody Alexa Fluor 488 (used 1:250–1:400 for *in vivo* studies)	Thermo Fisher Scientific	A78948
Donkey anti-goat IgG secondary antibody Alexa Fluor 594 (used 1:500 for *in vivo* studies)	Thermo Fisher Scientific	A32758
Donkey anti-rabbit IgG secondary antibody Alexa Fluor 647 (used 1:500 for *in vivo* studies)	Thermo Fisher Scientific	A32795
**Bacterial and viral strains**		
Ebola virus, Yambuku variant, Mayinga isolate (Ebola virus/Human/COD/1976/Yambuku-Mayinga)	NIH, National Institute of Allergy and Infectious Diseases, Rocky Mountain Laboratories	Johnson et al.^129^
Andes virus, South variant, Chile-9717869 isolate (Andes virus/Oligoryzomys longicaudatus/CHL/1997/Chile-9717869)	NIH, National Institute of Allergy and Infectious Diseases, Rocky Mountain Laboratories	Toro et al.^131^ and Hooper et al.93
Sendai virus (Cantell strain)	ATCC	VR-907
Mix & Go! *E. coli* Competent Cells	Zymo Research	T3020
**Chemicals, peptides and recombinant proteins**		
mTesR Plus medium	STEMCELL Technologies	100-0276
Essential 8 medium	Thermo Fisher Scientific	A1517001
StemFlex medium	Thermo Fisher Scientific	A3349401
Penicillin/streptomycin	Thermo Fisher Scientific	15140163
Geltrex LDEV-free, hESC-qualified, reduced growth factor basement membrane matrix	Thermo Fisher Scientific	A1413302
Versene (EDTA-based dissociation buffer)	Thermo Fisher Scientific	15040066
Accutase (dissociation buffer)	Thermo Fisher Scientific	00-4555-56
TrypLE Express (dissociation buffer)	Thermo Fisher Scientific	12604013
EDTA (for flow cytometry buffer)	Thermo Fisher Scientific	15575020
DMEM/F12 + GlutaMAX	Thermo Fisher Scientific	10565042
IMDM + GlutaMAX	Thermo Fisher Scientific	31980-097
F12 + GlutaMAX	Thermo Fisher Scientific	31765-092
Polyvinyl alcohol	Sigma	P8136-250G
Chemically defined lipid concentrate	Thermo Fisher Scientific	11905-031
1-thioglycerol	Sigma	M6145-100ML
Recombinant human insulin	Sigma	11376497001
Human transferrin	Sigma	10652202001
Recombinant human Activin A	R&D Systems	338-AC-050
Recombinant human BMP4	R&D Systems	314-BP-050
Recombinant human FGF2	R&D Systems	233-FB-01M
Recombinant human VEGF	R&D Systems	293-VE-0500
CHIR99201	Tocris	4423
GDC-0941	Cellagen Technology	C4321-25
XAV939	Tocris	3748
SB505124	Tocris	3263
Forskolin	Tocris	1099
Ascorbic acid-2-phosphate (AA2P, Vitamin C analog)	Sigma	49752-10G
RO4929097	Cellagen Technology	C7649-10
DMH1	Tocris	4126
PD0325901	Tocris	4192
Thiazovivin	Tocris	3845
Dimethyl sulfoxide (DMSO)	Sigma	D2650
UltraPure DNase/RNase-free distilled water (H_2_O)	Thermo Fisher Scientific	10977023
Bovine albumin fraction V (7.5% solution), to reconstitute recombinant growth factors	Thermo Fisher Scientific	15260037
Endothelial Cell Growth Medium 2 (EGM2)	Lonza	CC-3162
Triton X-100	Sigma	X100-500ML
PBS, without Ca^2+^ or Mg^2+^, for non-virological experiments	Thermo Fisher Scientific	10010049
DPBS, without Ca^2+^ or Mg^2+^, for virological experiments	Thermo Fisher Scientific	14190144
DAPI, for *in vitro* studies	Thermo Fisher Scientific	D1306
DAPI, for *in vivo* studies	Sigma	D9542-1 MG
DMEM, for lentiviral packaging	Thermo Fisher Scientific	10566024
Fetal bovine serum, for lentiviral packaging	Atlanta Biologicals (later sold by R&D Systems)	S11550H
FuGENE HD Transfection Reagent, for lentiviral packaging	Promega	E2311
Poly-L-lysine, for lentiviral packaging	R&D Systems	3438-200-01
Opti-MEM, for lentiviral packaging	Thermo Fisher Scientific	31985070
Wizard Plus SV Minipreps DNA Purification System	Promega	A1460
Quick Ligation Kit	New England Biolabs	M2200
Hygromycin B	Thermo Fisher Scientific	10687010
Puromycin	Thermo Fisher Scientific	A1113803
Bovine serum albumin (BSA)	Sigma	A2153
Fluoromount-G mounting medium, for mouse embryology	SouthernBiotech	0100-01
32% paraformaldehyde (PFA), for mouse embryo immunostaining	Electron Microscopy Sciences	15714
32% paraformaldehyde (PFA), for mouse embryo *in situ* hybridization	Fisher Scientific	50-980-495
Methanol, for mouse embryo *in situ* hybridization	Fisher Scientific	A412-1
Tween 20, for mouse embryo *in situ* hybridization	Sigma	P9416-100ML
Glycerol, for mouse embryology	Thermo Fisher Scientific	327255000
Sucrose, for mouse embryology	Sigma	S0389
Normal donkey serum, freeze-dried, for mouse embryology	Jackson ImmunoResearch	102644-006
Tissue-Plus optimal cutting temperature (OCT) embedding medium, for mouse embryology	Fisher Scientific	23-730-571
(*Z*)-4-hydroxytamoxifen (4OHT), for *ApInr-CreER* lineage tracing	Cayman Chemical	14854
(*Z*)-4-hydroxytamoxifen (4OHT), for *Sox17-Cre;Aplnr-DreER* lineage tracing	Sigma	H7904
Ethanol, for lineage tracing	Fisher Scientific	BP2818500
Corn oil, for lineage tracing	Sigma	C8267
DMEM, for viral titration experiments	Robert Koch Institute	Produced in-house
Fetal bovine serum, for viral titration experiments	PAN Biotech	P30-3306
Stable glutamine, for viral titration experiments	PAN Biotech	P04-82100
Carboxymethylcellulose sodium salt, for viral titration experiments	Sigma	C4888-500G
Crystal violet, for viral titration experiments	Roth	T123.1
Methanol, for viral titration experiments	Roth	CP43.1
Tween-20, for viral titration experiments	Sigma	P9416
10% Formalin, for viral titration experiments	Sigma	F5554
**Critical commercial assays**		
Chromium Single Cell 3’ GEM, Library & Gel Bead Kit v3	10x Genomics	PN-1000075
Chromium Next GEM Single Cell 3’ GEM, Library & Gel Bead Kit v3.1	10x Genomics	PN-1000121
LEGENDScreen PE-Conjugated Human Antibody Plates	BioLegend	700007
Human IFNβ Quantikine QuicKit ELISA	R&D Systems	QK410
QIAamp Viral RNA Kit, for virological experiments	Qiagen	52904
AgPath-ID One-Step RT-PCR Kit, for virological experiments	Thermo Fisher Scientific	4387391
RNeasy Mini Kit, for virological experiments	Qiagen	74106
RNeasy Micro Kit, for virological experiments	Qiagen	74004
RNeasy Plus Mini Kit	Qiagen	10010049
High-Capacity cDNA Reverse Transcription Kit	Applied Biosystems	4368814
SensiFAST SYBR Lo-ROX Kit	Thomas Scientific	BIO-94050
Quick-RNA Microprep Kit	Zymo Research	R1051
Tagment DNA Enzyme 1 (TDE1)	Illumina	15027865
Tagment DNA Buffer	Illumina	15027866
MinElute PCR Purification Kit, for Omni-ATAC-seq	Qiagen	28204
AMPure XP Beads, for Omni-ATAC-seq	Beckman Coulter	A63880
CellBIND polystyrene CellSTACK chamber	Corning	3311
**Deposited data**		
Single-cell RNA-sequencing dataset of hPSCs differentiated into day 1 mid primitive streak, day 2 lateral mesoderm, day 3 artery ECs (unsorted cell population), day 3 (CD144^+^ DLL4^+^ CD73^lo/−^ FACS-purified), day 3 pre-vein ECs (unsorted cell population), day 3 pre-vein ECs (CD144^+^ FACS-purified), and day 4 vein ECs (CD144^+^ DLL4^−^ CD73^hi ^FACS-purified)	Kyle Loh’s and Lay Teng Ang’s laboratories (Stanford University)	This study
Single-cell RNA-sequencing dataset of hPSCs differentiated into day 4 vein ECs (unsorted cell population) using the Ang et al. protocol	Kyle Loh’s and Lay Teng Ang’s laboratories (Stanford University)	NCBI PRJNA837932 (Ang et al.^48^)
Single-cell RNA-seq of CRISPRi control vs. *SOX7/SOX17/SOX18*-knockdown hPSCs differentiated into day 3 artery ECs, day 3 pre-vein ECs, and day 4 vein ECs	Kyle Loh’s and Lay Teng Ang’s laboratories (Stanford University)	This study
Single-cell RNA-sequencing dataset of hPSCs differentiated into day 6 artery ECs using the Pan et al. protocol	Wei Kong’s, Xi Wang’s, and Kai Wang’s laboratories (Peking University)	NCBI PRJNA1114402 (Pan et al.^57^)
Single-cell RNA-sequencing dataset of hPSCs differentiated into day 6 vein ECs using the Pan et al. protocol	Wei Kong’s, Xi Wang’s, and Kai Wang’s laboratories (Peking University)	NCBI PRJNA1114402 (Pan et al.^57^)
Single-cell RNA-sequencing dataset of hPSCs differentiated into day 6 ECs using the Zhang et al. protocol	Andrew Baker’s laboratory (University of Edinburgh)	NCBI GSE131736 (McCracken et al.^61^)
Single-cell RNA-sequencing dataset of hPSCs differentiated into day 7 ECs using the McCracken et al. protocol	Andrew Baker’s laboratory (University of Edinburgh)	NCBI GSE131736 (McCracken et al.^61^)
Single-cell RNA-sequencing dataset of hPSCs differentiated into day 12 ECs using the Paik et al. protocol	Joseph Wu’s laboratory (Stanford University)	NCBI GSE116555 (Paik et al.^59^)
Single-cell RNA-sequencing dataset of hPSCs differentiated into day 14 ECs using the Nikolova et al. protocol	Josef Penninger’s, J. Gray Camp’s, and Barbara Treutlein’s laboratories (ETH Zurich, IMBA, and Roche)	Array Express E-MTAB-14807 (Nikolova et al.^58^)
Single-cell RNA-sequencing dataset of E9.5 mouse embryonic vasculature	William Pu’s laboratory (Boston Children’s Hospital)	NCBI GSE216970 (Chen et al.^65^)
Single-cell RNA-sequencing dataset of Carnegie Stage 12 human fetal vasculature	Vincenzo Calvanese’s and Hanna Mikkola’s laboratories (University of California, Los Angeles)	NCBI GSE162950 (Calvanese et al.^66^)
Bulk-population RNA-sequencing dataset of hPSCs differentiated into day 1 mid primitive streak, day 2 lateral mesoderm, and day 3 pre-vein ECs (CD144^+^ FACS-purified)	Kyle Loh’s and Lay Teng Ang’s laboratories (Stanford University)	This study
Bulk-population RNA-sequencing dataset of hPSCs differentiated into day 3 artery ECs (CD144^+^ DLL4^+^ CD73^lo/−^ FACS-purified) and day 4 vein ECs (CD144^+^ DLL4^−^ CD73^hi^ FACS-purified)	Kyle Loh’s and Lay Teng Ang’s laboratories (Stanford University)	NCBI PRJNA837932 (Ang et al.^48^)
OmniATAC-seq dataset of hPSCs differentiated into day 3 artery ECs (CD144^+^ DLL4^+^ CD73^lo/−^ FACS-purified), day 3 pre-vein ECs (CD144^+^ FACS-purified), and day 4 vein ECs (CD144^+^ DLL4^−^ CD73^hi^ FACS-purified)	Kyle Loh’s and Lay Teng Ang’s laboratories (Stanford University)	This study
CUT&RUN dataset of hPSCs differentiated into day 3 artery ECs (CD144^+^ DLL4^+^ CD73^lo/−^ FACS-purified), day 3 pre-vein ECs (CD144^+^ FACS-purified), and day 4 vein ECs (CD 144^+^ DLL4^−^ CD73^hi^ FACS-purified)	Kyle Loh’s and Lay Teng Ang’s laboratories (Stanford University)	This study
**Experimental models: cell lines**		
H1 hESCs	WiCell	WiCell, WA01 (Thomson et al.^125^)
H1 *NR2F2-2A-GFP; SOX17-2A-mPlum* knock-in reporter hESCs	This study	N/A
H1 CRISPRi-expressing hESCs	Richard She’s and Jonathan Weissman’s laboratories	N/A
WTC11 hiPSCs	Coriell Institute for Medical Research	Coriell Institute for Medical Research, GM25256, Kreitzer et al.^126^
SUN004.2 *CAG-mScarlet* hiPSCs	Hiro Nakauchi’s laboratory	Ang et al.^48^
HEK 293T/17 cells	ATCC	CRL-11268
Vero C1008 (Vero 76, clone E6, Vero E6)	ECACC	85020206
**Experimental models: organisms/strains**		
Wild-type CD-1 mice	Charles River	022
*Sox17-2A-iCre* mouse (otherwise known as *Sox17-Cre*)	Heiko Lickert’s laboratory	Engert et al.^67^
*Aplnr-CreER* mouse (otherwise known as *Apj-CreER*)	Kristy Red-Horse’s laboratory	Chen et al.^30^
*Aplnr-DreER* mouse (otherwise known as *Apj-DreER*)	Sophie Astrof’s laboratory	Ramirez et al.^71^
*ROSA26-LSL-zsGreen* mouse (otherwise known as Ai6)	The Jackson Laboratory (originally developed by the Allen Brain Institute)	JAX stock 007906 (Madisen et al.^122^)
*ROSA26-LSL-tdTomato* mouse (otherwise known as Ai14)	The Jackson Laboratory (originally developed by the Allen Brain Institute)	JAX stock 007914 (Madisen et al.^122^)
*RC::RLTG* mouse	The Jackson Laboratory (originally developed by Patricia Jensen’s laboratory)	JAX stock 026931 (Plummer et al.^72^)
*mTmG* mouse	The Jackson Laboratory (originally developed by Liqun Luo’s laboratory)	JAX stock 007676 (Muzumdar et al.^123^)
*Sema3g −13kB enhancer-LacZ* mouse	Len Pennacchio’s and Axel Visel’s laboratories	Kosicki et al.^85^
*Dll4 −12 kB enhancer-LacZ* mouse	Sarah De Val’s laboratory	Sacilotto et al.^81^
Wild-type AB strain zebrafish	Zebrafish International Resource Center	ZL1
*Cxcr4 +135 enhancer-GFP*; *kdrl-HRAS-mCherry* zebrafish	Sarah De Val’s laboratory	Nornes et al.^82^
**Oligonucleotides**		
Primers	See **Method details** section	This study
Pool of HCR3 *in situ* hybridization probes for human *APLNR* mRNA	Molecular Instruments	Custom order for human *APLNR* mRNA (NM_005161.6)
Pool of HCR3 *in situ* hybridization probes for zebrafish *sox7* mRNA	Molecular Instruments	Custom order
Pool of HCR3 *in situ* hybridization probes for zebrafish *aplnrb* mRNA	Molecular Instruments	Custom order
**Recombinant DNA**		
*mU6-sgRNA 1; hU6-sgRNA 2; EF1A-PuroR-2A*-GFP backbone plasmid, fragment 1	Addgene (originally developed by Jonathan Weissman’s laboratory)	Addgene, 187243 (Guna et al.^169^)
*mU6-sgRNA 1; hU6-sgRNA 2; EF1A-PuroR-2A*-GFP backbone plasmid, fragment 2	Addgene (originally developed by Jonathan Weissman’s laboratory)	Addgene, 187239 (Guna et al.^169^)
*mU6-sgRNA; EF1A-HygroR* plasmid	Karina Smolyar and Jonathan Weissman	This study
pVSV-G plasmid	Owen Witte’s laboratory	Goldstein et al.^174^
pMDL plasmid	Owen Witte’s laboratory	Goldstein et al.^174^
pREV plasmid	Owen Witte’s laboratory	Goldstein et al.^174^
**Software and algorithms**		
imageJ/Fiji	^134^	https://imagej.net/software/fiji/
Seurat v3	^121^	https://satijalab.org/seurat/
Seurat v4	^157^	https://satijalab.org/seurat/
RStudio	RStudio Team	https://www.rstudio.com/
Cell Ranger	10x Genomics (ref. ^152^)	https://support.10xgenomics.com/single-cell-gene-expression/software/pipelines/latest/what-is-cell-ranger
EnsDb.Hsapiens.v86	^149^	https://bioconductor.org/packages/release/data/annotation/html/EnsDb.Hsapiens.v86.html
DESeq2	^151^	https://github.com/mikelove/DESeq2
clusterProfiler	^154^	https://guangchuangyu.github.io/software/clusterProfiler/
ShinyCell	^155^	https://github.com/SGDDNB/ShinyCell
FastQC	^143^	http://www.bioinformatics.babraham.ac.uk/projects/fastqc/
Trim Galore	^144^	http://www.bioinformatics.babraham.ac.uk/projects/trim_galore/
Kallisto	^145^	https://pachterlab.github.io/kallisto/
EdgeR	^146^	http://bioconductor.org/packages/release/bioc/html/edgeR.html
Voom	^147^	https://rdrr.io/bioc/limma/man/voom.html
Limma	^148^	https://bioconductor.org/packages/release/bioc/html/limma.html
Dplyr	^135^	https://dplyr.tidyverse.org/
ggplot2	^136^	https://ggplot2.tidyverse.org/
Tidyverse	^137^	https://www.tidyverse.org/
FastQ Screen	^163^	https://www.bioinformatics.babraham.ac.uk/projects/fastq_screen/
NGmerge	^164^	https://github.com/harvardinformatics/NGmerge
SAMBLASTER	^165^	https://github.com/GregoryFaust/samblaster
SAMtools	^166^	https://github.com/samtools/samtools
deepTools	^167^	https://github.com/deeptools/deepTools
tximport	^150^	https://github.com/thelovelab/tximport
Diffbind	^161^	https://bioconductor.org/packages/release/bioc/html/DiffBind.html
HOMER	^162^	http://homer.ucsd.edu/homer/
pheatmap	^173^	https://github.com/raivokolde/pheatmap

## Data Availability

H1 *SOX17-2A-mPlum*; *NR2F2-2A-GFP* double reporter hESCs were generated as part of this study, and will be made freely available upon request and the completion of applicable Material Transfer Agreements. Ebola virus and Andes virus reference stocks were obtained from the CDC Viral Special Pathogens Branch and NIH/NIAID Rocky Mountain Laboratories. In accordance with U.S. federal law, requests for these viruses from these repositories must be approved by the U.S. Federal Select Agent Program, in compliance with biological security procedures that regulate the secure transfer of these viruses to authorized recipients within biosafety level 4 (BSL4) containment facilities.

## Data availability

Bulk-population RNA-seq, single-cell RNA-seq, bulk-population ATAC-seq, and bulk-population CUT&RUN datasets generated in this study will be made available upon acceptance. An interactive portal to access the single-cell RNA-seq dataset of hPSC differentiation into artery and vein ECs is available at https://anglab.shinyapps.io/artery-vein-scrna-seq/.

## Code availability

Computational scripts used for genomics analyses conducted in this study are available at GitHub: https://github.com/LayTengAngLab/Prevein_endothelial.
