# Supplementary material for: A human arteriovenous differentiation roadmap reveals vein developmental mechanisms and vascular effects of viruses
Source: bioRxiv. 2025 Oct 12:2025.10.11.681838. Preprint. [Version 1] doi: 10.1101/2025.10.11.681838 (PMC12632389; doi:10.1101/2025.10.11.681838)
Supplement: Supplementary file 1 [file NIHPP2025.10.11.681838v1-supplement-1.pdf]

bioRxiv preprint doi: https://doi.org/10.1101/2025.10.11.681838; this version posted October 12, 2025. The copyright holder for this preprint (which was not certified by peer review) is the author/funder, who has granted bioRxiv a license to display the preprint in perpetuity. It is made available under aCC-BY 4.0 International license.

Figure S1

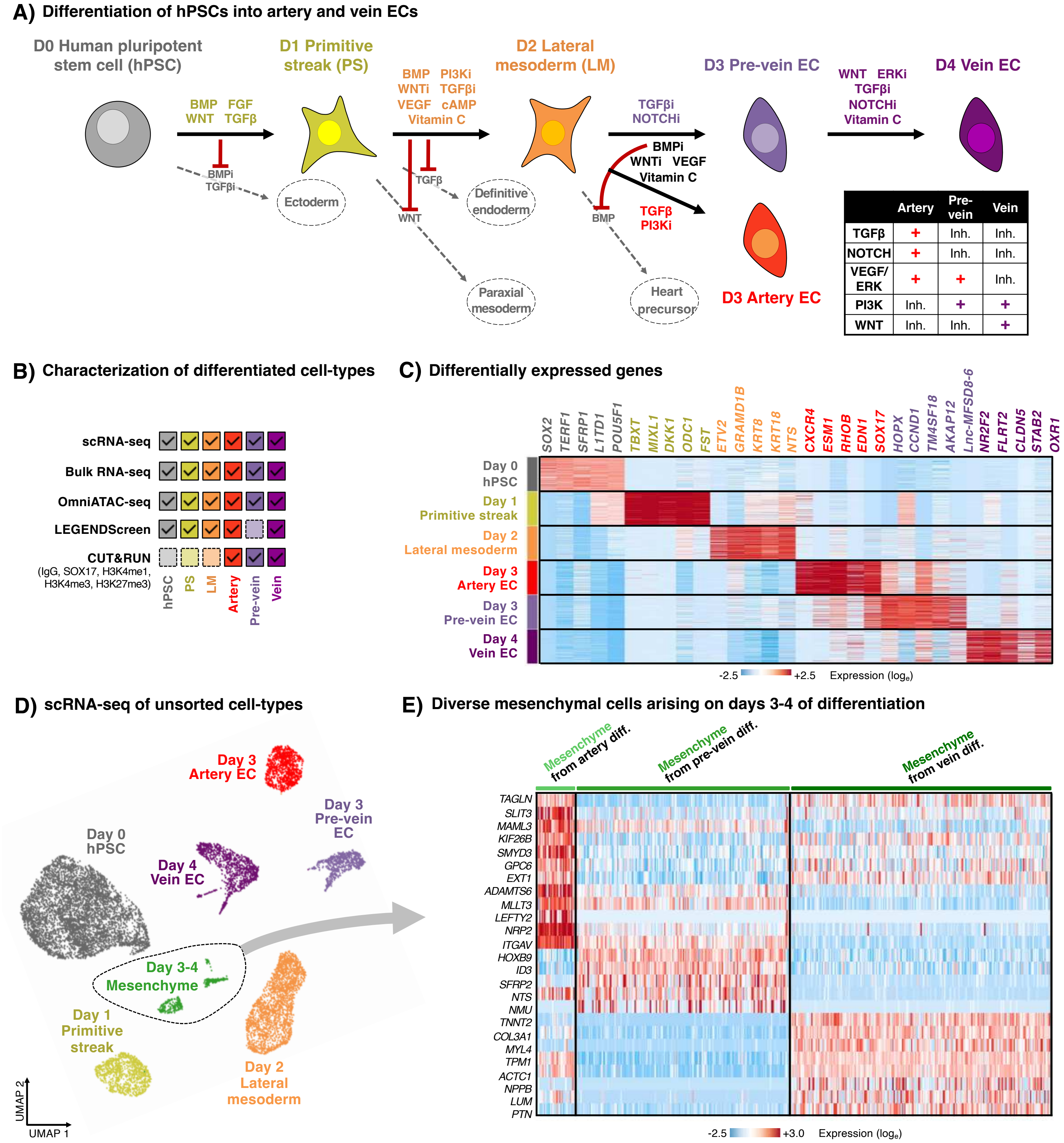

bioRxiv preprint doi: https://doi.org/10.1101/2025.10.11.681838; this version posted October 12, 2025. The copyright holder for this preprint (which was not certified by peer review) is the author/funder, who has granted bioRxiv a license to display the preprint in perpetuity. It is made available under aCC-BY 4.0 International license.

Figure S2

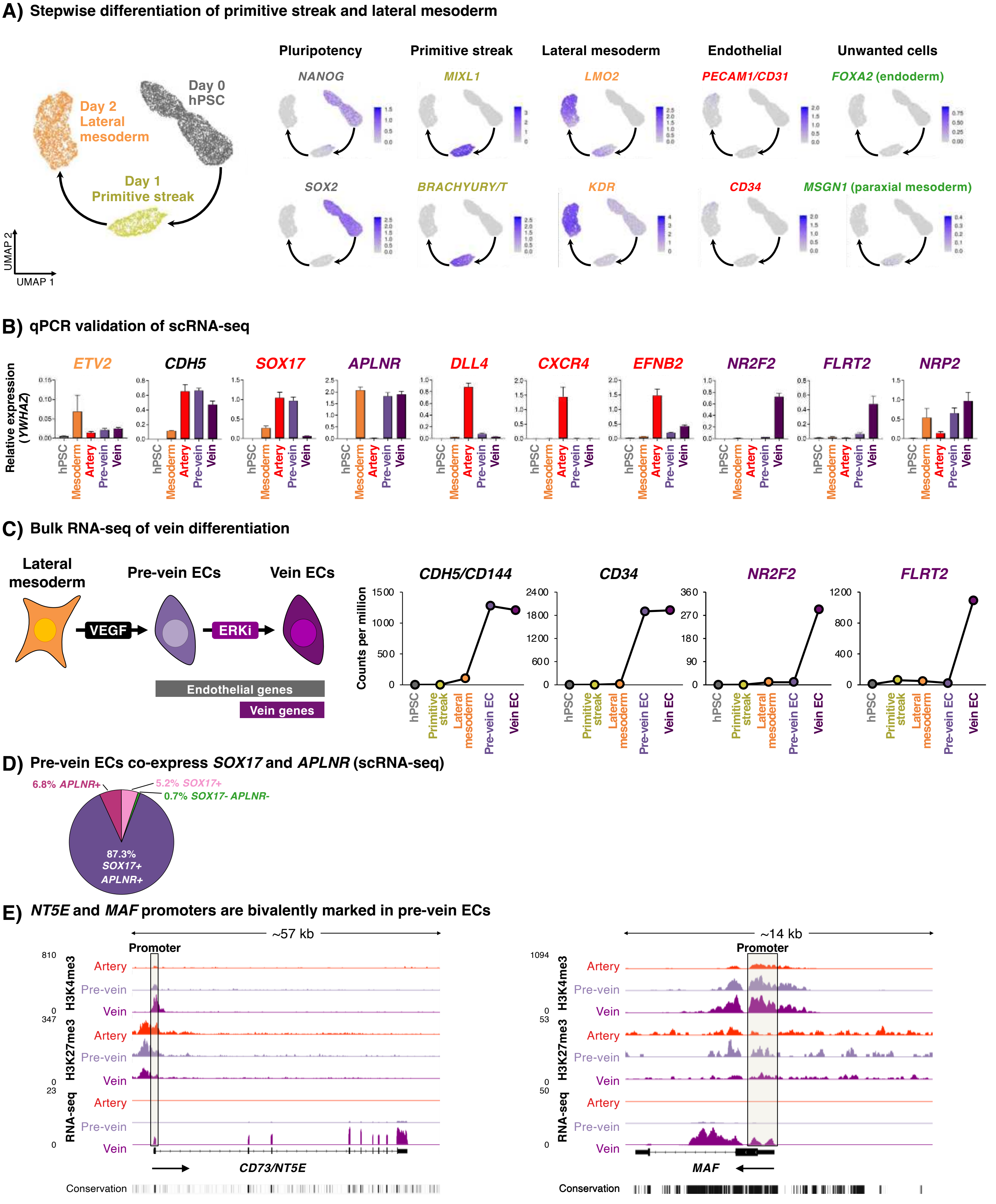

bioRxiv preprint doi: https://doi.org/10.1101/2025.10.11.681838; this version posted October 12, 2025. The copyright holder for this preprint (which was not certified by peer review) is the author/funder, who has granted bioRxiv a license to display the preprint in perpetuity. It is made available under aCC-BY 4.0 International license.

Figure S3

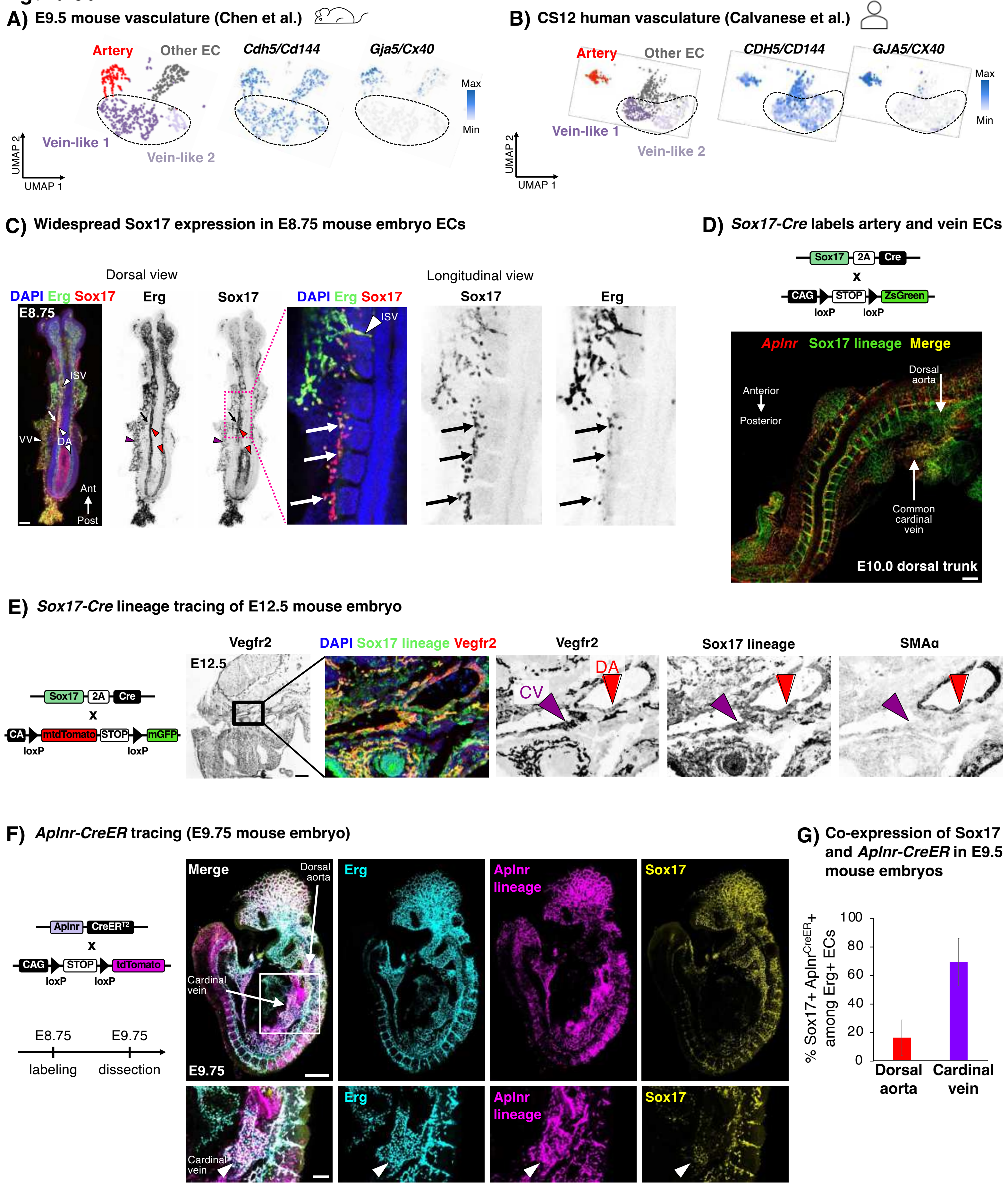

bioRxiv preprint doi: https://doi.org/10.1101/2025.10.11.681838; this version posted October 12, 2025. The copyright holder for this preprint (which was not certified by peer review) is the author/funder, who has granted bioRxiv a license to display the preprint in perpetuity. It is made available under aCC-BY 4.0 International license.

Figure S4

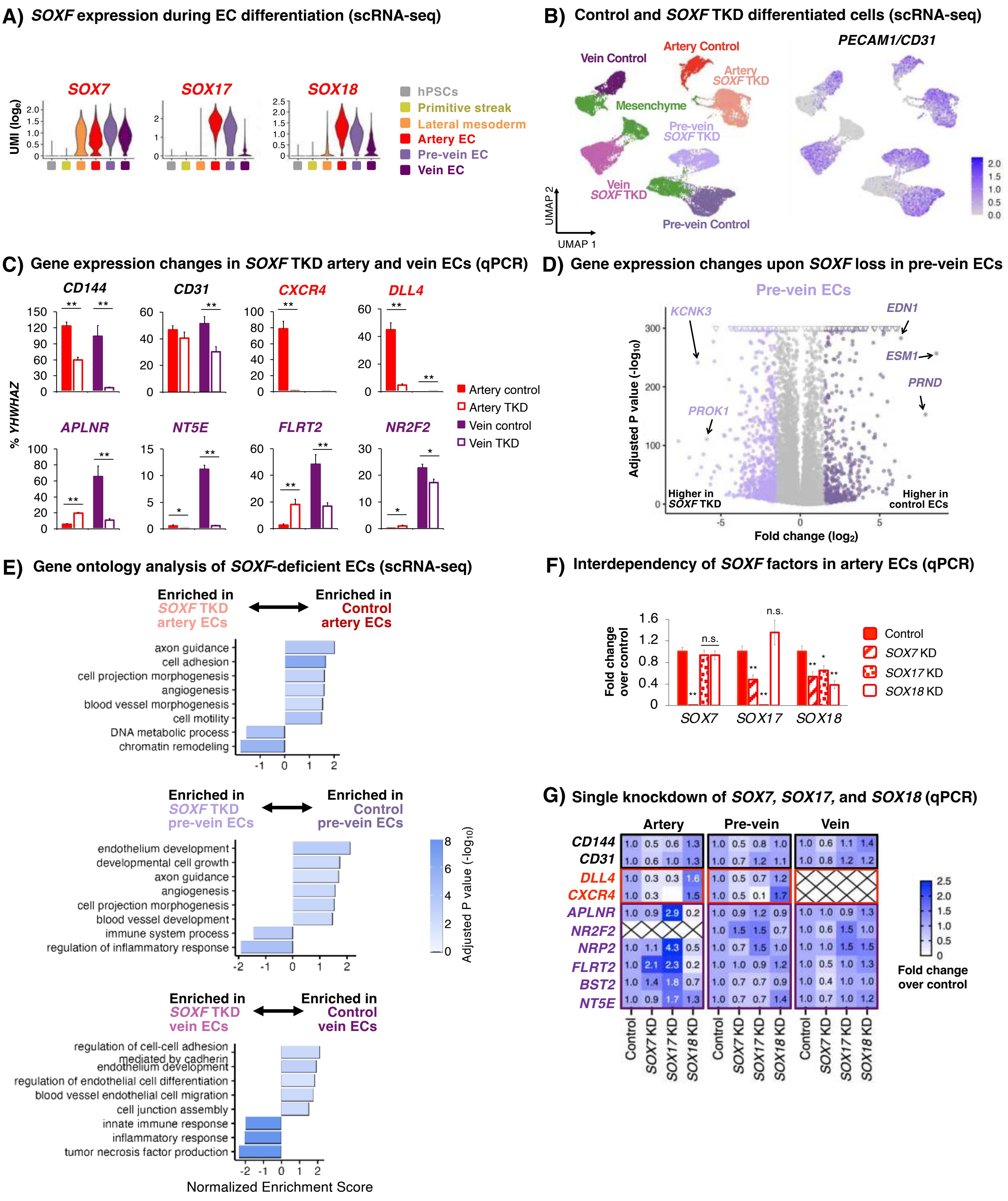

bioRxiv preprint doi: https://doi.org/10.1101/2025.10.11.681838; this version posted October 12, 2025. The copyright holder for this preprint (which was not certified by peer review) is the author/funder, who has granted bioRxiv a license to display the preprint in perpetuity. It is made available under aCC-BY 4.0 International license.

Figure S5

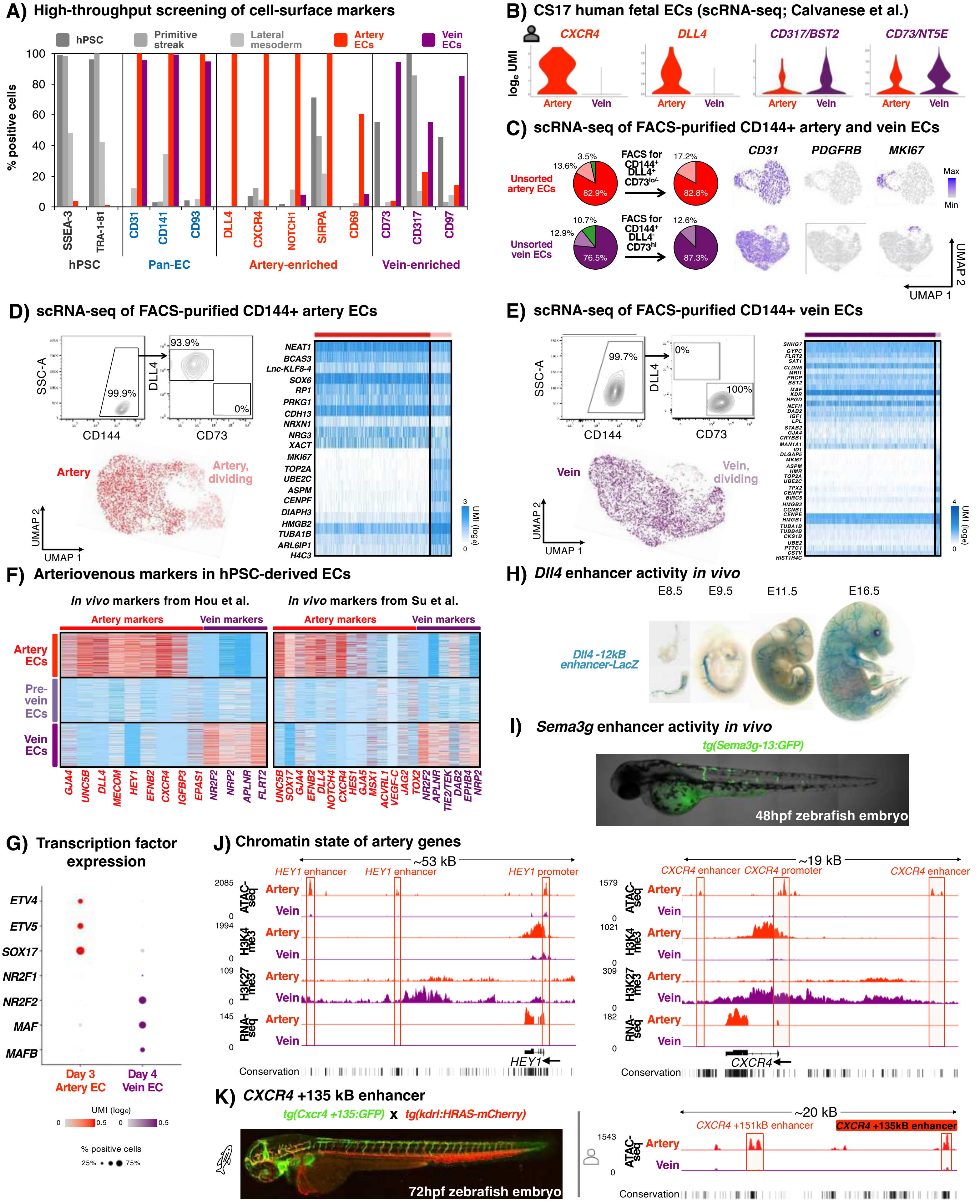

bioRxiv preprint doi: https://doi.org/10.1101/2025.10.11.681838; this version posted October 12, 2025. The copyright holder for this preprint (which was not certified by peer review) is the author/funder, who has granted bioRxiv a license to display the preprint in perpetuity. It is made available under aCC-BY 4.0 International license.

Figure S6

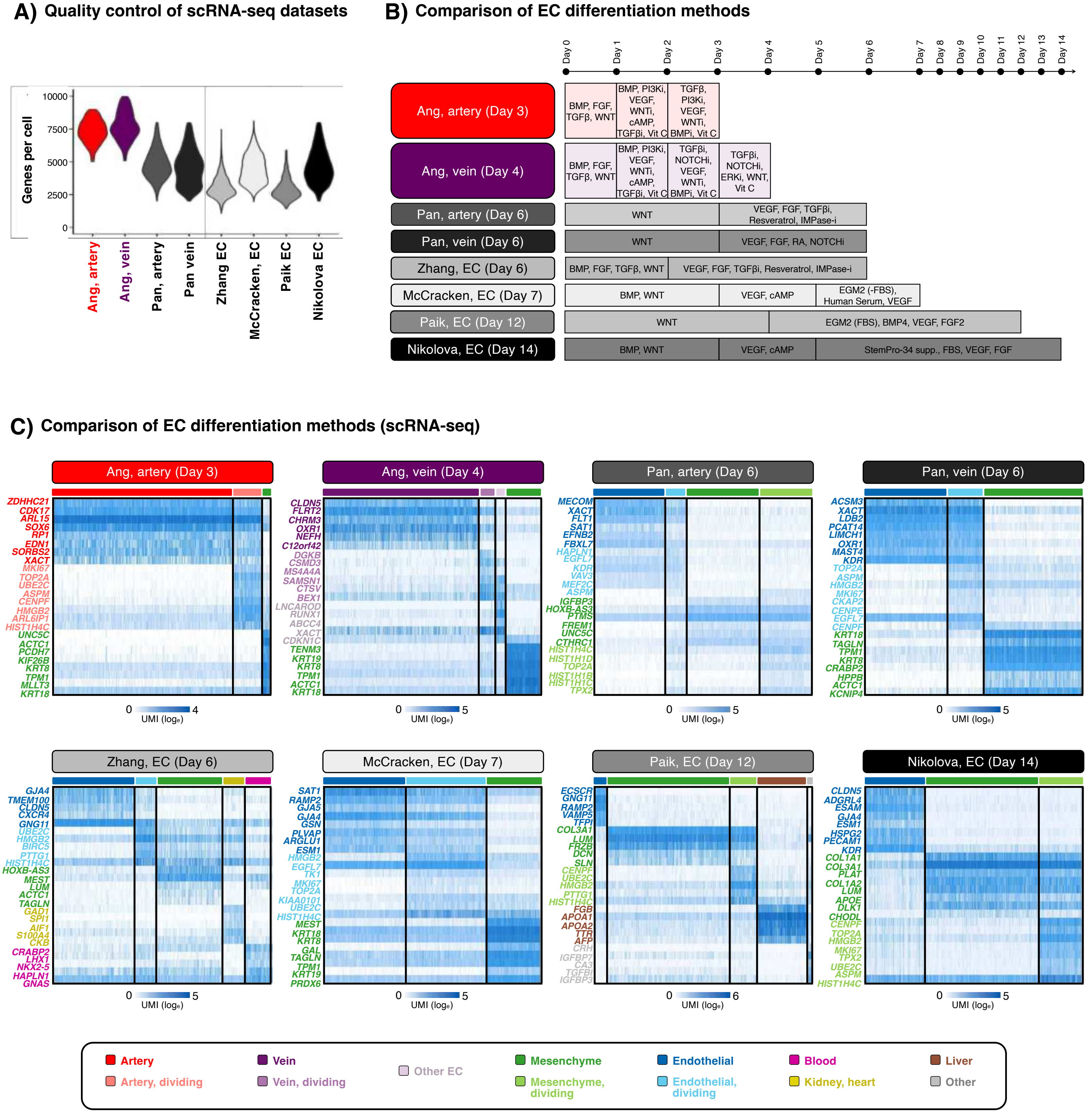

bioRxiv preprint doi: https://doi.org/10.1101/2025.10.11.681838; this version posted October 12, 2025. The copyright holder for this preprint (which was not certified by peer review) is the author/funder, who has granted bioRxiv a license to display the preprint in perpetuity. It is made available under aCC-BY 4.0 International license.

Figure S7

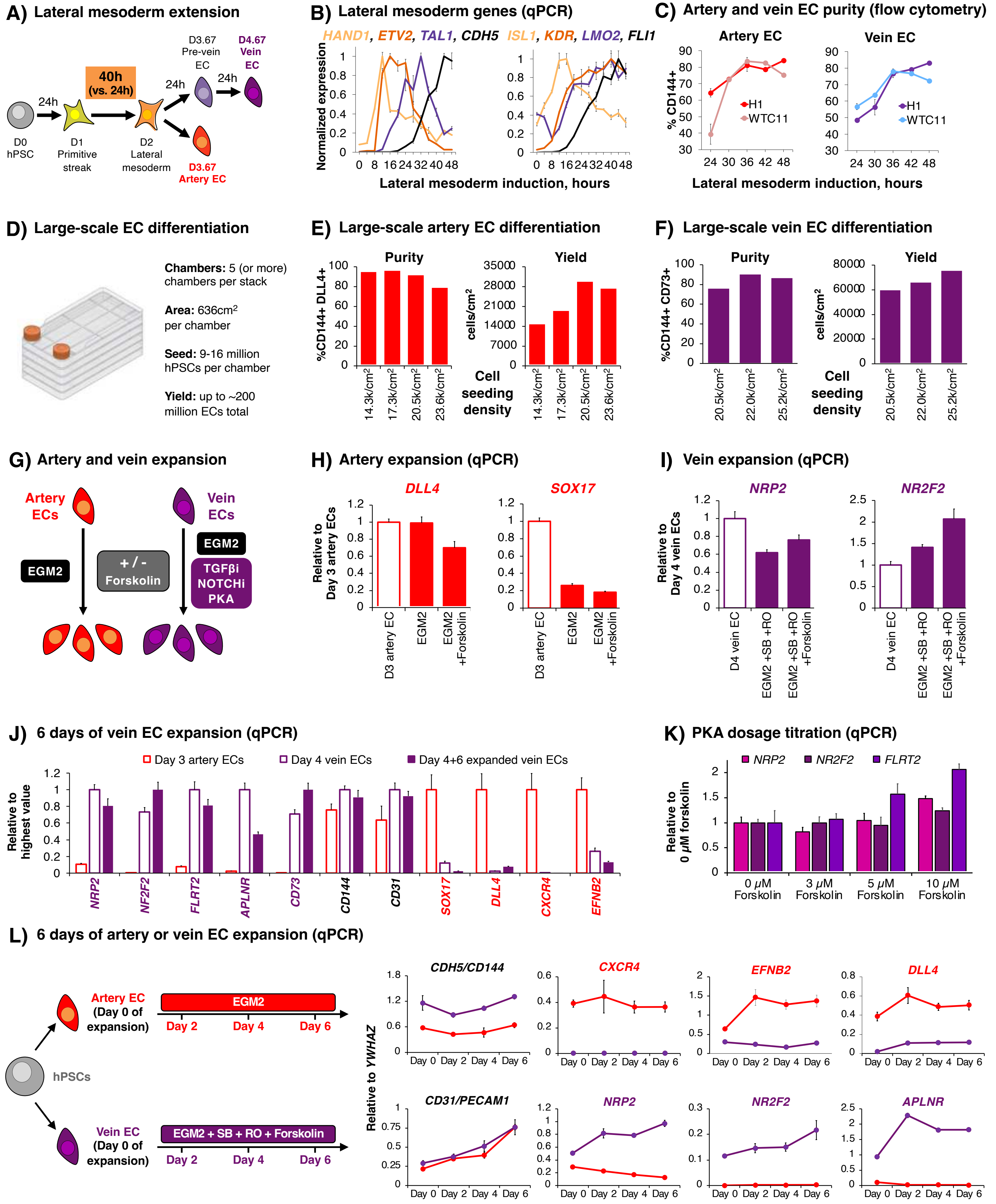

bioRxiv preprint doi: https://doi.org/10.1101/2025.10.11.681838; this version posted October 12, 2025. The copyright holder for this preprint (which was not certified by peer review) is the author/funder, who has granted bioRxiv a license to display the preprint in perpetuity. It is made available under aCC-BY 4.0 International license.

Figure S8

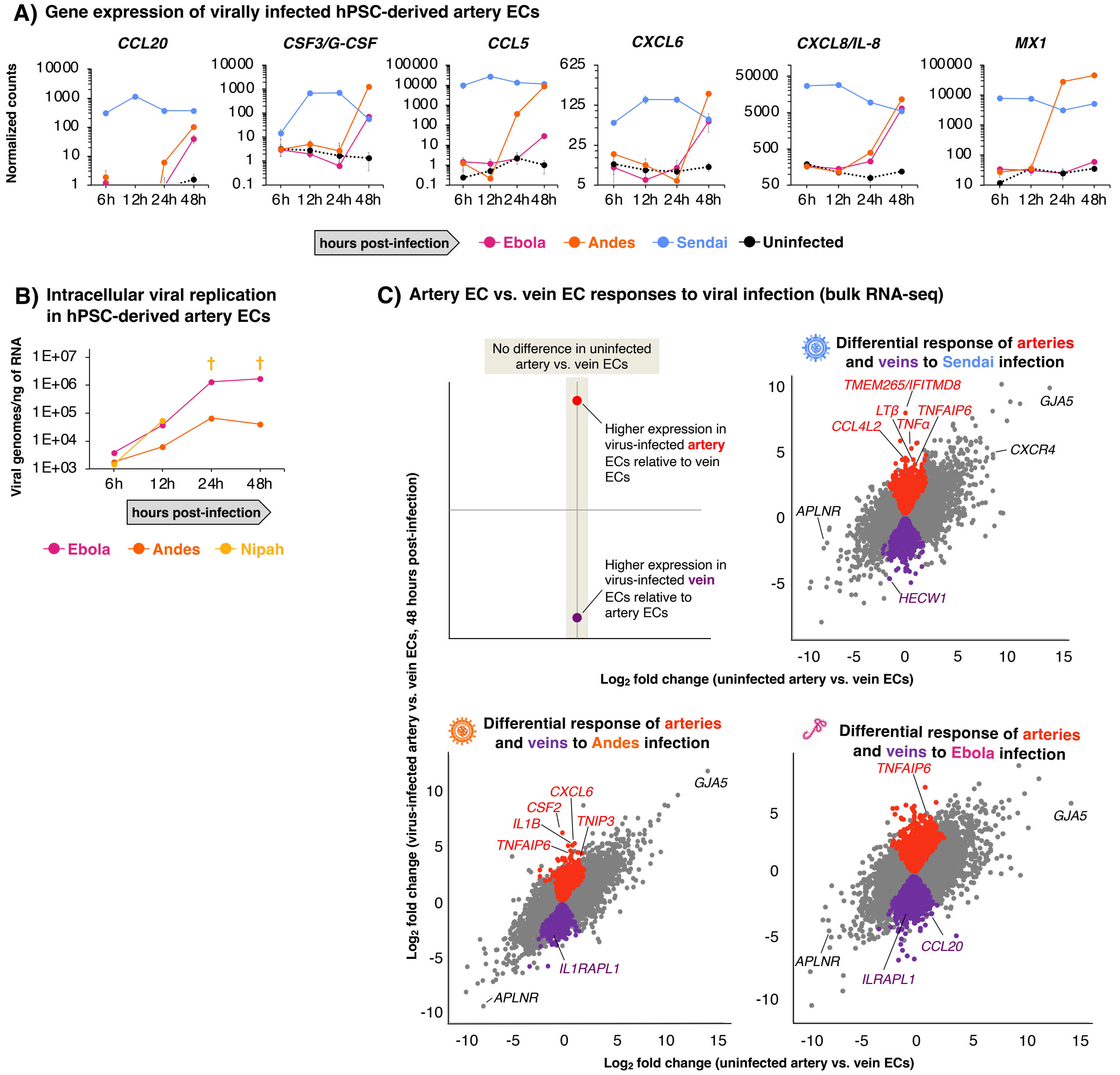

bioRxiv preprint doi: https://doi.org/10.1101/2025.10.11.681838; this version posted October 12, 2025. The copyright holder for this preprint (which was not certified by peer review) is the author/funder, who has granted bioRxiv a license to display the preprint in perpetuity. It is made available under aCC-BY 4.0 International license.

SUPPLEMENTAL FIGURE LEGENDS

Figure S1: Cellular diversity during early steps of hPSC differentiation, related to Figure 1

A) Summary of hPSC differentiation approach[49]. Inh. and i: inhibition.
B) Summary of gene expression, chromatin state, and cell-surface marker profiling conducted as part of this study. Dotted lines indicate that profiling was not conducted on a given cell-type.
C) Differentially expressed genes that distinguish each hPSC-derived cell-type, as detected by scRNAseq. scRNA-seq was performed on day-0 H1 hPSCs, day-1 primitive streak, day-2 lateral mesoderm, CD144+ FACS-purified day-3 artery ECs, CD144+ FACS-purified day-3 pre-vein ECs, and CD144+ FACS-purified day-4 vein ECs.
D) scRNAseq of day-0 H1 hPSCs that were differentiated into day-1 primitive streak, day-2 lateral mesoderm, day-3 artery ECs, day-3 pre-vein ECs, and day-4 vein ECs. None of cell populations shown here were FACS purified and consequently, mesenchymal cells were detected alongside artery, pre-vein, and vein ECs on days 3-4 of differentiation. The same day-0 hPSC, day-1 primitive streak, and day-2 primitive streak scRNAseq datasets were also used in **Fig. 1B**.
E) scRNAseq of H1 hPSC-derived mesenchymal cells that arose alongside day-3 artery ECs, day-3 pre-vein ECs, and day-4 vein ECs. Differentially expressed genes that distinguish these three different types of mesenchyme are shown. Diff: differentiation.

Figure S2: Characterization of pre-vein endothelial cells *in vitro*, related to Figure 2

A) Gene expression on days 0, 1, and 2 of H1 hPSC differentiation, as detected by scRNAseq.
B) qPCR of the indicated H1 hPSC-derived cell-types. qPCR data normalized to reference gene *YWHAZ* (i.e., *YWHAZ* levels = 1.0). Error bars: standard deviation (SD).
C) Bulk RNA-seq of the indicated H1 hPSC-derived cell-types. FACS was used to purify CD144+ pre-vein and vein ECs for RNA-seq. i: inhibition.
D) scRNAseq of H1 hPSC-derived day-3 pre-vein EC populations, showing the percentage of cells that expressed *APLNR* and/or *SOX17* mRNAs.
E) CUT&RUN profiling of H3K4me3 and H3K27me3 and bulk RNA-seq H1 hPSC-derived day-3 artery ECs, day-3 pre-vein ECs, and day-4 vein ECs. Conservation: Phastcons evolutionary conservation of genome sequence across 46 vertebrate species.

Figure S3: Existence and fate of *Sox17*+ *Aplnr*+ endothelial cells in the mouse embryo, related to Figure 3

A) scRNAseq of E9.5 mouse embryo ECs. scRNAseq data were obtained from Chen et al., 2024[65].
B) scRNAseq of Carnegie Stage 12 (CS12) human embryo ECs. scRNAseq data were obtained from Calvanese et al., 2022[66].
C) Whole-mount Erg and Sox17 immunostaining of E8.75 mouse embryo. DA: dorsal aorta (arrowhead). VV: vitelline vein (arrowhead). ISV: intersomitic vessel (arrowhead). Arrows: solitary Sox17+ Erg+ ECs. Ant: anterior. Post: posterior. Scale: 100 μm.
D) Whole-mount *Aplnr* mRNA staining of E10 $Sox17^{Cre}$; $R26^{zsGreen}$ mouse embryos[67,137] by HCR3 *in situ* hybridization. Scale: 100 μm.

bioRxiv preprint doi: https://doi.org/10.1101/2025.10.11.681838; this version posted October 12, 2025. The copyright holder for this preprint (which was not certified by peer review) is the author/funder, who has granted bioRxiv a license to display the preprint in perpetuity. It is made available under aCC-BY 4.0 International license.

E) E12.5 *Sox17^{Cre}; R26^{mTmG}* mouse embryos[67,138] were sectioned and stained for Vegfr2, SMAα, and GFP proteins. Scale: 200 μm.

F) 4-hydroxytamoxifen (4OHT) was administered *in utero* to E8.75 *Aplnr-CreER*; *R26-tdTomato* mouse embryos[17,137], which were then isolated at E9.75 and immunostained for Sox17 and Erg proteins. Bottom row arrows: cardinal vein. Scale: 200 μm (top row), 50 μm (bottom row).

G) 4-hydroxytamoxifen (4OHT) was administered *in utero* to E8.75 *Aplnr-CreER*; *R26-tdTomato* mouse embryos[17,137], which were then isolated at E9.75 and immunostained for Sox17 and Erg proteins. Quantification of Erg+ ECs in the dorsal aorta and cardinal vein that co-expressed tdTomato (indicative of *Aplnr-CreER* activity) and Sox17. Error bars: SD. **P<0.01.

Figure S4: Triple CRISPRi knockdown of *SOXF* transcription factors impairs both human artery and vein EC differentiation *in vitro*, related to Figure 4.

A) scRNA-seq of day-2 lateral mesoderm, CD144+ FACS-purified day-3 artery ECs, CD144+ FACS-purified day-3 pre-vein ECs, and CD144+ FACS-purified day-4 vein ECs.

B) *Left*: scRNA-seq of day-3 artery ECs, day-3 pre-vein ECs and day-4 vein ECs generated from either H1 control or *SOXF* TKD CRISPRi hPSCs. *Right*: *PECAM1*+ ECs and *PECAM1*- mesenchymal cells were detected. The same scRNAseq datasets are shown here as in **Fig. 4C**, except that mesenchymal cells were computationally excluded in **Fig. 4C**.

C) qPCR of day-3 artery ECs and day-4 vein ECs generated from H1 control vs. *SOXF* TKD CRISPRi hPSCs. Gene expression normalized to reference gene *YWHAZ* (i.e., *YWHAZ* = 100%). Statistics: unpaired t-test. Error bars: SEM. *P<0.05. **P<0.01.

D) Differentially expressed genes between day-3 pre-vein ECs generated from H1 control vs. *SOXF* TKD CRISPRi hPSCs are colored.

E) Gene Set Enrichment analysis (GSEA)[139] of scRNA-seq data from H1 CRISPRi control vs. *SOXF* TKD hPSCs differentiated into day-3 artery ECs, day-3 pre-vein ECs, and day-4 vein ECs. Color represents the $\log_{10}$-transformed adjusted P-value.

F) qPCR of day-3 artery ECs derived from control and single *SOX7*-, *SOX17*-, or *SOX18*-knockdown H1 CRISPRi hPSC lines. Gene expression normalized to levels in control hPSC-derived artery ECs, which was set as "1.0". Statistics: unpaired t-test. Error bars: SEM. n.s.: not significant. *P<0.05. **P<0.01.

G) qPCR of day-3 artery ECs derived from control and single *SOX7*-, *SOX17*-, or *SOX18*-knockdown H1 CRISPRi hPSC lines. Gene expression normalized to levels in control hPSC-derived artery ECs, which was set as "1.0". "X" indicates that qPCR data were not shown, because gene expression was under 2% of *YWHAZ* in control samples. Statistics: unpaired t-test. **P<0.01.

Figure S5: Transcriptional, chromatin, and surface-marker differences between artery and vein ECs, related to Figure 5

A) LEGENDScreen high-throughput flow cytometry of cell-surface markers in day-0 hPSCs, day-1 primitive streak, day-2 lateral mesoderm, day-3 artery ECs, and day-4 vein ECs. Day-3 artery and day-4 vein populations were pre-gated on the CD144+ EC subset before depicting marker expression.

bioRxiv preprint doi: https://doi.org/10.1101/2025.10.11.681838; this version posted October 12, 2025. The copyright holder for this preprint (which was not certified by peer review) is the author/funder, who has granted bioRxiv a license to display the preprint in perpetuity. It is made available under aCC-BY 4.0 International license.

B) scRNAseq of human Carnegie Stage 17 (CS17) fetal ECs. scRNAseq data were obtained from Calvanese et al., 2022[66].
C) scRNAseq of FACS-purified CD144+ DLL4+ CD73lo/- day-3 artery ECs and CD144+ DLL4- CD73hi day-4 vein ECs generated from H1 hPSCs.
D) scRNAseq of FACS-purified CD144+ DLL4+ CD73lo/- day-3 artery ECs generated from H1 hPSCs. Left: FACS isolation strategy. Right: subclustering was performed to assess any potential population heterogeneity, and differentially expressed genes that distinguished cell subsets are shown.
E) scRNAseq of FACS-purified CD144+ DLL4- CD73hi day-4 vein ECs generated from H1 hPSCs. Left: FACS isolation strategy. Right: subclustering was performed to assess any potential population heterogeneity, and differentially expressed genes that distinguished cell subsets are shown.
F) scRNAseq of FACS-purified CD144+ DLL4+ CD73lo/- day-3 artery ECs, CD144+ day-3 pre-vein ECs, and CD144+ DLL4- CD73hi day-4 vein ECs generated from H1 hPSCs. In each of these *in vitro* cell-types, expression of *in vivo* arterial and venous markers reported by Hou et al., 2022[21] and Su et al., 2018[20] is shown.
G) Expression of *ETV*, *SOX*, *NR2F*, and *MAF* family transcription factors at the mRNA level, as shown by scRNAseq of FACS-purified CD144+ DLL4+ CD73lo/- day-3 artery ECs and CD144+ DLL4- CD73hi day-4 vein ECs generated from H1 hPSCs.
H) LacZ staining of E8.5-E16.5 mouse embryos bearing a *Dll4* -12 kB enhancer transgene driving *LacZ* expression[79].
I) Image of a 2 day-post-fertilization (dpf) zebrafish bearing a *Sema3g* -13 kB enhancer driving *GFP* expression.
J) OmniATAC-seq, CUT&RUN, and bulk RNA-seq of H1 hPSC-derived day-3 artery ECs and day-4 vein ECs. Conservation: Phastcons evolutionary conservation of genome sequence across 46 vertebrate species.
K) Left: image of a 3 day-post-fertilization (dpf) zebrafish bearing a *Cxcr4* +135 kB enhancer driving *GFP* expression, together with a *kdrl:HRAS-mCherry* transgene to label ECs[84]. Right: ATAC-seq of H1 hPSC-derived day-3 artery ECs and day-4 vein ECs, highlighting the *CXCR4* +135 kB enhancer whose ortholog was tested in the zebrafish transgenic assay[84]. Conservation: Phastcons evolutionary conservation of genome sequence across 46 vertebrate species.

Figure S6: Comparison of methods to differentiate hPSCs into ECs, related to Figure 6

A) scRNA-seq of hPSCs differentiated into ECs using various protocols, depicting the number of genes detected per single cell. This represents the quality control assessment for scRNA-seq datasets described in **Fig. 6A**.
B) Summary of protocols to differentiate hPSCs into ECs[49,60,61,85-87] used to generate the scRNA-seq datasets described in **Fig. 6A**.
C) scRNA-seq of differentiated hPSC populations described in **Fig. 6A**. Subclustering was performed to assess population heterogeneity, and differentially expressed genes that distinguished cell subsets are shown.

Figure S7: Scaling up hPSC differentiation into artery and vein ECs, and maintenance of arteriovenous identity, related to Figures 6 and 7

bioRxiv preprint doi: https://doi.org/10.1101/2025.10.11.681838; this version posted October 12, 2025. The copyright holder for this preprint (which was not certified by peer review) is the author/funder, who has granted bioRxiv a license to display the preprint in perpetuity. It is made available under aCC-BY 4.0 International license.

A) Schematic of differentiation protocol, which includes prolongation of lateral mesoderm induction from 24 hours (V1 protocol) to 40 hours (V2 protocol).
B) qPCR of lateral mesoderm and vascular marker expression in H1 hPSC-derived day-1 primitive streak cells challenged with lateral mesoderm induction media for up to 48 hours. 0 hours corresponds to day-1 primitive streak cells, prior to addition of lateral mesoderm induction media. Cells were lysed every 4 hours and qPCR was performed. For each gene, expression is normalized to the sample with the highest value, which was set to "1.0". Error bars: SEM.
C) Effect of lateral mesoderm induction duration on the subsequent production of CD144+ artery and vein ECs from the H1 and WTC11 hPSC lines, as measured by flow cytometry. Duration of lateral mesoderm induction was varied from 24 hours (V1 protocol) to 48 hours, with all other parameters of the differentiation protocol unchanged. Error bars: SEM.
D) Schematic of CellSTACK system for large-scale differentiation of hPSCs into ECs.
E) Yield and percentage of CD144+ DLL4+ day-3.67 artery ECs generated in the large-scale CellSTACK differentiation system, depending on initial hPSC seeding density. Yield and differentiation purity were respectively measured by flow cytometry and cell counting.
F) Yield and percentage of CD73+ DLL4+ day-4.67 vein ECs generated in the large-scale CellSTACK differentiation system, depending on initial hPSC seeding density. Yield and differentiation purity were respectively measured by flow cytometry and cell counting.
G) Strategy for expanding hPSC-derived artery and vein ECs. Artery ECs are cultured in EGM2 medium[49], and vein ECs are cultured in EGM2 medium + TGFβ inhibitor + NOTCH inhibitor + PKA activator. i: inhibitor.
H) qPCR of H1 hPSC-derived artery ECs that were thawed in EGM2 medium[49] and cultured for 6 days. ROCK inhibitor (Thiazovivin, 2 μM) was added for the first day of thawing to improve cell survival, and was subsequently removed in later days. Forskolin (3 μM) was added as indicated. For each gene, expression is normalized to the sample with the highest value, which was set to "1.0". Error bars: SEM.
I) qPCR of H1 hPSC-derived vein ECs that were thawed in EGM2 medium + SB505124 (2 μM, TGFβ inhibitor) + RO4929097 (1 μM, NOTCH inhibitor)[49] and cultured for 6 days. ROCK inhibitor (Thiazovivin, 2 μM) was added for the first day of thawing to improve cell survival, and was subsequently removed in later days. Forskolin (3 μM) was added as indicated. For each gene, expression is normalized to the sample with the highest value, which was set to "1.0". Error bars: SEM.
J) qPCR of H1 hPSC-derived vein ECs that were thawed in EGM2 medium + SB505124 (2 μM, TGFβ inhibitor) + RO4929097 (1 μM, NOTCH inhibitor) + Forskolin (10 μM, adenylate cyclase agonist) and cultured for 6 days. ROCK inhibitor (Thiazovivin, 2 μM) was added for the first day of thawing to improve cell survival, and was subsequently removed in later days. As a control, H1 hPSC-derived artery ECs prior to expansion were also analyzed. qPCR data are normalized to the sample with the highest expression, which was set to "1.0". Error bars: SEM.
K) qPCR of H1 hPSC-derived vein ECs that were thawed in EGM2 medium + SB505124 (2 μM, TGFβ inhibitor) + RO4929097 (1 μM, NOTCH inhibitor), in the presence or absence of Forskolin (3-10 μM, adenylate cyclase agonist) and cultured for 6 days.

bioRxiv preprint doi: https://doi.org/10.1101/2025.10.11.681838; this version posted October 12, 2025. The copyright holder for this preprint (which was not certified by peer review) is the author/funder, who has granted bioRxiv a license to display the preprint in perpetuity. It is made available under aCC-BY 4.0 International license.

qPCR data are normalized to the sample lacking forskolin, which was set to “1.0”. Error bars: SEM.

L) qPCR of pan-endothelial, arterial, and venous marker expression over the course of 6-day expansion of SUN004.2 *CAG-mScarlet* hPSC-derived artery and vein ECs. Artery ECs were expanded in EGM2 medium, whereas vein ECs were expanded in EGM2 medium + SB505124 (2 μM, TGFβ inhibitor) + RO4929097 (1 μM, NOTCH inhibitor) + Forskolin (10 μM, adenylate cyclase agonist). qPCR data normalized to reference gene *YWHAZ* (i.e., *YWHAZ* levels = 1.0). Error bars: SEM.

Figure S8: Responses of artery and vein ECs to infection by Risk Group 4 viruses, related to Figure 7

A) Bulk RNA-seq of hPSC-derived artery ECs 6, 12, 24, and 48 hours after infection with Ebola, Andes, and Sendai viruses, or left uninfected (mock control). Error bars: SEM.

B) Replication of Andes, Nipah, and Ebola viruses in hPSC-derived artery and vein ECs, as assayed by qPCR for intracellular viral genomes. †: extensive cell death, and therefore RNA was not quantified at the indicated timepoints.

C) Bulk RNA-seq of artery and vein ECs 48 hours after infection with Sendai, Andes, Nipah, and Ebola viruses. Genes that are differentially expressed between infected artery and vein ECs, but are not differentially expressed between uninfected artery and vein ECs are correspondingly colored. Red: genes more highly induced by viral infection in hPSC-derived artery relative to vein ECs, and which do not show substantially different expression in uninfected artery vs. vein ECs. Purple: genes more highly induced by viral infection in hPSC-derived vein relative to artery ECs, and which do not show substantially different expression in uninfected artery vs. vein ECs.

bioRxiv preprint doi: https://doi.org/10.1101/2025.10.11.681838; this version posted October 12, 2025. The copyright holder for this preprint (which was not certified by peer review) is the author/funder, who has granted bioRxiv a license to display the preprint in perpetuity. It is made available under aCC-BY 4.0 International license.

INVENTORY OF SUPPLEMENTAL INFORMATION

Table S1: **Single-cell RNA-seq of hPSC-derived cell-types**
Differentially expressed genes that distinguish day 0 hPSCs ("H1"), day 1 mid primitive streak ("d1ps"), day 2 lateral mesoderm ("d2dlm"), day 3 artery ECs ("d3aus"), day 3 pre-vein ECs ("d3pvus"), day 4 vein ECs ("d4vus"), and day 3-4 mesenchyme ("mes1"). Gene expression was measured by single-cell RNA-seq.

Table S2: **Bulk RNA-seq of hPSC-derived cell-types**
Count matrix of gene expression of day-0 hPSCs ("H1"), day-1 mid primitive streak ("D1_PS"), day-2 lateral mesoderm ("D2_DLM"), day 3 artery ECs, CD144+ FACS-purified ("D3_Artery"), day 3 pre-vein ECs, CD144+ FACS-purified ("D3_Prevein"), and day 4 vein ECs, CD144+ FACS-purified ("D4_Vein"). Gene expression was measured by bulk-population RNA-seq, and is quantified in $\log_2$ counts per million (CPM) units.

Table S3: **Single-cell RNA-seq of E9.5 mouse embryo ECs *in vivo***
Differentially expressed genes that distinguish 4 different types of ECs identified in the E9.5 mouse embryo. Raw single-cell RNA-sequencing data were obtained from a previous study[65]. We provisionally annotated the following clusters based on expression of characteristic marker genes: 0 (vein-like 1 ECs), 1 (other ECs), 2 (artery ECs), and 3 (vein-like 2 ECs).

Table S4: **Single-cell RNA-seq of gene expression changes upon triple *SOXF* knockdown during artery EC differentiation**
Genes that are differentially expressed between artery ECs generated from control vs. *SOX7/SOX17/SOX18*-CRISPRi hPSCs. Positive fold change indicates higher expression in control artery ECs, and $\log_2$ fold change is shown. Gene expression was measured by single-cell RNA-seq.

Table S5: **Single-cell RNA-seq of gene expression changes upon triple *SOXF* knockdown during vein EC differentiation**
Genes that are differentially expressed between vein ECs generated from control vs. *SOX7/SOX17/SOX18*-CRISPRi hPSCs. Positive fold change indicates higher expression in control vein ECs, and $\log_2$ fold change is shown. Gene expression was measured by single-cell RNA-seq.

Table S6: **Cell-surface marker screening of hPSC-derived cell-types**
For each hPSC-derived cell-type, the number indicates the percentage of cells that expressed a given cell-surface marker by high-throughput flow cytometry. Day 3 artery EC and day 4 vein EC populations were pre-gated on the CD144+ endothelial subset before quantifying the expression of other cell-surface markers. This was done to avoid being confounded by CD144- non-endothelial cells in the culture.

Table S7: **Differentially accessible genomic elements in hPSC-derived artery vs. vein ECs, as assayed by OmniATAC-seq**
Differentially accessible genomic elements in hPSC-derived artery vs. vein ECs, as assayed by OmniATAC-seq. Fold change indicates accessibility in vein ECs relative to artery ECs (i.e.,

bioRxiv preprint doi: https://doi.org/10.1101/2025.10.11.681838; this version posted October 12, 2025. The copyright holder for this preprint (which was not certified by peer review) is the author/funder, who has granted bioRxiv a license to display the preprint in perpetuity. It is made available under aCC-BY 4.0 International license.

a positive value indicates that a genomic element is more accessible in vein ECs, whereas a negative value indicates greater chromatin accessibility in artery ECs). The gene closest to each genomic element is also indicated.

Table S8: Bulk RNA-seq of hPSC-derived artery and vein ECs infected by Risk Group 4 viruses

Tab 1: count matrix of gene expression of hPSC-derived artery and vein ECs infected with either Sendai, Andes, Nipah, or Ebola viruses at 6, 12, 24, and 48 hours post-infection. Uninfected cells are designated as "Mock". Each condition was assessed in triplicate. Tab 2: sample annotations. Gene expression was measured by bulk-population RNA-seq, and count units are shown.
